# Supplementary material for: Synthesis, In Silico and In Vivo Toxicity Assessment of Functionalized Pyridophenanthridinones via Sequential MW-Assisted Intramolecular Friedel-Crafts Alkylation and Direct C–H Arylation
Source: Molecules. 2022 Nov 22;27(23):8112. doi: 10.3390/molecules27238112 (PMC9741109; doi:10.3390/molecules27238112)
Supplement: Supplementary file 1 [file molecules-27-08112-s001.zip › molecules-2005088-supplementary.pdf]

Synthesis, *in silico* and *in vivo* toxicity assessment of functionalized pyridophenanthridinones obtained via sequential MW-assisted intramolecular Friedel-Crafts alkylation and direct C–H arylation

Marlyn C. Ortiz Villamizar[1], Carlos E. Puerto Galvis[1], Silvia A. Pedraza Rodríguez[1], Fedor I. Zubkov[2] and Vladimir V. Kouznetsov[1,*]

[1] *Laboratorio de Química Orgánica y Biomolecular, CMN, Universidad Industrial de Santander. Parque Tecnológico Guatiguará, Km 2 Vía Refugio, Piedecuesta 681011, Colombia.*
[2] *Department of Organic Chemistry, Faculty of Science, RUDN University, 6 Miklukho-Maklaya St., Moscow 117198, Russian Federation*

* *kouznet@uis.edu.co*.

SUPPORTING INFORMATION

List of contents **Pages**

**1.**	Optimization of reaction conditions for the synthesis of *N*-arylcinnamamides **8a-f**	SI-2
**2.**	Characterization data of all synthesized *N*-arylcinnamamides **8a-f**	SI-2
**3.**	Characterization data of all synthesized *N*-(2-bromobenzyl)-*N*-phenylcinnamamide **7a-f**	SI-5
**4.**	Optimization of reaction conditions for the synthesis of *N*-benzyl-4-phenyl-3,4-dihydroquinolin-2(1*H*)-ones **6a-f**	SI-8
**5.**	Characterization data of all synthesized *N*-benzyl-4-phenyl-3,4-dihydroquinolin-2(1*H*)-ones **6a-f**	SI-9
**6.**	Characterization data of all synthesized pyrido[3,2,1-*de*]phenanthridin-6-ones **4a-f**	SI-12
**7.**	^{1}H and ^{13}C NMR spectra of all synthesized *N*-arylcinnamamides **8a-f** (Figures S1-S16)	SI-16
**8.**	^{1}H and ^{13}C NMR spectra of all synthesized *N*-(2-bromobenzyl)-*N*-phenylcinnamamide **7a-f** (Figures S17-S32)	SI-24
**9.**	^{1}H and ^{13}C NMR spectra of all synthesized *N*-benzyl-4-phenyl-3,4-dihydroquinolin-2(1*H*)-ones **6a-f** (Figures S33-S48)	SI-32
**10.**	^{1}H and ^{13}C NMR spectra of all synthesized pyrido[3,2,1-*de*]phenanthridin-6-ones **4a-f** (Figures S49-S65)	SI-40

1. Optimization of reaction conditions for the synthesis of *N*-arylcinnamamides 8a-f

Table S1. Optimization of reaction conditions for the synthesis of *N*-arylcinnamamides **8a-f**[a]

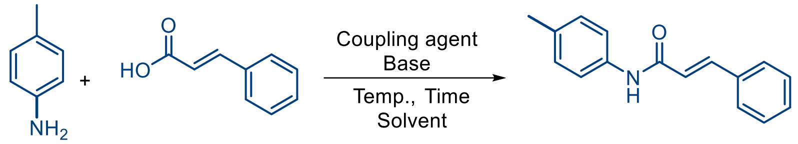

Entry	Solvent (mL)	Coupling agent (equiv)	Base (equiv)	Time (h/min)	Temp. (°C)	Yield (%)[b]
**1**[c]	DMF (15)	TBTU (1)	Et_3N (1)	48 h	r.t.	36
**2**[d]	-	SiO_2 (5)	-	80 min	130	NR[e]
**3**[d]	-	SiO_2 (5)	-	10 min	160	28
**4**[d]	[Bmim]PF_6	-	-	15 min	240	NR[e]
**5**[d]	DMF (8)	DCC (1)	-	1 min	145	27
**6**[d]	DMF (8)	DCC (1)	-	10 min	145	45
**7**[d]	DMF (8)	TBTU (1)	Et_3N (1)	10 min	80	77
**8**[d]	DMF (8)	TBTU (1)	Et_3N (1)	10 min	100	93

[a] *Reaction conditions:* **11b** (1 equiv), **10** (1 equiv), coupling agent, Base, temperature, time, solvent. [b] Isolated yield after column chromatography over SiO_2. [c] Reaction performed under conventional conditions. [d] Reaction assisted by microwave irradiation on a Biotage® Initiator+. [e] NR: No Reaction.

2. Characterization data of all synthesized *N*-arylcinnamamides 8a-f

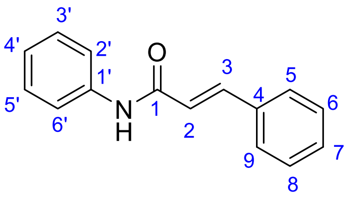

***N*-Phenylcinnamamide** (**8a**) was prepared according to the general procedure from aniline **11a** (93.13 mg, 1 mmol) and cinnamic acid **10** (148.16 mg, 1 mmol) in DMF. 198.71 mg (0.89 mmol) of a white solid was obtained, with a yield of 89 %. R_f = 0.27 (5: 1 petroleum ether / ethyl acetate); m.p. = 156 °C. **IR** (KBr, ν_{max}/cm^{-1}): 3270 ν(N-H), 3054 ν(=C-H), 1650 ν(C=O), 1604 ν(C=C), 1342 ν(C-N). **^{1}H NMR** (400 MHz, $CDCl_3$) δ $_{(ppm)}$: 7.90 (s, 1H, NH), 7.75 (d, *J* = 15.5 Hz, 1H, 3-CH), 7.66 (d, *J* = 7.1 Hz, 2H, 2'- and 6'-H_{Ar}), 7.47 (dd, *J* = 7.2, 2.4 Hz, 5- and 9-H_{Ar}), 7.37–7.28 (m, 5H, 3-', 5-', 6-, 7- and 8-H_{Ar}), 7.12 (t, *J* = 7.4 Hz, 1H, 4'-H_{Ar}), 6.62 (d, *J* = 15.5 Hz, 1H, 2-CH). **^{13}C NMR** (101 MHz, $CDCl_3$) $\delta_{(ppm)}$: 164.5 (1-CO), 142.3 (3-CH), 138.2 (1'-C_{Ar}), 134.6 (4-C_{Ar}), 129.9 (3-' and 5'-C_{Ar}), 129.1 (6- and 8-C_{Ar}), 128.9 (5- and 9-C_{Ar}), 128.0 (7-C_{Ar}), 124.5 (4'-C_{Ar}), 121.1 (2-CH), 120.3 (2'- and 6'-C_{Ar}). **HRMS** (ESI+): *m/z*: calcd for $C_{15}H_{13}NO$ ($[M+H]^+$) 224.1069, found: 224.1073.

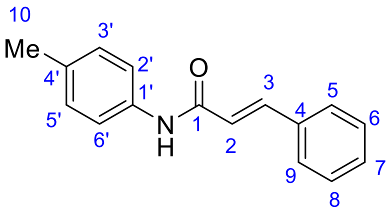

***N*-(p-Methylphenyl)cinnamamide** (**8b**) was prepared according to the general procedure from *p*-toluidine **11b** (107.17 mg, 1 mmol), and cinnamic acid **10** (148.16 mg, 1 mmol) in DMF. 220.69 mg (0.93 mmol) of a white solid was obtained, with a yield of 93%; R_f= 0.33 (5: 1 petroleum ether / ethyl acetate); m.p. = 168–170 °C. **IR**

(KBr, ν_{max}/cm^{-1}): 3255 ν(N-H), 3054 ν(=C-H), 1666 ν(C=O), 1604 ν(C=C), 1342 ν(C-N). **^{1}H NMR** (400 MHz, $CDCl_3$) $\delta_{(ppm)}$: 7.88 (s, 1H, NH), 7.73 (d, *J* = 15.5 Hz, 1H, 3-CH), 7.53 (d, *J*= 7.5 Hz, 2H, 2-’ and 6’-H_{Ar}), 7.46 (dd, *J* = 7.1, 2.4 Hz, 2H, 5- and 9-H_{Ar}), 7.36–7.28 (m, 3H, 6-, 7- and 8-H_{Ar}), 7.12 (d, *J* = 8.2 Hz, 2H, 3’- and 5’-H_{Ar}), 6.61 (d, *J* = 15.5 Hz, 1H, 2-CH), 2.31 (s, 3H, 10-CH_3). **^{13}C NMR** (101 MHz, $CDCl_3$) $\delta_{(ppm)}$: 164.2 (1-CO), 142.1 (3-CH), 135.6 (1’-C_{Ar}), 134.8 (4-C_{Ar}), 134.1 (4’-C_{Ar}), 129.9 (3’- and 5’-C_{Ar}), 129.6 (7-C_{Ar}), 128.9 (6- and 8-C_{Ar}), 128.0 (5- and 9-C_{Ar}), 121.2 (2-CH), 120.3 (2’- and 6’-C_{Ar}), 21.0 (10-CH_3). **DEPT 135** (101 MHz, $CDCl_3$) $\delta_{(ppm)}$: 142.0 (3-CH), 129.8 (3-’ and 5’-C_{Ar}), 129.6 (7-C_{Ar}), 128.8 (5- and 9-C_{Ar}), 128.0 (6- and 8-C_{Ar}), 121.2 (2’- and 6’-C_{Ar}), 120.4 (2-CH), 21.0 (10-CH_3). Correlation **COSY** [δH/δH]: 7.73/6.61 [3-CH/2-CH], 7.53/7.12 [2-’ and 6’-H_{Ar} / 3-’ and 5’-H_{Ar}], 7.46/7.36-7.28 [5- and 9-H_{Ar} / 6-, 7- and 8-H_{Ar}]. Correlation **HSQC**, [δH/δC]: 7.73/142.1 [3-CH/3-CH], 7.53/ 120,3 [2-’ and 6’-H_{Ar} /2’- and 6’-C_{Ar}], 7.46/128.0 [5- and 9-H_{Ar}/5 and 9-C_{Ar}], 7.36-7.28/128.9/129.6[6, 7 and 8-H_{Ar}/6, 7- and 8-C_{Ar}], 7.12/129.9 [3’- and 5’-H_{Ar}/3’- and 5’-C_{Ar}], 6.61/121.2 [2-CH/2-CH], 2.31/21.0 [10-CH_3/10-CH_3]. Correlation **HMBC** [δH/δC]: 2.31/129.9/134.1 [10-CH/3’ and 5’-C_{Ar}/4’-C_{Ar}], 6.61/134.8/142.1/164.2 [2-CH/4-C_{Ar}/3-CH/1-CO], 7.12/21,0/129.9/ 135.6 [3’ and 5’-H_{Ar} / 10-CH_3 / 3-’ and 5’- C_{Ar}/ 1’-C_{Ar}], 7.36–7.28/128.9/134.8 [6-, 7- and 8-H_{Ar}/5- and 9-C_{Ar}/ 4-C_{Ar}], 7.46/129.6/142.1/ 128.0 [5 and 9-H_{Ar}/7-C_{Ar}/3-CH/ 6- and 8-C_{Ar}], 7.53/134.1 [2-’ and 6’-H_{Ar}/4-C_{Ar}], 7.73/121.2/128.9/134.8/164.2 [3-CH/2-CH/5 and 9-C_{Ar}/4-C_{Ar}/1-CO], 7.88/121.2/142.1/164.2 [NH/2-CH/3-CH/1-CO]. **HRMS** (ESI+): *m/z*: calcd for $C_{16}H_{15}NO$ ($[M+H]^+$) 238.1226, found: 238.1231.

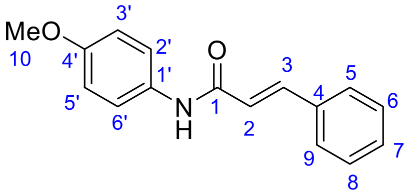

***N*-(4-Methoxyphenyl)cinnamamide** (**8c**) was prepared according to the general procedure from anisidine **11c** (123.15 mg, 1 mmol) and cinnamic acid **10** (148.16 mg, 1 mmol) in DMF. 222.90 mg (0.88 mmol) of a white solid were obtained, with a yield of 88%; R_f = 0.20 (5:1 petroleum ether/ethyl acetate); m.p. = 155–156 °C. **IR** (KBr, ν_{max}/cm^{-1}): 3301 ν(N-H), 3054 ν(=C-H), 1666 ν(C=O), 1619 ν(C=C), 1342 ν(C-N), 1234 ν(C-O). **^{1}H NMR** (400 MHz, $CDCl_3$) $\delta_{(ppm)}$: 8.16 (s, 1H, NH), 7.71 (d, *J* = 15.6 Hz, 1H, 3-CH), 7.55 (d, *J* = 8.8 Hz, 2H, 2’- and 6’-H_{Ar}), 7.45–7.38 (m, 2H, 5- and 9-H_{Ar}), 7.34–7.26 (m, 3H, 6-, 7- and 8-H_{Ar}), 6.83 (d, *J* = 8.9 Hz, 2H, 3-’ and 5’-H_{Ar}), 6.63 (d, *J* = 15.5 Hz, 1H, 2-CH), 3.74 (s, 3H, 10-CH_3). **^{13}C NMR** (101 MHz, $CDCl_3$) $\delta_{(ppm)}$: 164.2 (1-CO), 156.5 (4’-C_{Ar}), 141.9 (3-CH), 134.8 (4-C_{Ar}), 131.3 (1’-C_{Ar}), 129.9 (7-C_{Ar}), 128.9 (6- and 8-C_{Ar}), 128.0 (5- and 9-C_{Ar}), 122.0 (2’- and 6’-C_{Ar}), 121.1 (2-CH), 114.2 (3’- and 5’-C_{Ar}), 55.5 (10-CH_3). **HRMS** (ESI+): *m/z*: calcd for $C_{16}H_{15}NO_2$ ($[M+H]^+$) 254.1175, found: 254.1180.

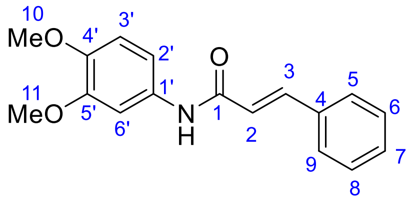

***N*-(3,4-Dimethoxyphenyl)cinnamamide** **(8d)** was prepared according to the general procedure from 3,4-dimethoxyaniline **1e** (153.18 mg, 1 mmol) and cinnamic acid (148.16 mg, 1 mmol) in DMF. 229.5 mg (0.81 mmol) of a white solid were obtained, with a yield of 81%; R_f = 0.50 (5:1 petroleum ether/ethyl acetate); m.p. = 147-150 °C. **IR** (KBr, ν_{max}/cm^{-1}): 3311 ν(N-H), 3055 ν(=C-H), 1665 ν(C=O), 1617 ν(C=C), 1343 ν(C-N), 1235 ν(C-O). **^{1}H NMR** (400 MHz, $CDCl_3$) $\delta_{(ppm)}$: 8.24 (s, 1H, NH), 7.73 (d, *J* = 15.6 Hz, 1H, 3-CH), 7.50 (s, 1H, 6'-H_{Ar}), 7.44–7.41 (m, 2H, 5- and 9-H_{Ar}), 7.32–7.29 (m, 3H, 6-, 7-, and 8-H_{Ar}), 7.06 (d, *J* = 7.9 Hz, 1H, 3'-H_{Ar}), 6.77 (d, *J* = 8.7 Hz, 1H, 2'-H_{Ar}), 6.64 (d, *J* = 15.6 Hz, 1H, 2-CH), 3.82 (s, 6H, 10- and 11-CH_3). **^{13}C NMR** (101 MHz, $CDCl_3$) $\delta_{(ppm)}$: 164.3 (1-CO), 149.1 (4'-C_{Ar}), 145.9 (5'-C_{Ar}), 142.0 (3-CH), 134.7 (4-C_{Ar}), 132.0 (1'-C_{Ar}), 129.9 (7-C_{Ar}), 128.9 (6- and 8-C_{Ar}), 127.9 (5-and 9-C_{Ar}), 121.1 (2-CH), 112.2 (6'-C_{Ar}), 111.4 (3'-C_{Ar}), 105.1 (2'-C_{Ar}), 56.1 (11-CH_3), 55.9 (10-CH_3). **HRMS** (ESI+): *m/z*: calcd for $C_{17}H_{17}NO_3$ ($[M+H]^+$) 284.1281, found: 284.1287.

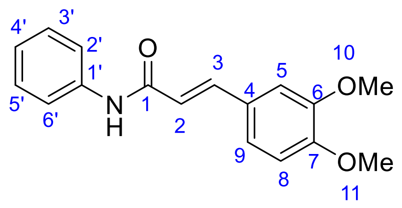

***(E)*-3-(3,4-Dimethoxyphenyl)-*N*-phenylacrylamide** **(8e)** was prepared according to the general procedure from aniline **1a** (93.13 mg, 1 mmol) and 3,4-dimethoxybenzoic acid (182.17 mg, 1 mmol) in DMF. 240.85 mg (0.85 mmol) of a white solid were obtained, with a yield of 85%; R_f = 0.20 (5:1 petroleum ether/ethyl acetate); m.p. = 120-124 °C. **IR** (KBr, ν_{max}/cm^{-1}): 3303 ν(N-H), 3056 ν(=C-H), 1666 ν(C=O), 1618 ν(C=C), 1343 ν(C-N), 1234 ν(C-O). **^{1}H NMR** (400 MHz, $CDCl_3$) $\delta_{(ppm)}$: 9.09 (s, 1H, NH), 7.73 (d, *J* = 8.2 Hz, 2H, 2'- and 6'-H_{Ar}), 7.67 (d, *J* = 15.5 Hz, 1H, 3-CH), 7.30–7.22 (m, 2H, 3'- and 5'-H_{Ar}), 7.08–7.03 (m, 1H, 4'-H_{Ar}), 6.92 (dd, *J*= 8.4, 2.1 Hz, 1H, 9-H_{Ar}), 6.86 (d, *J* = 2.0 Hz, 1H, 5-H_{Ar}), 7.67 (d, *J* = 15.5 Hz, 1H, 2-CH), 6.68 (d, *J*= 8.4 Hz, 1H, 8-H_{Ar}), 3.80 (s, 3H, 11-CH_3), 3.65 (s, 3H, 10-CH_3).**^{13}C NMR** (101 MHz, $CDCl_3$) $\delta_{(ppm)}$: 165.1 (1-CO), 150.5 (7-C_{Ar}), 148.8 (6-C_{Ar}), 141.7 (3-CH), 138.5 (4-C_{Ar}), 128.9 (2'- and 6'-C_{Ar}), 127.6 (1'-C_{Ar}), 124.1 (5-C_{Ar}), 121.8 (2-CH), 120.1 (8-C_{Ar}), 119.2 (9-C_{Ar}), 111.0 (3'- and 5'-C_{Ar}), 109.9 (4'-C_{Ar}), 55.7 (11-CH_3), 55.5 (10-CH_3). **HRMS** (ESI+): *m/z*: calcd for $C_{17}H_{17}NO_3$ ($[M+H]^+$) 284.1281, found: 284.1290.

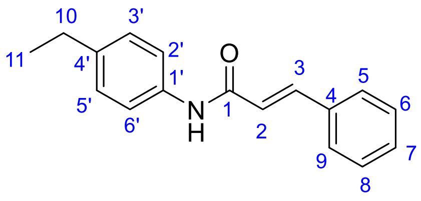

***N*-(4-Ethylphenyl)cinnamamide** **(8f)** was prepared according to the general procedure from 4-ethylaniline **1d** (121.18 mg, 1 mmol) and cinnamic acid (148.16 mg, 1 mmol) in DMF. 223.68 mg (0.89 mmol) of a white solid were obtained, with a yield of 89%; R_f = 0.37 (5:1 petroleum ether/ethyl acetate); m.p. = 146–147 °C. **IR** (KBr, ν_{max}/cm^{-1}): 3286 ν(N-H), 3054 ν(=C-H), 2962 ν(C-C), 1666 ν(C=O), 1619 ν(C=C), 1342 ν(C-N). **^{1}H NMR** (400 MHz, $CDCl_3$) $\delta_{(ppm)}$: 8.07 (s, 1H, NH), 7.73 (d, *J* = 15.5 Hz, 1H, 3-CH), 7.57 (d, *J* = 7.6 Hz, 2H,

2’- and 6’-H_{Ar}), 7.47–7.38 (m, 2H, 5- and 9-H_{Ar}), 7.35–7.27 (m, 3H, 6-, 7- and 8-H_{Ar}), 7.14 (d, *J* = 8.3 Hz, 2H, 3- and 5’-H_{Ar}), 6.64 (d, *J* = 15.5 Hz, 1H, 2-CH), 2.60 (q, *J*= 7.6 Hz, 2H, 10-CH_2), 1.21 (t, *J* = 7.6 Hz, 3H, 11-CH_3).**^{13}C NMR** (101 MHz, $CDCl_3$) $\delta_{(ppm)}$: 164.3 (1-CO), 142.1 (3-CH), 140.6 (4’-C_{Ar}), 135.8 (1’-C_{Ar}), 134.8 (4-C_{Ar}), 129.9 (7-C_{Ar}), 128.9 (6- and 8-C_{Ar}), 128.4 (5- and 9-C_{Ar}), 128.0 (3’- and 5’-C_{Ar}), 121.2 (2-CH), 120.4 (2’- and 6’-C_{Ar}), 28.4 (10-CH_2), 15.7 (11-CH_3). **HRMS** (ESI+): *m/z*: calcd for $C_{17}H_{17}NO$ ($[M+H]^+$) 252.1383, found: 252.1388.

3. Characterization data of all synthesized *N*-(2-bromobenzyl)-*N*-phenylcinnamamide 7a-f

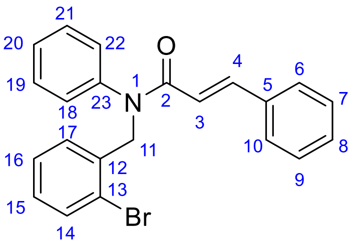

N-(2-Bromobenzyl)-N-phenylcinnamamide (**7a**) was prepared according to the general procedure from *N*-phenylcinnamamide **8a,** (223.27 mg, 1 mmol) and 2-bromobenzyl bromide, **12**, (374.89 mg, 1.5 mmol) in THF. 364.83 mg (0.93 mmol) of a white solid were obtained, with a yield of 93%. R_f = 0.47 (5:1 petroleum ether/ethyl acetate); m.p. = 125 °C. **IR** (KBr, ν_{max}/cm^{-1}): 3054 ν(=C-H), 2915 ν(C-C), 1650 ν(C=O), 1604 ν(C=C), 1388 ν(C-N). **^{1}H NMR** (400 MHz, $CDCl_3$) $\delta_{(ppm)}$: 7.77 (d, *J* = 15.5 Hz, 1H, 4-CH), 7.49 (dd, *J* = 8.0, 1.1 Hz, 1H, 14-H_{Ar}), 7.43 (dd, *J* = 7.7, 1.5 Hz, 1H, 18-H_{Ar}), 7.38–7.24 (m, 9H, 6-, 7-, 8-, 9-, 10-, 19-, 20-, 21- and 22-H_{Ar}), 7.17–7.14 (m, 2H, 16- and 17-H_{Ar}), 7.10 (td, *J* = 7.9, 1.7 Hz, 1H, 15-H_{Ar}), 6.42 (d, *J* = 15.5 Hz, 1H, 3-CH), 5.19 (s, 2H, 11-CH). **^{13}C NMR** (101 MHz, $CDCl_3$) $\delta_{(ppm)}$: 166.3 (2-CO), 142.7 (4-CH), 141.9 (12-C_{Ar}), 136.4 (23-C_{Ar}), 135.1 (5-C_{Ar}), 132.8 (14-C_{Ar}), 129.8 (8-C_{Ar}), 129.7 (7- and 9-C_{Ar}), 129.6 (18- and 22-C_{Ar}), 128.8 (16-C_{Ar}), 128.8 (6- and 10-C_{Ar}), 128.1 (15-C_{Ar}), 128.0 (19- and 21-C_{Ar}), 128.0 (17-C_{Ar}), 127.6 (20-C_{Ar}), 123.6 (13-C_{Ar}), 118.5 (3-CH), 53.1 (11-CH_2). **HRMS** (ESI+): *m/z*: calcd for $C_{22}H_{18}BrNO$ ($[M+H]^+$) 392.0644, found: 392.0652.

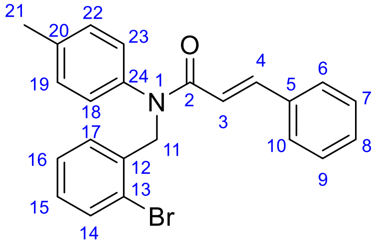

N-(2-Bromobenzyl)-N-(p-tolyl)lcinnamamide (**7b**) was prepared according to the general procedure from *N*-phenylcinnamamide **8b,** (237.30 mg, 1 mmol) and 2-bromobenzyl bromide, **12**, (374.89 mg, 1.5 mmol) in THF. 337.25 mg (0.83 mmol) of a white solid were obtained, with a yield of 83%. R_f = 0.50 (5:1 petroleum ether/ethyl acetate); m.p. = 153 - 155 °C. **IR** (KBr, ν_{max}/cm^{-1}): 3054 ν(=C-H), 2915 ν(C-C), 1650 ν(C=O), 1619 ν(C=C), 1388 ν(C-N), 570.84 ν(C-Br). **^{1}H NMR** (400 MHz, $CDCl_3$) $\delta_{(ppm)}$: 7.79 (d, *J* = 15.6 Hz, 1H, 4-CH), 7.51 (d, *J* = 8.0 Hz, 1H, 14-H_{Ar}), 7.44 (dd, *J* = 7.7, 1.3 Hz, 1H, 17-H_{Ar}), 7.40–7.24 (m, 6H, 6-, 7-, 8-, 9-, 10- and 16-H_{Ar}), 7.17 (d, *J* = 8.3 Hz, 2H, 18- and 23-H_{Ar}), 7.12 (td, *J* = 7.8, 1.5 Hz, 1H, 15-H_{Ar}), 7.05 (d, *J* = 8.2 Hz, 2H, 19- and 22-H_{Ar}), 6.47 (d, *J* = 15.6 Hz, 1H, 3-CH), 5.18 (s, 2H, 11-CH_2), 2.39 (s, 3H, 21-CH_3). **^{13}C NMR** (101 MHz, $CDCl_3$) $\delta_{(ppm)}$: 166.4 (2-CO), 142.5 (4-CH), 139.3 (12-C_{Ar}), 137.8 (20-C_{Ar}), 136.5 (24-C_{Ar}), 135.2 (5-C_{Ar}), 132.8 (14-C_{Ar}), 130.2 (17-, 18- and 23-C_{Ar}),

129.8 (8-C_{Ar}), 129.7 (7- and 9-C_{Ar}), 128.8 (6- and 10-C_{Ar}), 128.0 (16-C_{Ar}), 127.8 (15-C_{Ar}), 127.6 (19- and 22-C_{Ar}), 123.6 (13-C_{Ar}), 118.6 (3-CH), 53.1 (11-CH_2), 21.2 (21-CH_3). **DEPT 135** (101 MHz, $CDCl_3$) $\delta_{(ppm)}$: 142.5 (4-CH), 132.7 (17-C_{Ar}), 130.2 (18- and 23-C_{Ar}), 129.7 (8-C_{Ar}), 129.7 (7- and 9-C_{Ar}), 128.78 (6- and 10-C_{Ar}), 128.0 (16-C_{Ar}), 127.8 (15-C_{Ar}), 127.6 (19- and 22-C_{Ar}), 118.6 (3-CH), 53.1 (11-CH_2), 21.2 (21-CH_3). Correlation **COSY** [δH/δH]: 6.47/7.79 [3-CH/4-CH], 7.05/7.17 [18- and 23-H_{Ar}/19- and 22-H_{Ar}], 7.12/7.51 [15-H_{Ar}/14-H_{Ar}], 7.44/7.40–7.24 [17-H_{Ar}/16-H_{Ar}]. Correlation **HSQC** [δH/δC]: 2.39/21.2 [21-CH_3/21-CH_3], 5.18/53.1 [11-CH_2], 6.47/118.6 [3-CH/3-CH], 7.05/127.6 [19 and 22-H_{Ar}/19- and 22-C_{Ar}], 7.12/127.8 [15-H_{Ar}/15-C_{Ar}], 7.17/130.2 [18- and 23-H_{Ar}/19 and 22-C_{Ar}], 7.40 – 7.24/129.7/128.0 [7-, 9- and 16-H_{Ar}/7- and 9-C_{Ar}/16-C_{Ar}], 7.44/130.2 [17-H_{Ar}/17-C_{Ar}], 7.51/132.8 [14-H_{Ar}/14-C_{Ar}], 7.79/142.5 [4-CH/4-CH]. Correlation **HMBC** [δH/δC]: 2.39/130.21/137.88 [21-CH_3/18 and 23-C_{Ar}/20-C_{Ar}], 5.18/123.6/130.2/136.5/139.3/166.4 [11-CH_2/13-C_{Ar}/18 and 23-C_{Ar}/24-C_{Ar}/12-C_{Ar}/2-CO], 6.47/135.2/166.4 [3-CH/5-C_{Ar}/2-CO], 7.05/127.6/ 137.8 [19- and 22-H_{Ar}/19- and 22-C_{Ar}/20-C_{Ar}], 7.12/123.6/130.2 [15-H_{Ar}/13-C_{Ar}/17-C_{Ar}], 7.17/21.2/130.2/137.8 [18- and 23-H_{Ar}/21-CH_3/18- and 23-C_{Ar}/20-C_{Ar}], 7.40 – 7.24/127.8/132.8/ 135.2/ 129.7 [6, 7, 8, 9, 10 and 16-H_{Ar}/15-C_{Ar}/14-C_{Ar}/5-C_{Ar}/7- and 9-C_{Ar}], 7.44/53.1/123.6/128.0 [17-H_{Ar}/11-CH_2/13-C_{Ar}/16-C_{Ar}], 7.51/127.8/123.6 [14-H_{Ar}/15-C_{Ar}/13-C_{Ar}], 7.79/118.6/128.8/166.4/ 135.2 [4-CH/3-CH/6 and 10-C_{Ar}/2-CO/5-C_{Ar}]. **HRMS** (ESI+): *m/z*: calcd for $C_{23}H_{20}BrNO$ ($[M+H]^+$) 406.0801, found: 406.0809.

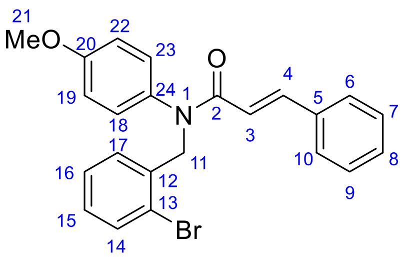

***N*-(2-Bromobenzyl)-*N*-(4-methoxyphenyl)cinnamamide** (**7c**) was prepared according to the general procedure from *N*-phenylcinnamamide **8c,** (253.30 mg, 1 mmol) and 2-bromobenzyl bromide, **12**, (374.89 mg, 1.5 mmol) in THF. 413.87 mg (0.98 mmol) of a white solid were obtained, with a yield of 98%. R_f = 0.50 (5:1 petroleum ether/ethyl acetate); m.p. = 101-105 °C. **IR** (KBr, ν_{max}/cm^{-1}): 3054 ν(=C-H), 2962 ν(C-C), 1650 ν(C=O), 1619 ν(C=C), 1388 ν(C-N). **^{1}H NMR** (400 MHz, $CDCl_3$) $\delta_{(ppm)}$: 7.75 (d, *J* = 15.6 Hz, 1H, 4-CH), 7.48 (dd, *J* = 8.0, 0.9 Hz, 1H, 14-H_{Ar}), 7.41 (dd, *J* = 7.7, 1.5 Hz, 1H, 17-H_{Ar}), 7.36–7.23 (m, 6H, 6, 7, 8, 9, 10, 16-H_{Ar}), 7.09 (td, *J*= 7.8, 1.6 Hz, 1H, 15-H_{Ar}), 7.04 (d, *J* = 8.9 Hz, 2H, 18- and 23-H_{Ar}), 6.85 (d, *J* = 8.9 Hz, 2H, 19- and 22-H_{Ar}), 6.41 (d, *J* = 15.6 Hz, 1H, 3-CH), 5.14 (s, 2H, 11-CH_2), 3.81 (s, 3H, 21-CH_3). **^{13}C NMR** (101 MHz, $CDCl_3$) $\delta_{(ppm)}$: 166.5 (2-CO), 159.0 (20-C_{Ar}), 142.5 (4-CH), 136.5 (12-C_{Ar}), 135.2 (5-C_{Ar}), 134.5 (24-C_{Ar}), 132.8 (14-C_{Ar}), 130.1 (17-C_{Ar}), 129.7 (8-C_{Ar}), 129.4 (7- and 9-C_{Ar}), 128.8 (16-C_{Ar}), 128.8 (6- and 10-C_{Ar}), 128.0 (18- and 23-C_{Ar}), 127.6 (15-C_{Ar}), 123.8 (13-C_{Ar}), 118.5 (3-CH), 114.6 (19- and 22-C_{Ar}), 55.5 (21-CH_3), 53.1 (11-CH_2). **HRMS** (ESI+): *m/z*: calcd for $C_{23}H_{20}BrNO_2$ ($[M+H]^+$) 422.0750, found: 422.0758.

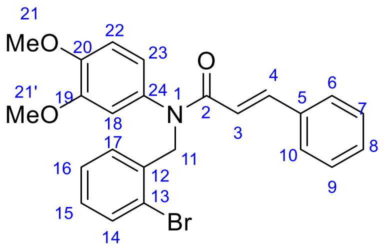

***N*-(2-bromobenzyl)-*N*-(3,4-dimethoxyphenyl) cinnamamide (7d)** was prepared according to the general procedure from *N*-(3,4-dimethoxyphenyl)cinnamamide **8d**, (283.33 mg, 1 mmol) and 2-bromobenzyl bromide, **12**, (374.89 mg, 1.5 mmol) in THF. 425.19 mg (0. 94 mmol) of a non-color solid were obtained, with a yield of 94%. R_f = 0.56 (5:1 petroleum ether/ethyl acetate); m.p. = 115-117 °C. **IR** (KBr, ν_{max}/cm^{-1}): 3053 ν(=C-H), 2964 ν(C-C), 1655 ν(C=O), 1620 ν(C=C), 1386 ν(C-N). **^{1}H NMR** (400 MHz, $CDCl_3$) $\delta_{(ppm)}$: 7.75 (d, *J*= 15.6 Hz, 1H, 4-CH), 7.48 (dd, *J* = 8.0, 1.4 Hz, 1H, 17-H_{Ar}), 7.42 (dd, *J* = 7.7, 1.7 Hz, 1H, 14-H_{Ar}), 7.36–7.27 (m, 5H, 7, 8, 9, 10, and16-H_{Ar}), 7.25 (dd, *J* = 7.6, 1.3 Hz, 1H, 6-H_{Ar}), 7.09 (td, *J* = 7.5, 1.7 Hz, 1H, 15-H_{Ar}), 6.79 (d, *J* = 8.5 Hz, 1H, 22-H_{Ar}), 6.68 (dd, *J* = 8.4, 2.4 Hz, 1H, 23-H_{Ar}), 6.56 (d, *J* = 2.4 Hz, 1H, 18-H_{Ar}), 6.42 (d, *J* = 15.6 Hz, 1H, 3-CH), 5.16 (s, 2H, 11-CH_2), 3.89 (s, 3H, 21’-CH_3), 3.73 (s, 3H, 21-CH_3). **^{13}C NMR** (101 MHz, $CDCl_3$) $\delta_{(ppm)}$: 166.4 (2-CO), 149.3 (20-C_{Ar}), 148.7 (19-C_{Ar}), 142.5 (4-CH), 136.7 (12-CAr), 135.2 (5-C_{Ar}), 134.5 (24-C_{Ar}), 132.7 (14-C_{Ar}), 130.5 (17-C_{Ar}), 129.7 (8-C_{Ar}), 128.9 (16-C_{Ar}), 128.8 (6- and 10-C_{Ar}), 128.0 (7- and 9-C_{Ar}), 127.6 (15-C_{Ar}), 124.0 (13-C_{Ar}), 120.8 (18-C_{Ar}), 118.5 (3-CH), 111.5 (22-C_{Ar}), 111.1 (23-C_{Ar}), 56.0 (21’- and 21-CH_3), 52.8 (11-CH_2). **HRMS** (ESI+): *m/z*: calcd for $C_{24}H_{22}BrNO_3$ ($[M+H]^+$) 452.0855, found: 452.0864.

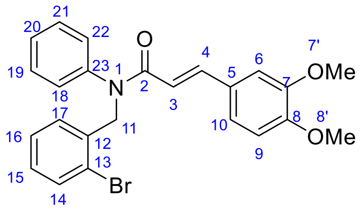

(*E*)-*N*-(2-bromobenzyl)-3-(3,4-dimethoxyphenyl)-*N*-phenylacrylamide (7e) was prepared according to the general procedure from (*E*)-3-(3,4-dimethoxyphenyl)-N-phenylacrylamide **8e**, (253.30 mg, 1 mmol) and 2-bromobenzyl bromide, **12**, (374.89 mg, 1.5 mmol) in THF. 413.87 mg (0.98 mmol) of a yellow liquid were obtained, with a yield of 98%. R_f = 0.50 (5:1 petroleum ether/ethyl acetate). **IR** (KBr, ν_{max}/cm^{-1}): 3056 ν(=C-H), 2964 ν(C-C), 1658 ν(C=O), 1621 ν(C=C), 1385 ν(C-N). **^{1}H NMR** (400 MHz, $CDCl_3$) $\delta_{(ppm)}$: 7.69 (d, *J* = 15.4 Hz, 1H, 4-CH), 7.48 (dd, *J* = 8.0, 1.4 Hz, 1H, 14-H_{Ar}), 7.42 (dd, *J* = 7.7, 1.9 Hz, 1H, 17-H_{Ar}), 7.38–7.23 (m, 5H, 10, 16, 19, 20 and 21-H_{Ar}), 7.16 (dd, *J* = 8.2, 1.6 Hz, 2H, 18- and 22-H_{Ar}), 7.09 (td, *J* = 7.6, 1.8 Hz, 1H, 15-H_{Ar}), 6.95 (dd, *J* = 8.6, 2.1 Hz, 1H, 9-H_{Ar}), 6.80 (d, *J* = 3.0 Hz, 1H, 6-H_{Ar}), 6.27 (d, *J* = 15.4 Hz, 1H, 3-CH), 5.18 (s, 2H, 11-CH_2), 3.86 (s, 3H, 24’-CH_3), 3.80 (s, 3H, 24-CH_3).**^{13}C NMR** (101 MHz, $CDCl_3$) $\delta_{(ppm)}$: 166.6 (2-CO), 150.7 (8-C_{Ar}), 149.1 (7-C_{Ar}), 142.6 (4-CH), 142.2 (13-C_{Ar}), 136.6(12-C_{Ar}), 132.8 (14-C_{Ar}), 129.8 (17-C_{Ar}), 129.5(18- and 22-C_{Ar}), 128.8(19- and 21-C_{Ar}), 128.2 (23-C_{Ar}), 128.2 (16-C_{Ar}), 127.8 (20-C_{Ar}), 127.6 (15-C_{Ar}), 123.6 (13-C_{Ar}), 121.8(6-C_{Ar}), 116.6 (3-CH), 111.2 (9-C_{Ar}), 110.5 (10-C_{Ar}), 56.0 (7’-C_{Ar}), 55.9 (8’-C_{Ar}), 53.1 (11-CH_2). **HRMS** (ESI+): *m/z*: calcd for $C_{24}H_{22}BrNO_3$ ($[M+H]^+$) 452.0855, found: 452.0869.

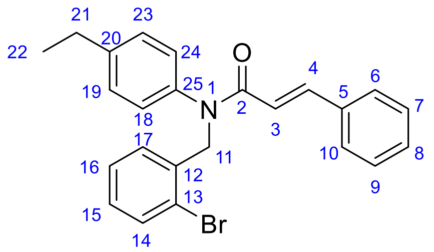

***N*-(2-Bromobenzyl)-*N*-(4-ethylphenyl)cinnamamide** (**7f**) was prepared according to the general procedure from *N*-(4-Ethylphenyl)cinnamamide **8f,** (251.32 mg, 1 mmol) and 2-bromobenzyl bromide, **12**, (374.89 mg, 1.5 mmol) in THF. 361.50 mg (0.86 mmol) of a white solid were obtained, with a yield of 86%. R_f = 0.53 (5:1 petroleum ether/ethyl acetate); m.p.= 136-137 °C. **IR** (KBr, ν_{max}/cm^{-1}): 3054 ν(=C-H), 2962 ν(C-C), 1650 ν(C=O), 1619 ν(C=C), 1388 ν(C-N). **^{1}H NMR** (400 MHz, $CDCl_3$) $\delta_{(ppm)}$: 7.77 (d, *J* = 15.6 Hz, 1H, 4-CH), 7.49 (dd, *J* = 8.0, 1.1 Hz, 1H, 14-H_{Ar}), 7.41 (dd, *J* = 7.7, 1.5 Hz, 1H, 17-H_{Ar}), 7.37–7.28 (m, 5H, 6-, 7-, 8-, 9-, 10-H_{Ar}), 7.26 (dd, *J* = 7.6, 1.1 Hz, 1H, 16-H_{Ar}), 7.17 (d, *J*= 8.5 Hz, 2H, 18- and 24-H_{Ar}), 7.10 (dd, *J* = 7.7, 1.5 Hz, 1H, 15-H_{Ar}), 7.06 (d, *J* = 8.4 Hz, 2H, 19- and 23-H_{Ar}), 6.46 (d, *J* = 15.6 Hz, 1H, 3-CH), 5.16 (s, 2H, 11-CH_2), 2.67 (q, *J* = 7.6 Hz, 2H, 21-CH_2), 1.25 (t, *J* = 7.6 Hz, 3H, 22-CH_3). **^{13}C NMR** (101 MHz, $CDCl_3$) $\delta_{(ppm)}$: 166.4 (2-CO), 144.1 (4-CH), 142.6 (20-C_{Ar}), 139.5 (12-C_{Ar}), 136.5 (25-C_{Ar}), 135.2 (5-C_{Ar}), 132.8 (14-C_{Ar}), 129.7 (7- and 9-C_{Ar}), 129.6 (8-C_{Ar}), 128.9 (19- and 23-C_{Ar}), 128.8 (6- and 10-C_{Ar}), 128.7 (17-C_{Ar}), 128.0 (16-C_{Ar}), 127.8 (15-C_{Ar}), 127.6 (18- and 24-C_{Ar}), 123.5 (13-C_{Ar}), 118.6 (3-CH), 53.3 (11-CH_2), 28.5 (21-CH_2), 15.4 (22-CH_3). **HRMS** (ESI+): *m/z*: calcd for $C_{24}H_{22}BrNO$ ($[M+H]^+$) 420.0957, found: 420.0965.

4. Optimization of reaction conditions for the synthesis of *N*-benzyl-4-phenyl-3,4-dihydroquinolin-2(1*H*)-ones 6a-f

Table S2. Optimization of reaction conditions for the synthesis of *N*-benzyl-4-phenyl-3,4-dihydroquinolin-2(1*H*)-ones ***6a-f***[a]

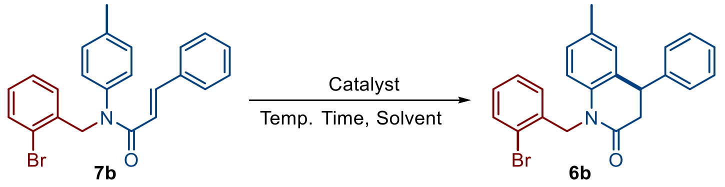

Entry	Catalyst (1 mL/mmol)	Solvent (mL)	Time (h/min)	Temp. (°C)	Yield (%)[b]
**1**[c]	TfOH	$CHCl_3$ (5)	24 h	65	22
**2**[d]	TfOH	$CHCl_3$ (5)	5 min	150	18
**3**[d]	TFA	-	10 min	80	NR[e]
**4**[d]	TFA	-	10 min	100	NR[e]
**5**[d]	TFA	-	60 min	100	Traces
**6**[d]	TFA	-	30 min	120	24
**7**[d]	TFA	-	30 min	130	38
**8**[d]	TFA	-	40 min	140	63

[a] *Reaction conditions:* **7b** (1 mmol), Catalyst (4 mL/mmol), temperature, time, solvent. [b] Isolated yield after column chromatography over SiO_2. [c] Reaction performed under conventional conditions. [d] Reaction assisted by microwave irradiation on a Biotage® Initiator+. [e] NR: No Reaction.

5. Characterization data of all synthesized *N*-benzyl-4-phenyl-3,4-dihydroquinolin-2(1*H*)-ones 6a-f

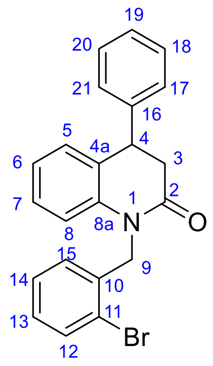

***1-(2-Bromobenzyl)-4-phenyl-3,4-dihydroquinolin-2(1H)-one* (6a)** was prepared according to the general procedure from *N*-(2-bromobenzyl)-*N*-phenylcinnamamide **7a**, (392.29 mg, 1mmol) and TFA (4 mL). 274.60 mg (0.70 mmol) of a white solid were obtained, with a yield of 70%; R_f = 0.37 (5:1 petroleum ether/ethyl acetate); m.p. = 122–124 °C. **IR** (KBr, ν_{max}/cm^{-1}): 3023 ν(=C-H), 1681 ν(C=O), 1373 ν(C-N), 694 ν(C-Br). **^{1}H NMR** (400 MHz, $CDCl_3$) δ(ppm): 7.59 (dd, *J* = 7.5, 1.6 Hz, 1H, 12-H_{Ar}), 7.39-7.33 (m, 2H, 21- and 17-H_{Ar}), 7.32–7.27 (m, 1H, 19-H_{Ar}), 7.24-7.19 (m, 2H, 20- and 18-H_{Ar}), 7.19–7.07 (m, 3H, 6-, 7- and 13-H_{Ar}), 7.03–6.96 (m, 2H, 14- and 15-H_{Ar}), 6.85 (dd, *J* = 7.4, 1.9 Hz, 1H, 5-H_{Ar}), 6.77 (d, *J* = 8.1 Hz, 1H, 8-H_{Ar}), 5.22 (q, *J* = 17.1 Hz, 2H, 9-CH_2), 4.35 (dd, *J* = 7.6, 6.1 Hz, 1H, 4-CH), 3.16 (dq, *J*= 15.8, 6.9 Hz, 2H, 3-CH_2). **^{13}C NMR** (101 MHz, $CDCl_3$) δ(ppm): 169.5 (2-CO), 140.9 (16-C_{Ar}), 139.3 (8a-C_{Ar}), 135.2 (10-C_{Ar}), 132.9 (12-C_{Ar}), 129.1 (4a-C_{Ar}), 129.0 (18 and 20-C_{Ar}), 128.7 (15-C_{Ar}), 128.5 (13-C_{Ar}), 128.2 (7-C_{Ar}), 127.9 (17- and 21-C_{Ar}), 127.8 (5-C_{Ar}), 127.3 (14-C_{Ar}), 127.2 (19-C_{Ar}), 123.4 (6-C_{Ar}), 122.6 (11-C_{Ar}), 115.7 (8-C_{Ar}), 46.7 (9-CH_2), 41.5 (4-CH), 38.8 (3-CH_2). **HRMS** (ESI+): *m/z*: calcd for $C_{22}H_{18}BrNO$ ([M+H]$^+$) 392.0644, found: 392.0652.

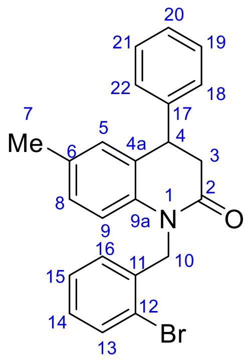

***1-(2-Bromobenzyl)-6-methyl-4-phenyl-3,4-dihydroquinolin-2(1H)-one*. (6b)** was prepared according to the general procedure from *N*-(2-bromobenzyl)-*N*-(*p*-tolyl)lcinnamamide (**7b**) (406.32 mg, 1 mmol) and TFA (4 mL). 268.17 mg (0.66 mmol) of a white solid were obtained, with a yield of 66%; R_f = 0.38 (5:1 petroleum ether/ethyl acetate); m.p. = 135 °C. **IR** (KBr, ν_{max}/cm^{-1}): 3023 ν(=C-H), 1681 ν(C=O), 1373 ν(C-N), 694 ν(C-Br). **^{1}H NMR** (400 MHz, $CDCl_3$) δ(ppm): 7.58 (dd, *J* = 7.5, 1.7 Hz, 1H, 13-H_{Ar}), 7.39–7.33 (m, 2H, 22- and 18-H_{Ar}), 7.32–7.27 (m, 1H, 20-H_{Ar}), 7.23–7.18 (m, 2H, 21 and 19-H_{Ar}), 7.11 (pd, *J* = 7.3, 1.7 Hz, 2H, 14- and 16-H_{Ar}), 6.96 (dd, *J* = 8.3, 1.9 Hz, 1H, 8-H_{Ar}), 6.84 (dd, *J* = 6.1, 1.5 Hz, 2H, 5- and 15-H_{Ar}), 6.67 (d, *J* = 8.3 Hz, 1H, 9-H_{Ar}), 5.29–5.11 (m, 2H, 10-CH_2), 4.30 (t, *J* = 6.7 Hz, 1H, 4-CH), 3.14 (dq, *J* = 15.8, 6.7 Hz, 2H, 3-CH_2), 2.22 (s, 3H, 7-CH_3). **^{13}C NMR** (101 MHz, $CDCl_3$) δ(ppm): 169.4 (2-CO), 141.1 (17-C_{Ar}), 136.9 (9a-C_{Ar}), 135.4 (11-C_{Ar}), 133.1 (6-C_{Ar}), 132.9 (13-C_{Ar}), 129.2 (14-C_{Ar}), 129.0 (21- and 19-C_{Ar}), 128.9 (4a-C_{Ar}), 128.7 (16-C_{Ar}), 128.6 (15-C_{Ar}), 127.9 (22 and 18-C_{Ar}), 127.7 (5-C_{Ar}), 127.3 (8-C_{Ar}), 127.2 (20-C_{Ar}), 122.6 (12-C_{Ar}), 115.6 (9-C_{Ar}), 46.7 (10-CH_2), 41.5 (4-CH), 39.0 (3-CH_2), 20.7 (7-CH_3). **HRMS** (ESI+): *m/z*: calcd for $C_{23}H_{20}BrNO$ ([M+H]$^+$) 406.0801, found: 406.0809.

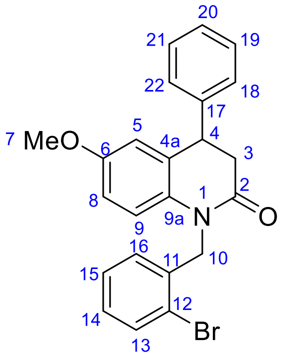

1-(2-Bromobenzyl)-6-methoxy-4-phenyl-3,4-dihydroquinolin-2(1H)-one. **(6c)** was prepared according to the general procedure from *N*-(2-bromobenzyl)-*N*-(4-methoxyphenyl)cinnamamide (**7c**) (422.32 mg, 1mmol) and TFA (4 mL). 198.49 mg (0.47 mmol) of a white solid was obtained with a yield of 47%; R_f = 0.27 (5:1 petroleum ether/ethyl acetate); m.p. = 125-127 °C. **IR** (KBr, ν_{max}/cm-1): 3023 ν(=C-H), 2931 ν(C-H), 1681 ν(C=O), 1296 ν(C-N), 740 ν(C-Br). **^{1}H NMR (**400 MHz, $CDCl_3$) δ(ppm): 7.58 (dd, *J* = 7.6, 1.5 Hz, 1H, 13-H_{Ar}), 7.36 (t, *J*= 7.3 Hz, 2H, 22- and 18-H_{Ar}), 7.32–7.27 (m, 1H, 20-H_{Ar}), 7.21 (d, *J* = 7.0 Hz, 2H, 21- and 19-H_{Ar}), 7.17–7.07 (m, 2H, 14- and 16 -H_{Ar}), 6.85 (dd, *J* = 7.5, 1.7 Hz, 1H, 8-H_{Ar}), 6.73–6.65 (m, 2H, 5- and 15-H_{Ar}), 6.56 (d, *J* = 2.1 Hz, 1H, 9-H_{Ar}), 5.20 (q, *J* = 17.0 Hz, 2H, 10-CH_2), 4.30 (dd, *J* = 7.4, 6.4 Hz, 1H, 4-CH), 3.68 (s, 3H, 7-CH_3), 3.13 (dq, *J* = 15.8, 7.0 Hz, 2H, 3-CH_2). **^{13}C NMR** (101 MHz, $CDCl_3$) δ(ppm): 169.2 (2-CO), 155.7 (6-C_{Ar}), 140.7 (17-C_{Ar}), 135.3 (11-C_{Ar}), 132.9 (13-C_{Ar}), 132.8 (9a-C_{Ar}), 130.7 (16-C_{Ar}), 129.0 (21 and 19-C_{Ar}), 128.7 (14-C_{Ar}), 127.9 (22- and 18-C_{Ar}), 127.8 (20-C_{Ar}), 127.4 (15-C_{Ar}), 127.3 (4a-C_{Ar}), 122.6 (12-C_{Ar}), 116.7 (9-C_{Ar}), 114.8 (8-C_{Ar}), 112.3 (5-C_{Ar}), 55.5 (7-CH_3), 46.7 (10-CH_2), 41.7 (4-CH), 38.8 (3-CH_2). **HRMS** (ESI+): *m/z*: calcd for $C_{23}H_{20}BrNO_2$ ($[M+H]^+$) 422.0750, found: 422.0758.

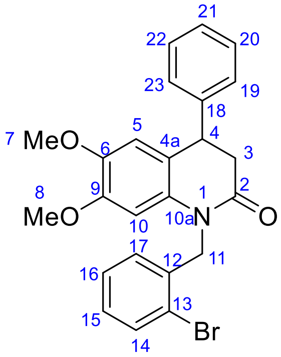

1-(2-Bromobenzyl)-6,7-dimethoxy-4-phenyl-3,4-dihydroquinolin-2(1H)-one. **(6d)** was prepared according to the general procedure from *N*-(2-bromobenzyl)-*N*-(3,4-dimethoxyphenyl)cinnamamide (**7d**) (452.35 mg, 1mmol) and TFA (4 mL). 226.17 mg (0.50 mmol) of a yellow liquid was obtained with a yield of 50%; R_f= 0.23 (5:1 petroleum ether/ethyl acetate). **IR** (KBr, ν_{max}/cm-1): 3025 ν(=C-H), 2931 ν(C-H), 1683 ν(C=O), 1295 ν(C-N), 742 ν(C-Br). **^{1}H NMR (**400 MHz, $CDCl_3$) δ(ppm): 7.59 (dd, *J* = 7.6, 1.5 Hz, 1H, 14-H_{Ar}), 7.39–7.30 (m, 3H, 21, 16 and 15-H_{Ar}), 7.21–7.10 (m, 4H, 23, 22, 20 and 19-H_{Ar}), 6.90 (dd, *J* = 7.4, 1.8 Hz, 1H, 17-H_{Ar}), 6.56 (s, 1H, 10-H_{Ar}), 6.42 (s, 1H, 5-H_{Ar}), 5.30 (q, *J* = 17.0 Hz , 2H, 11-CH_2), 4.28 (t, *J* = 6.5 Hz, 1H, 4-CH), 3.74 (s, 3H, 7-CH_3), 3.74 (s, 3H, 8-CH_3), 3.16 (dd, *J* = 6.4, 2.6 Hz, 2H, 3-CH_2). **^{13}C NMR** (101 MHz, $CDCl_3$) δ(ppm): 169.3 (2-CO), 148.5 (6-C_{Ar}), 144.8 (9-C_{Ar}), 141.3 (18-C_{Ar}), 135.6 (12-C_{Ar}), 132.8 (14- and 17-C_{Ar}), 132.6 (10a-C_{Ar}), 128.9 (22- and 20-C_{Ar}), 128.0 (15-C_{Ar}), 127.9 (21-C_{Ar}), 127.8 (23- and 19-C_{Ar}), 127.3 (16-C_{Ar}), 122.6 (13-C_{Ar}), 120.3 (4a-C_{Ar}), 112.2 (5-C_{Ar}), 101.1 (10-C_{Ar}), 56.2 (7-CH_3), 56.2 (8 -CH_3), 45.8 (11-CH_2), 41.1 (4-CH), 39.3 (3-CH_2). **HRMS** (ESI+): *m/z*: calcd for $C_{24}H_{22}BrNO_3$ ($[M+H]^+$) 452.0855, found: 452.0864.

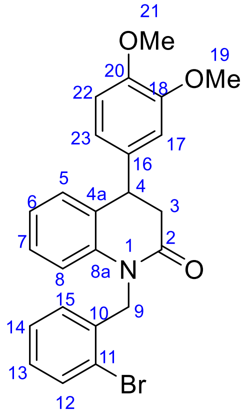

1-(2-Bromobenzyl)-4-(3,4-dimethoxyphenyl)-3,4-dihydroquinolin-2(1H)-one **(6e)** was prepared according to the general procedure from (*E*)-*N*-(2-bromobenzyl)-3-(3,4-dimethoxyphenyl)-*N*-phenyl-acrylamide (**7e**) (452.35 mg, 1mmol) and TFA (4 mL). 248.78 mg (0.55 mmol) of a yellow solid was obtained with a yield of 55%; R_f= 0.30 (5:1 petroleum ether/ethyl acetate); m.p. = 147-150 °C. **IR** (KBr, ν_{max}/cm-1): 3023 ν(=C-H), 2930 ν(C-H), 1684 ν(C=O), 1298 ν(C-N), 742 ν(C-Br). **^{1}H NMR (**400 MHz, $CDCl_3$) δ(ppm): 7.59 (dd, *J* = 7.6, 1.7 Hz, 1H, 12-H_{Ar}), 7.18–7.09 (m, 3H, 15-, 14, and 13-H_{Ar}), 7.01–6.99 (m, 2H, 8- and 5-H_{Ar}), 6.86–6.82 (m, 2H, 7- and 6-H_{Ar}), 6.78–6.74 (m, 2H, 22- and 23-H_{Ar}), 6.73 (s, 1H, 17-H_{Ar}), 5.22 (q, *J* = 17.0 Hz , 2H, 9-CH_2), 4.29 (dd, *J* = 8.3, 5.7 Hz, 1H, 4-CH), 3.89 (s, 3H, 21-CH_3), 3.82 (s, 3H, 19-CH_3), 3.21–3.06 (m, 2H, 3-CH_2). **^{13}C NMR** (101 MHz, $CDCl_3$) δ(ppm): 169.7 (2-CO), 149.3 (20-C_{Ar}), 148.3 (18-C_{Ar}), 139.3 (8a-C_{Ar}), 135.3 (10-C_{Ar}), 133.3 (16-C_{Ar}), 133.0 (12-C_{Ar}), 129.5 (4a-C_{Ar}), 128.7 (15-C_{Ar}), 128.4 (7-C_{Ar}), 128.1 (13-C_{Ar}), 127.7 (14-C_{Ar}), 127.2 (5-C_{Ar}), 123.4 (6-CAr), 122.6 (11-C_{Ar}), 120.1 (17-C_{Ar}), 115.7 (8-C_{Ar}), 111.5 (23-C_{Ar}), 111.1 (22-C_{Ar}), 56.0 (21-CH_3), 56.0 (19-CH_3), 46.7 (9-CH_2), 41.1 (4-CH), 39.0 (3-CH_2). **HRMS** (ESI+): *m/*z: calcd for $C_{24}H_{22}BrNO_3$ ($[M+H]^+$) 452.0855, found: 452.0864.

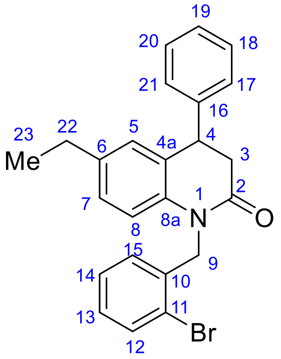

1-(2-Bromobenzyl)-6-ethyl-4-phenyl-3,4-dihydroquinolin-2(1H)-one **(6f)** was prepared according to the general procedure from *N*-(2-bromobenzyl)-*N*-(4-ethylphenyl)cinnamamide (**7f**) (420.35 mg, 1 mmol) and TFA (4 mL). 180.75 mg (0.68 mmol) of a white solid was obtained with a yield of 68%; R_f = 0.43 (5:1 petroleum ether/ethyl acetate); m.p. = 111-113 °C. **IR** (KBr, ν_{max}/cm^{-1}): 3023 ν(=C-H), 2962 ν(C-H), 1666 ν(C=O), 1373 ν(C-N), 740 ν(C-Br). **^{1}H NMR** (400 MHz, $CDCl_3$) δ(ppm): 7.59 (dd, *J* = 7.6, 1.6 Hz, 1H, 12-H_{Ar}), 7.39–7.33 (m, 2H, 21- and 17-H_{Ar}), 7.32–7.27 (m, 1H, 19-H_{Ar}), 7.23–7.18 (m, 2H, 20- and 18-H_{Ar}), 7.12 (pd, *J* = 7.3, 1.7 Hz, 2H, 13- and 15-H_{Ar}), 7.00 (dd, *J* = 8.3, 1.9 Hz, 1H, 7-H_{Ar}), 6.85 (dd, *J* = 7.1, 1.6 Hz, 2H, 5- and 14-H_{Ar}), 6.70 (d, *J* = 8.3 Hz, 1H, 8-H_{Ar}), 5.27–5.13 (m, 2H, 9-CH_2), 4.32 (t, *J* = 6.6 Hz, 1H, 4-CH), 3.22–3.06 (m, 2H, 3-CH_2), 2.53 (q, *J* = 7.6 Hz, 2H, 22-CH_2), 1.15 (t, *J* = 7.6 Hz, 3H, 23-CH_3). **^{13}C NMR** (101 MHz, $CDCl_3$) δ(ppm): 169.4 (2-CO), 141.1 (16-C_{Ar}), 139.5 (6-C_{Ar}), 137.1 (8a-C_{Ar}), 135.4 (10-C_{Ar}), 132.9 (12-C_{Ar}), 128.9 (20- and 18-C_{Ar}), 128.8 (4a-C_{Ar}), 128.6 (15-C_{Ar}), 128.1 (13-C_{Ar}), 127.9 (21- and 17-C_{Ar}), 127.7 (5-C_{Ar}), 127.4 (14-C_{Ar}), 127.3 (7-C_{Ar}), 127.2 (19-C_{Ar}), 122.6 (11-C_{Ar}), 115.7 (8-C_{Ar}), 46.7 (9-CH_2), 41.6 (4-CH), 39.0 (3-CH_2), 28.1 (22-CH_2), 15.6 (23-CH_3). **DEPT 135** (101 MHz, $CDCl_3$) δ(ppm): 132.9 (12-C_{Ar}), 128.9 (20- and 18-C_{Ar}), 128.6 (15-C_{Ar}), 128.1 (13-C_{Ar}), 127.8 (21- and 17-C_{Ar}), 127.7 (5-C_{Ar}), 127.3 (14-C_{Ar}), 127.3 (7-C_{Ar}), 127.2 (19-C_{Ar}), 115.7 (8-C_{Ar}), 46.7 (9-CH_2), 41.5 (4-CH), 39.0 (3-CH_2), 28.1 (22-CH_2), 15.6 (23-CH_3). **COSY** Correlation [δH/δH]: 1.15/2.53 [23-CH_3/22-CH_2], 3.22–3.06/4.32 [3-CH_2/4-CH], 6.70/7.00 [8-H_{Ar}/15-

H_{Ar}], 6.85/7.12 [14-H_{Ar}/13- and 15-H_{Ar}], 7.12/7.59 [13- and 15-H_{Ar}/12-H_{Ar}],7.21/7.36 [20- and 18-H_{Ar}/21 and 17-H_{Ar}]. **HSQC** Correlation [δH/δC]: 1.15/15.6 [23-CH_3/23-CH_3], 2.53/28.1 [22-CH_2/22-CH_2], 3.22–3.06/39.0 [3-CH_2/3-CH_2], 4.32/41.6 [4-CH /4-CH], 5.27 – 5.13/46.7 [9-CH_2/9-CH_2], 6.70/115.7 [8-H_{Ar}/8-C_{Ar}], 6.85/127.4 [5- and 14-H_{Ar}/5- and/or 14-C_{Ar}], 7.00/127.3 [7-H_{Ar}/7-C_{Ar}], 7.12/128.6 [15-H_{Ar}/15-C_{Ar}], 7.23–7.18/128.9 [20- and 18-H_{Ar}/20 and 18-C_{Ar}], 7.32–7.27/127.2 [19-H_{Ar}/19-C_{Ar}], 7.39–7.33/127.9 [21- and 17-H_{Ar}/21 and 17-C_{Ar}], 7.59/132.9 [12-H_{Ar}/12-C_{Ar}]. **HMBC** Correlation [δH/δC]: 1.15/28.1/139.5 [23-CH_3/22-CH_2/6-C_{Ar}], 2.53/15.6/127.7/139.5 [22-CH_2/23-CH_3/5-C_{Ar}/6-C_{Ar}], 3.22–3.06/41.6/ 128.8/141.1/169.4 [3-CH_2/4-CH/4a-C_{Ar}/16-C_{Ar}/2-CO], 4.32/39.0/128.8/ 137.1/141.1/169.4 [4-CH/3-CH_2/4a-C_{Ar}/8a-C_{Ar}/16-C_{Ar}/2-CO], 5.27 – 5.13/122.6/135.4/169.4 [9-CH_2/11-C_{Ar}/10-C_{Ar}/2-CO], 6.7/128.8/139.5 [8-H_{Ar}/4a-C_{Ar}/6-C_{Ar}], 6.8/28.1/41.6/128.1/135.4 [5- and 14-H_{Ar}/22-CH_2/4-CH/13-C_{Ar}/10-C_{Ar}], 7.00/28.1/115.7/127.7/137.1 [7-H_{Ar}/22-CH_2/8-C_{Ar}/5-C_{Ar}/8a-C_{Ar}], 7.12/122.6/ 127.4/132.9/135.4 [13 and 15-H_{Ar}/11-C_{Ar}/14-C_{Ar}/12-C_{Ar}/10-C_{Ar}]. 7.23-7.18/41.6/127.2 [20- and 18-H_{Ar}/4-CH/19-C_{Ar}], 7.32–7.27/127.9 [19-H_{Ar}/21 and 17-C_{Ar}], 7.39–7.33/128.9/141.1 [21- and 17-H_{Ar}/20- and 18-C_{Ar}/16-C_{Ar}], 7.59/127.4/135.4/122.6 [12-H_{Ar}/14-C_{Ar}/10-C_{Ar}/11-C_{Ar}]. **HRMS** (ESI+): *m/z*: calcd for $C_{24}H_{22}BrNO$ ($[M+H]^+$) 420.0957, found: 420965.

6. Characterization data of all synthesized pyrido[3,2,1-*de*]phenanthridin-6-ones 4a-f

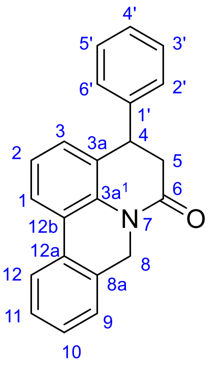

***4-Phenyl-4,5-dihydro-6H,8H-pyrido[3,2,1-de]phenanthridin-6-one* (4a)** was prepared according to the general procedure from 1-(2-bromobenzyl)-4-phenyl-3,4-dihydroquinolin-2(1*H*)-one **6a** (392.29 mg, 1 mmol). 289.58 mg (0.93 mmol) of a yellow solid was obtained with a yield 93%; R_f = 0.50 (4:1 petroleum ether/ethyl acetate); m.p. = 159–160 °C. **IR** (KBr, ν_{max}/cm^{-1}): 3023 ν(=C-H), 2854 ν(C-H), 1681 ν(C=O), 1388 ν(C-N). **^{1}H NMR** (400 MHz, $CDCl_3$) $\delta_{(ppm)}$: 7.76 (t, *J* = 7.0 Hz, 2H, 1- and 12-H_{Ar}), 7.38-7.36 (m, 2H, 2’- and 6’-H_{Ar}), 7.34 (s, 1H, 11-H_{Ar}), 7.31-7.29 (m, 3H, 4’-, 9- and 10-H_{Ar}), 7.23–7.17 (m, 2H, 3’- and 5’-H_{Ar}), 7.09 (t, *J* = 7.7 Hz, 1H, 2-H_{Ar}), 6.89 (d, *J* = 7.5 Hz, 1H, 3-H_{Ar}), 5.11 (d, *J* = 15.6 Hz, 1H, 8-CH_2), 5.00 (d, *J* = 15.6 Hz, 1H, 8-CH_2), 4.28 (t, *J* = 7.3 Hz, 1H, 4-CH), 3.03–2.91 (m, 2H, 5-CH_2). **^{13}C NMR** (101 MHz, $CDCl_3$) $\delta_{(ppm)}$: 168.3 (6-CO), 141.0 (1’-C_{Ar}), 135.4 ($3a^1$-C_{Ar}), 131.5 (12a-C_{Ar}), 130.6 (8a-C_{Ar}), 129.8 (3a-C_{Ar}), 129.0 (3-’ and 5’-C_{Ar}), 128.3 (10-C_{Ar}) 128.2 (11-C_{Ar}), 127.9 (3-C_{Ar}), 127.8 (2’- and 6’-C_{Ar}), 127.4 (9-C_{Ar}), 126.7 (4’-C_{Ar}), 124.1 (12b-C_{Ar}), 124.0 (2-C_{Ar}), 123.2 (1-C_{Ar}), 122.6 (12-C_{Ar}), 42.6 (8-CH_2), 41.6 (4-CH), 38.7 (5-CH_2). **HRMS** (ESI+): *m/z*: calcd for $C_{22}H_{17}NO$ ($[M+H]^+$) 312.1383, found: 312.1389.

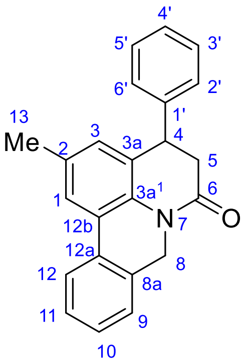

2-Methyl-4-phenyl-4,5-dihydro-6H,8H-pyrido[3,2,1-de]phenanthridin-6-one **(4b)** was prepared according to the general procedure from 1-(2-bromobenzyl)-6-methyl-4-phenyl-3,4-dihydro-quinolin-2(1*H*)-one (**6b**) (406.32 mg, 1 mmol). 302.63 mg (0.93 mmol) of a yellow solid was obtained with a yield of 93%; R_f = 0.50 (4:1 petroleum ether/ethyl acetate); m.p. = 178–181 °C. **IR** (KBr, ν_{max}/cm^{-1}): 3023 ν(=C-H), 2854 ν(C-H), 1666 ν(C=O), 1373 ν(C-N). **^{1}H NMR** (400 MHz, $CDCl_3$) $\delta_{(ppm)}$: 7.77 (d, *J* = 7.6 Hz, 1H, 12-H_{Ar}), 7.56 (s, 1H, 1-H_{Ar}), 7.40–7.32 (m, 3H, 2'-, 6'- and 11-H_{Ar}), 7.30-7.28 (m, 3H, 4'-, 9- and 10-H_{Ar}), 7.23–7.17 (m, 2H, 3'- and 5'-H_{Ar}), 6.72 (d, *J* = 0.8 Hz, 1H, 3-H_{Ar}), 5.04 (s, 2H, 8-CH_2), 4.24 (t, *J* = 7.0 Hz, 1H, 4-CH), 2.94 (d, *J* = 7.1 Hz, 2H, 5-CH_2), 2.32 (s, 3H, 13-CH_3). **^{13}C NMR** (101 MHz, $CDCl_3$) $\delta_{(ppm)}$: 168.1 (6-CO), 141.2 (1'-C_{Ar}), 133.5 (12a-C_{Ar}), 133.1 (3a^1-C_{Ar}), 131.5 (8a-C_{Ar}), 130.6 (2-C_{Ar}), 129.5 (3a-C_{Ar}), 129.0 (3-' and 5'-C_{Ar}), 128.6 (9-C_{Ar}), 128.2 (11-C_{Ar}), 128.1 (10-C_{Ar}), 127.8 (2' and 6'-C_{Ar}), 127.3 (3-C_{Ar}), 126.7 (4'-C_{Ar}), 123.9 (12b-C_{Ar}), 123.2 (1-C_{Ar}), 123.0 (12-C_{Ar}), 42.7 (8-CH_2), 41.6 (4-CH), 38.9 (5-CH_2), 21.2 (13-CH_3). **HRMS** (ESI+): *m/z*: calcd for $C_{23}H_{19}NO$ ($[M+H]^+$) 326.1539, found: 326.1545.

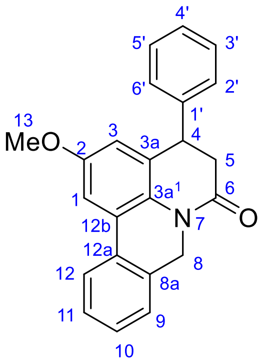

2-Methoxy-4-phenyl-4,5-dihydro-6H,8H-pyrido[3,2,1-de]phenanthridin-6-one **(4c)** was prepared according to the general procedure from 1-(2-bromobenzyl)-6-methoxy-4-phenyl-3,4-dihydroquinolin-2(1*H*)-one (**6c**) (422.32 mg, 1 mmol). 337.99 mg (0.9 mmol) of a yellow solid was obtained with a yield of 99%; R_f = 0.34 (4:1 petroleum ether/ethyl acetate); m.p. = 133–135 °C. **IR** (KBr, ν_{max}/cm^{-1}): 3055 ν(=C-H), 2839 ν(C-H), 1666 ν(C=O), 1388 ν(C-N). **^{1}H NMR** (400 MHz, $CDCl_3$) $\delta_{(ppm)}$: 7.73 (d, *J* = 7.6 Hz, 1H, 12-H_{Ar}), 7.42-7.25 (m, 7H, 2'-, 4'-, 6'-, 1-, 9-, 10- and 11-H_{Ar}), 7.22–7.18 (m, 2H, 3'- and 5'-H_{Ar}), 6.47 (d, *J* = 2.7 Hz, 1H, 3-H_{Ar}), 5.07 (d, *J* = 15.7 Hz, 1H, 8-CH_2), 4.98 (d, *J* = 15.6 Hz, 1H, 8-CH_2), 4.24 (t, *J* = 7.1 Hz, 1H, 4-CH), 3.78 (s, 3H, 13-CH_3), 2.94 (d, *J* = 7.2 Hz, 2H, 5-CH). **^{13}C NMR** (101 MHz, $CDCl_3$) $\delta_{(ppm)}$: 167.9 (6-CO), 156.2 (2-C_{Ar}), 140.8 (1'-C_{Ar}), 131.8 (12a-C_{Ar}), 131.3 (8a-C_{Ar}), 130.5 (3a^1-C_{Ar}), 129.16(3a-C_{Ar}), 129.0 (3'- and 5'-C_{Ar}), 128.4 (11-C_{Ar}), 128.2 (10-C_{Ar}), 127.8 (2-' and 6'-C_{Ar}), 127.4 (9-C_{Ar}), 126.7 (4'-C_{Ar}), 125.2 (12b-C_{Ar}), 123.3 (12-C_{Ar}), 114.1 (3-C_{Ar}), 107.2 (1-C_{Ar}), 55.6 (13-CH_3), 42.7 (8-CH_2), 41.8 (4-CH), 38.8 (5-CH_2). **HRMS** (ESI+): *m/z*: calcd for $C_{23}H_{19}NO_2$ ($[M+H]^+$) 342.1488, found: 342.1495.

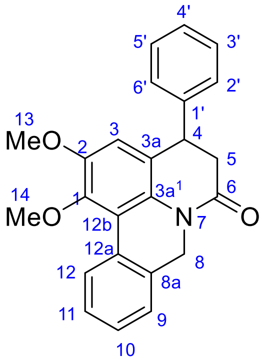

1,2-Dimethoxy-4-phenyl-4,5-dihydro-6H,8H-pyrido[3,2,1-de]phenanthridin-6-one **(4d)** was prepared according to the general procedure from 1-(2-bromobenzyl)-6,7-dimethoxy-4-phenyl-3,4-dihydroquinolin-2(1*H*)-one (**6d**) (452.35 mg, 1 mmol). 352.86 mg (0.95 mmol) of a yellow liquid was obtained with a yield of 95%; R_f = 0.30 (4:1 petroleum ether/ethyl acetate). **IR** (KBr, ν_{max}/cm^{-1}): 3054 ν(=C-H), 2838 ν(C-H), 1662 ν(C=O), 1387 ν(C-N). **^{1}H NMR** (400 MHz, $CDCl_3$) $\delta_{(ppm)}$: 8.49 (d, *J* = 8.1 Hz, 1H, 12-H_{Ar}), 7.38–7.33 (m, 3H, 9, 10 and 11-H_{Ar}), 7.32–7.28 (m, 3H, 2', 4' and 6'-H_{Ar}), 7.21–7.17 (m, 2H, 3'- and 5'-H_{Ar}), 6.49 (s, 1H, 3-H_{Ar}), 4.92 (s, 2H, 8-CH_2), 4.21 (t, *J* = 7.0 Hz, 1H, 4-CH), 3.81 (s, 3H, 13-CH_3), 3.75 (s, 3H, 14-CH_3), 2.92–2.89 (m, 2H, 5-CH_2).**^{13}C NMR** (101 MHz, $CDCl_3$) $\delta_{(ppm)}$: 167.6 (6-CO), 149.5 (2-C_{Ar}), 146.6 (1-C_{Ar}), 141.1 (1'-C_{Ar}), 132.5 (12a-C_{Ar}), 130.5 ($3a^1$-C_{Ar}), 129.6 (3a-C_{Ar}), 129.1 (3'- and 5'-C_{Ar}), 128.2 (11-C_{Ar}), 128.1 (10-C_{Ar}), 127.8 (2'- and 6'-C_{Ar}), 127.5 (12-C_{Ar}), 127.4 (9-C_{Ar}), 126.4 (4'-C_{Ar}), 125.1 (12b-C_{Ar}), 119.2 (8a-C_{Ar}), 112.1 (3-C_{Ar}), 60.7 (13-CH_3), 56.4 (14-CH_3), 42.8 (8-CH_2), 41.9 (4-CH), 39.1 (5-CH_2). **HRMS** (ESI+): *m/z*: calcd for $C_{24}H_{21}NO_3$ ($[M+H]^+$) 372.1594, found: 372.1605

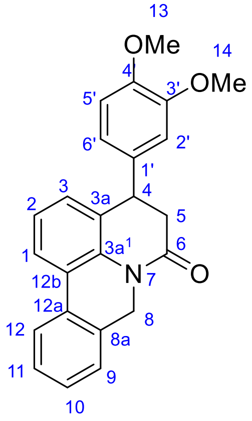

4-(3,4-Dimethoxyphenyl)-4,5-dihydro-6H,8H-pyrido[3,2,1-de]phenanthridin-6-one **(4e)** was prepared according to the general procedure from 1-(2-bromobenzyl)-4-(3,4-dimethoxyphenyl)-3,4-dihydroquinolin-2(1*H*)-one (**6e**) (452.35 mg, 1 mmol). 334.3 mg (0.90 mmol) of a yellow liquid was obtained with a yield of 90%; R_f = 0.26 (4:1 petroleum ether/ethyl acetate). **IR** (KBr, ν_{max}/cm^{-1}): 3053 ν(=C-H), 2837 ν(C-H), 1665 ν(C=O), 1388 ν(C-N). **^{1}H NMR** (400 MHz, $CDCl_3$) $\delta_{(ppm)}$: 7.75 (t, *J* = 7.6 Hz, 2H, 6'- and 5'-H_{Ar}), 7.40-7.35 (m, 1H, 12-H_{Ar}), 7.33–7.28 (m, 2H, 3- and 1-H_{Ar}), 7.09 (t, *J* = 7.7 Hz, 1H, 2-H_{Ar}), 6.91 (dt, *J* = 7.5, 1.2 Hz, 1H, 9-H_{Ar}), 6.83 (d, *J* = 8.0 Hz, 1H, 11-H_{Ar}), 6.75–6.71 (m, 2H, 10- and 2'-H_{Ar}), 5.04 (q, 1H, 8-CH_2), 4.22 (t, *J* = 7.4 Hz, 1H, 4-CH), 3.87 (s, 3H, 13-CH_3), 3.82 (s, 3H, 14-CH_3), 2.94 (d, *J*= 7.9 Hz, 1H, 5-CH_2). **^{13}C NMR** (101 MHz, $CDCl_3$) $\delta_{(ppm)}$: 168.4 (6-CO), 149.3 (4'-C_{Ar}), 148.3 (3'-C_{Ar}), 135.3 (1'-C_{Ar}), 133.4 ($3a^1$-C_{Ar}), 131.5 (12a-C_{Ar}), 130.6 (8a-C_{Ar}), 130.1 (3a-C_{Ar}), 128.3 (12- and 9-C_{Ar}), 127.8 (11-C_{Ar}), 126.7 (10-C_{Ar}), 124.1 (12b-C_{Ar}), 124.0 (3-C_{Ar}), 123.2 (1-C_{Ar}), 122.6 (2-C_{Ar}), 120.0 (5'-C_{Ar}), 111.6 (6'-C_{Ar}), 110.9 (2'-C_{Ar}), 56.0 (13-CH_3), 56.0 (14-CH_3), 42.69 (8-CH_2), 41.3 (4-CH), 38.9 (5-CH_2). **HRMS** (ESI+): *m/z*: calcd for $C_{24}H_{21}NO_3$ ($[M+H]^+$) 372.1594, found: 372.1601.

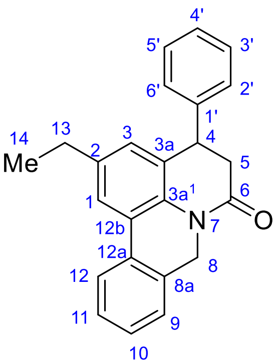

2-Ethyl-4-phenyl-4,5-dihydro-6H,8H-pyrido[3,2,1-de]phenanthridin-6-one **(4f)** was prepared according to the general procedure from 1-(2-bromobenzyl)-6-ethyl-4-phenyl-3,4-dihydro-quinolin-2(1*H*)-one **6f** (420.35 mg, 1 mmol). 315.67 mg (0.93 mmol) of a yellow solid was obtained with a yield of 93%; R_f = 0.50 (4:1 petroleum ether/ethyl acetate); m.p. = 143–146 °C. **IR** (KBr, ν_{max}/cm^{-1}): 3023.89 ν(=C-H), 2962.18 ν(C-H), 1666.23 ν(C=O), 1373.09 ν(C-N). **^{1}H NMR** (400 MHz, $CDCl_3$) $\delta_{(ppm)}$: 7.79 (d, *J* = 7.6 Hz, 1H, 12-H_{Ar}), 7.59 (d, *J* = 1.5 Hz, 1H, 1-H_{Ar}), 7.41–7.33 (m, 3H, 3'-, 5-' and 11-H_{Ar}), 7.33–7.26 (m, 3H, 4'-, 9- and 10-H_{Ar}), 7.23–7.17 (m, 2H, 2'- and 6'-H_{Ar}), 6.75 (d, *J* = 0.9 Hz, 1H, 3-H_{Ar}), 5.04 (s, 2H, 8-CH_2), 4.26 (t, *J* = 7.0 Hz, 1H, 4-CH), 2.95 (d, *J* = 7.0 Hz, 2H, 5-CH_2), 2.61 (q, *J* = 7.6 Hz, 2H, 13-CH_2), 1.22 (t, *J* = 7.6 Hz, 3H, 14-CH_3). **^{13}C NMR** (101 MHz, $CDCl_3$) $\delta_{(ppm)}$: 168.1 (6-CO), 141.2 (1'-C_{Ar}), 140.0 (2-C_{Ar}), 133.2 (3a^1-C_{Ar}), 131.5 (12a-C_{Ar}), 130.7(8a-C_{Ar}), 129.6 (3a-C_{Ar}), 129.0 (3' and 5'-C_{Ar}), 128.1 (11-C_{Ar}), 128.1 (10-C_{Ar}), 127.8 (2'- and 6'-C_{Ar}), 127.5 (9-C_{Ar}), 127.3 (3-C_{Ar}), 126.7 (4'-C_{Ar}), 124.0 (12b-C_{Ar}), 123.2 (12-C_{Ar}), 121.8 (1-C_{Ar}), 42.6 (8-CH_2), 41.7 (4-CH), 38.9 (5-CH_2), 28.6 (13-CH_2), 15.8 (14-CH_3). **DEPT 135** (101 MHz, $CDCl_3$) $\delta_{(ppm)}$: 129.0 (3'- and 5'-C_{Ar}), 128.1 (11-C_{Ar}), 128.1 (10-C_{Ar}) 127.8 (2'- and 6'-C_{Ar}), 127.5 (9-C_{Ar}), 127.3 (3-C_{Ar}), 126.7 (4'-C_{Ar}), 123.2 (12-C_{Ar}), 121.8 (1-C_{Ar}), 42.6 (8-CH_2), 41.7 (4-CH), 38.9 (5-CH_2), 28.6 (13-CH_2), 15.8 (14-CH_3). **COSY** [δH/δH]: 1.22/2.61 [14-CH_3/13-CH_2], 2.95/4.26 [5-CH_2/4-CH], 6.75/7.59 [3-H_{Ar}/1-H_{Ar}], 7.23–7.17/7.41–7.33 [3'- and 5'-H_{Ar}/2' and 6'-H_{Ar}], 7.41–7.33/7.79 [11-H_{Ar}/12-H_{Ar}]. **HSQC** [δH/δC]: 1.22/15.8 [14-CH_3/14-CH_3], 2.61/28.6 [13-CH_2/13-CH_2], 2.95/38.9 [5-CH_2/5-CH_2], 4.26/41.7 [4-CH/4-CH], 5.04/42.6 [8-CH_2/8-CH_2], 6.75/127.3 [3-H_{Ar}/3-C_{Ar}], 7.23 – 7.17/127.8 [2'- and 6'-H_{Ar}/2'- and 6'-C_{Ar}], 7.33 – 7.26/126.7 [4'-H_{Ar}/4'-C_{Ar}], 7.41–7.33/128.1/ 129.0 [3'-, 5'- and 11-H_{Ar}/11'-C_{Ar}/3'- and 5'-C_{Ar}], 7.59/121.8 [1-H_{Ar}/1-C_{Ar}], 7.79/123.2 [12-H_{Ar}/12-C_{Ar}]. **HMBC** [δH/δC]: 1.22/28.63/140.0 [14-CH_3/13-CH_2/2-C_{Ar}], 2.61/15.8/121.8/127.3/ 140.0 [13-CH_2/14-CH_3/1-C_{Ar}/3-C_{Ar}/2-C_{Ar}], 2.95/ 41.7/129.6/141.2/168.1 [5-CH_2/4-CH/3a-C_{Ar}/1'-C_{Ar}/6-CO], 4.26/38.9/ 129.0/133.2/141.2/ 168.1 [4-CH/5-CH_2/2' and 6'-C_{Ar}/3a^1-C_{Ar}/1'-C_{Ar}/6-CO], 5.04/127.5/130.7/133.2/168.1[8-CH_2/9-C_{Ar}/8a-C_{Ar}/3a^1-C_{Ar}/6-CO], 6.75/28.6/41.7/121.8133.2 [3-H_{Ar}/13-CH_2/4-CH/1-C_{Ar}/3a^1-C_{Ar}], 7.23–7.17/41.7/127.3 [2'- and 6'-H_{Ar}/4-CH/3-C_{Ar}], 7.33–7.26/41.7/123.2/128.1/ 130.7/141.2 [4-', 9- and 10-H_{Ar}/4-CH/12-C_{Ar}/11-C_{Ar}/8a-C_{Ar}/1'-C_{Ar}], 7.41–7.33/127.8/141.2 [3', 5' and 11-H_{Ar}/2'- and 6'-C_{Ar}/1'-C_{Ar}], 7.59/28.6/127.3/131.5/133.2 [1-H_{Ar}/13-CH_2/12a-C_{Ar}/3a^1-C_{Ar}], 7.79/124.0/ 128.1/131.5 [12-H_{Ar}/12b-C_{Ar}/11-C_{Ar}/12a-C_{Ar}]. **HRMS** (ESI+): *m/z*: calcd for $C_{24}H_{21}NO$ ($[M+H]^+$) 340.1695, found: 340.1702.

7. Figures S1-S16 of the respective ^{1}H and ^{13}C NMR spectra of all synthesized *N*-arylcinnamamides 8a-f

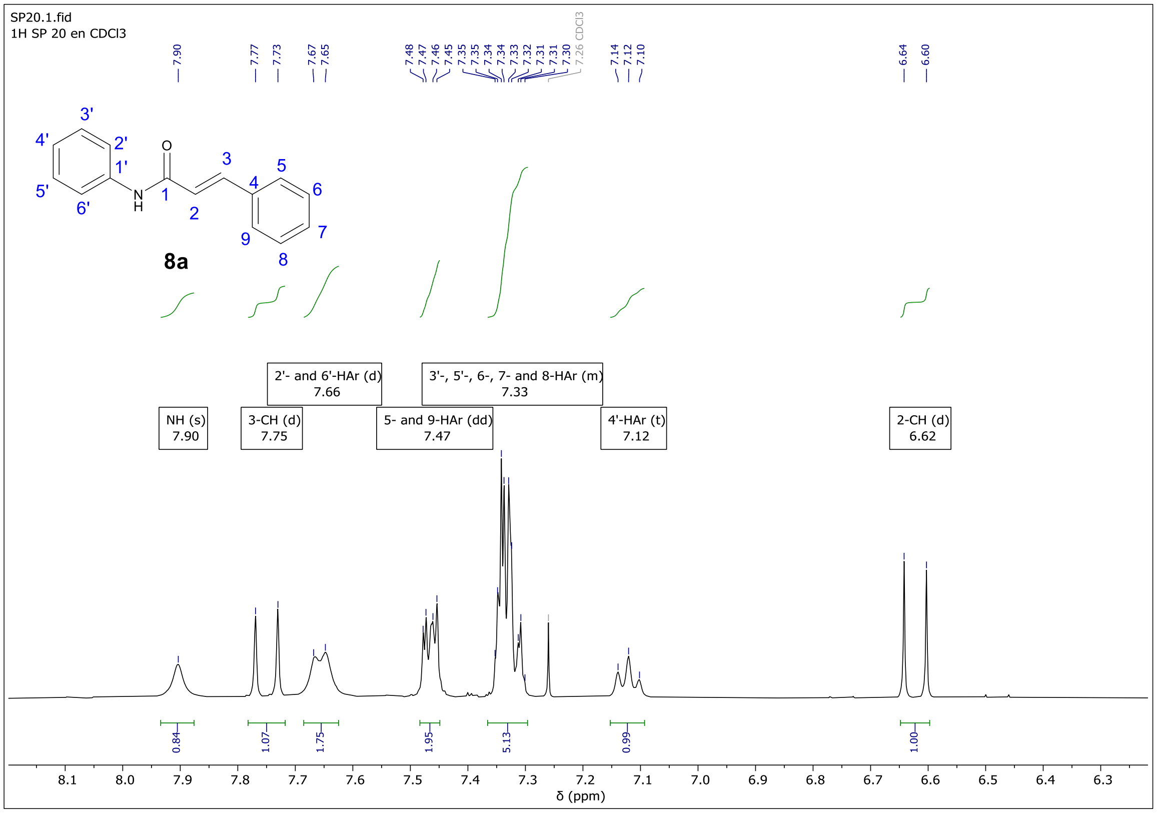

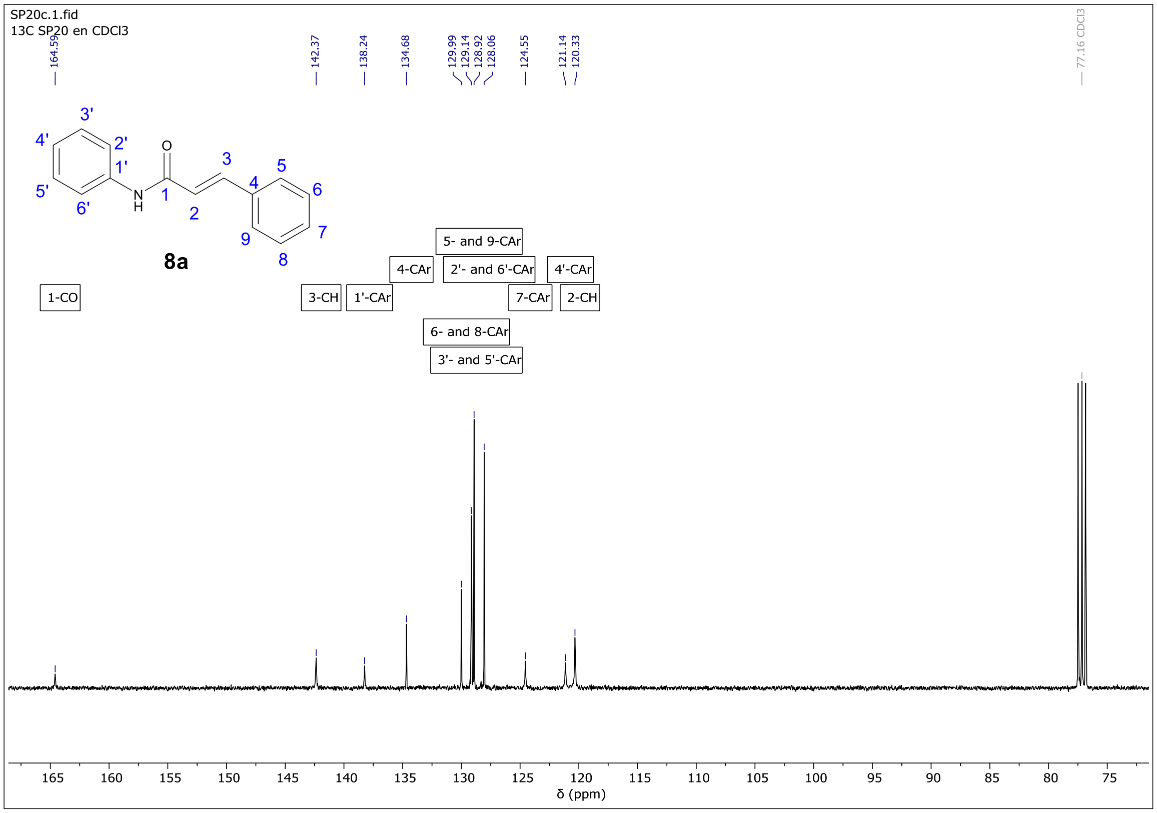

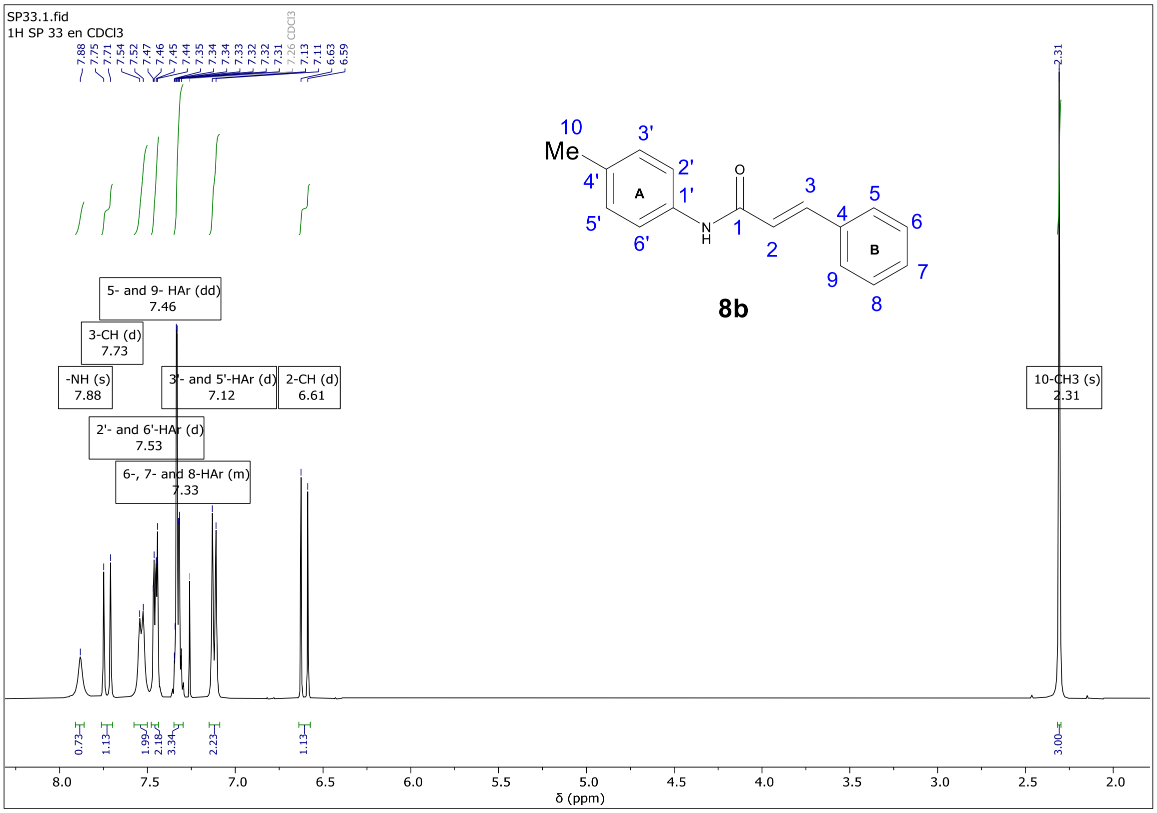
SP33.1.fid
1H SP 33 en CDCl3
8b
5- and 9- HAr (dd)
7.46
3-CH (d)
7.73
-NH (s)
7.88
3'- and 5'-HAr (d)
7.12
2-CH (d)
6.61
10-CH3 (s)
2.31
2'- and 6'-HAr (d)
7.53
6-, 7- and 8-HAr (m)
7.33
δ (ppm)

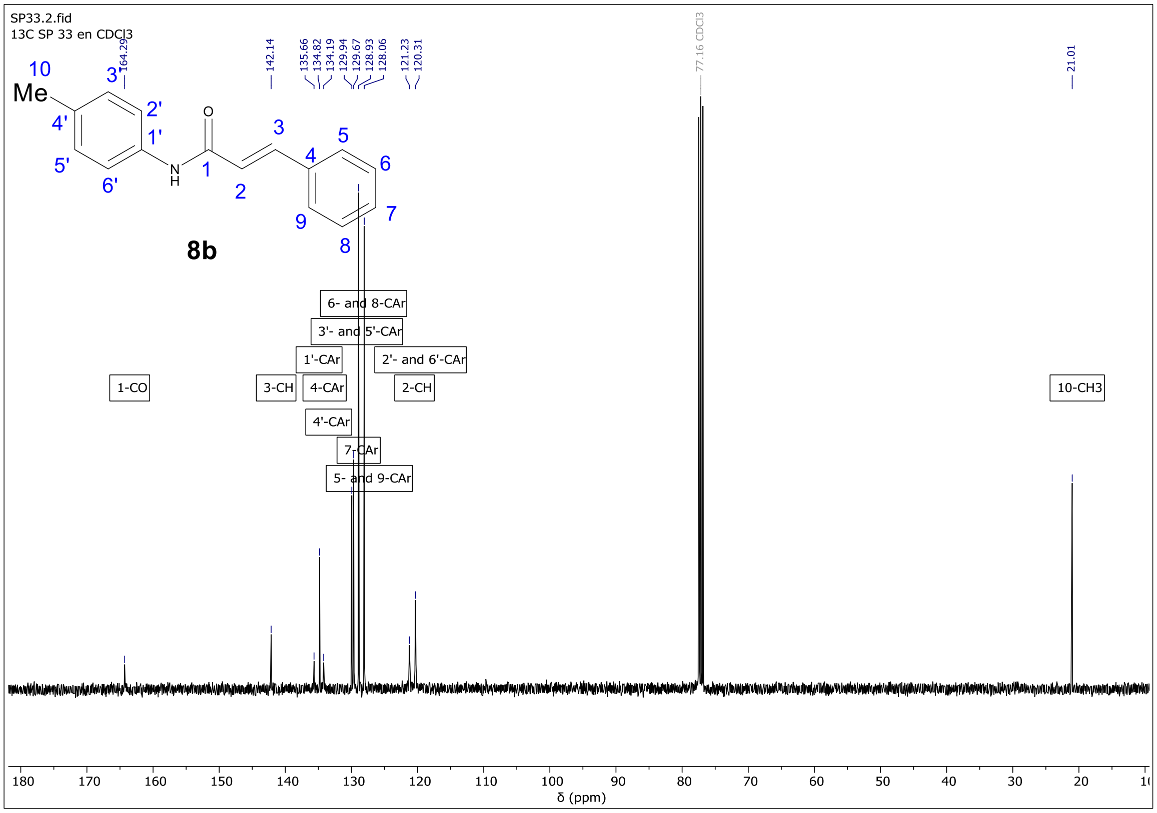
SP33.2.fid
13C SP 33 en CDCl3
8b
6- and 8-CAr
3'- and 5'-CAr
1'-CAr
2'- and 6'-CAr
1-CO
3-CH
4-CAr
2-CH
10-CH3
4'-CAr
7-CAr
5- and 9-CAr
δ (ppm)

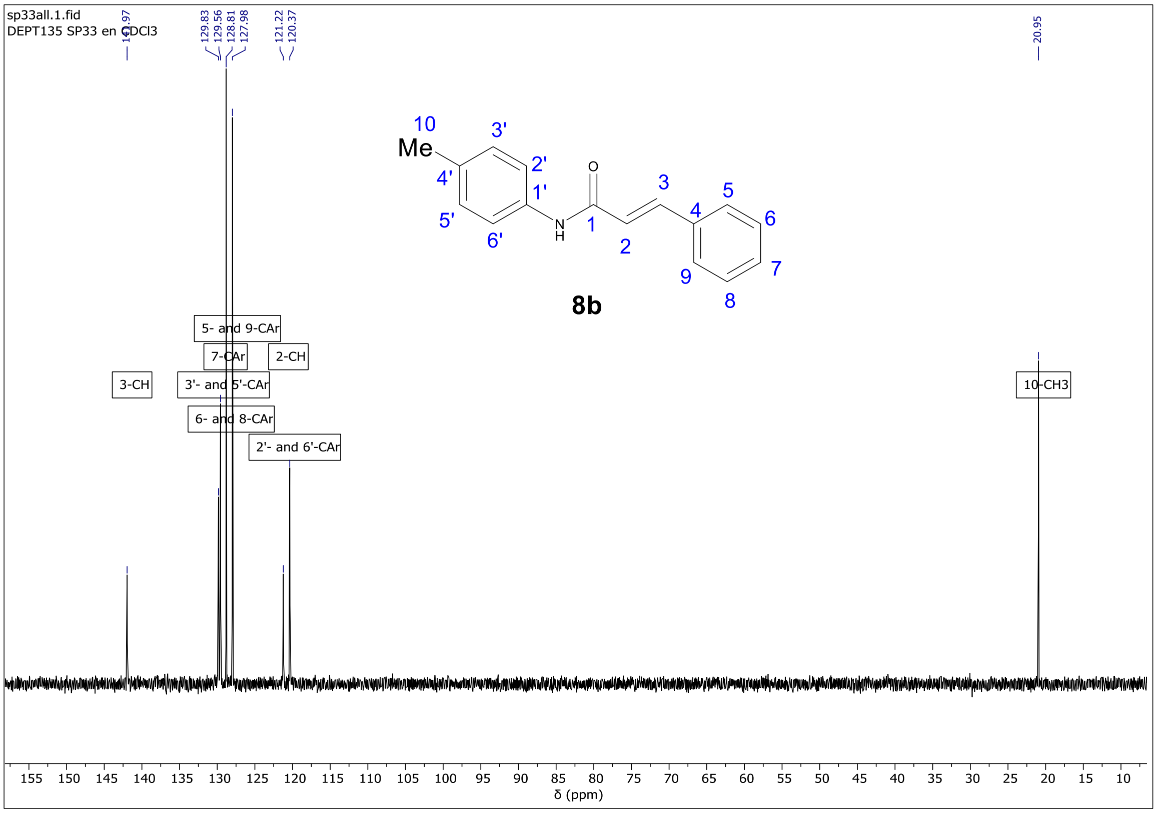
sp33all.1.fid
DEPT135 SP33 en CDCl3
129.83
129.56
128.81
127.98
121.22
120.37
20.95
8b
5- and 9-CAr
7-CAr
2-CH
3-CH
3'- and 5'-CAr
6- and 8-CAr
2'- and 6'-CAr
10-CH3
δ (ppm)

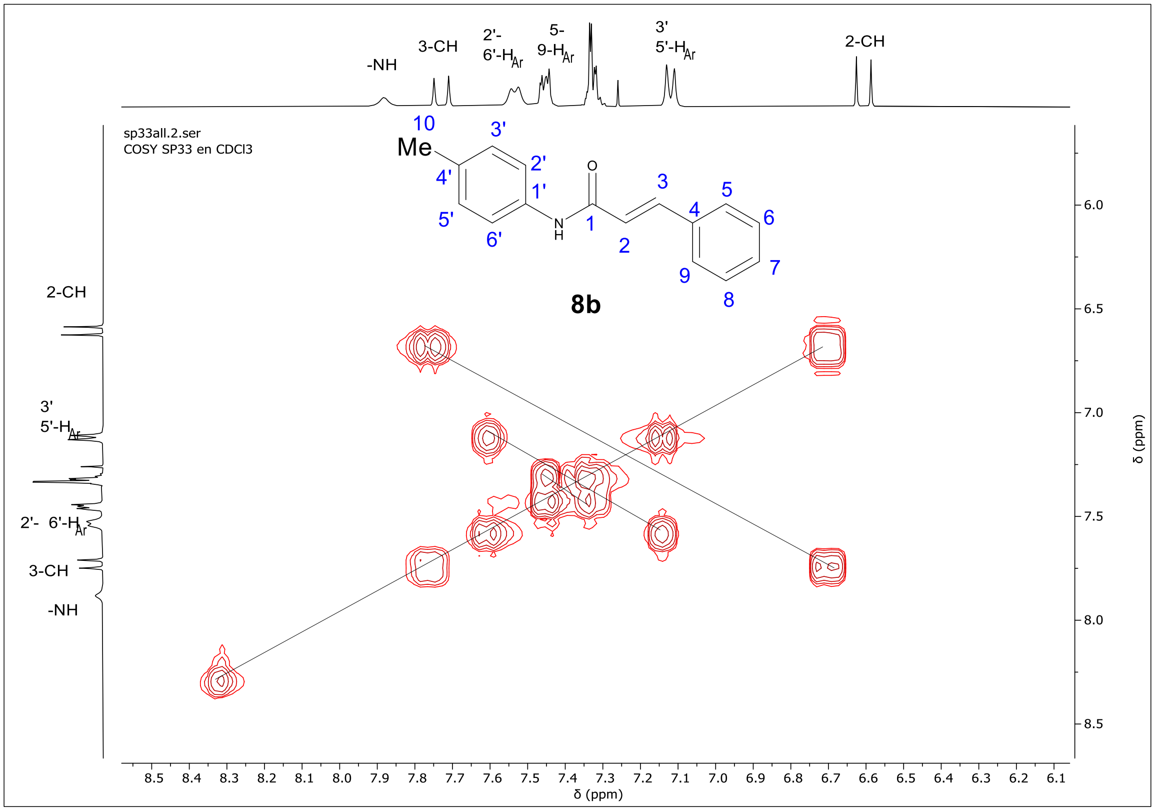
sp33all.2.ser
COSY SP33 en CDCl3
-NH
3-CH
2'- 6'-HAr
5- 9-HAr
3' 5'-HAr
2-CH
8b
δ (ppm)

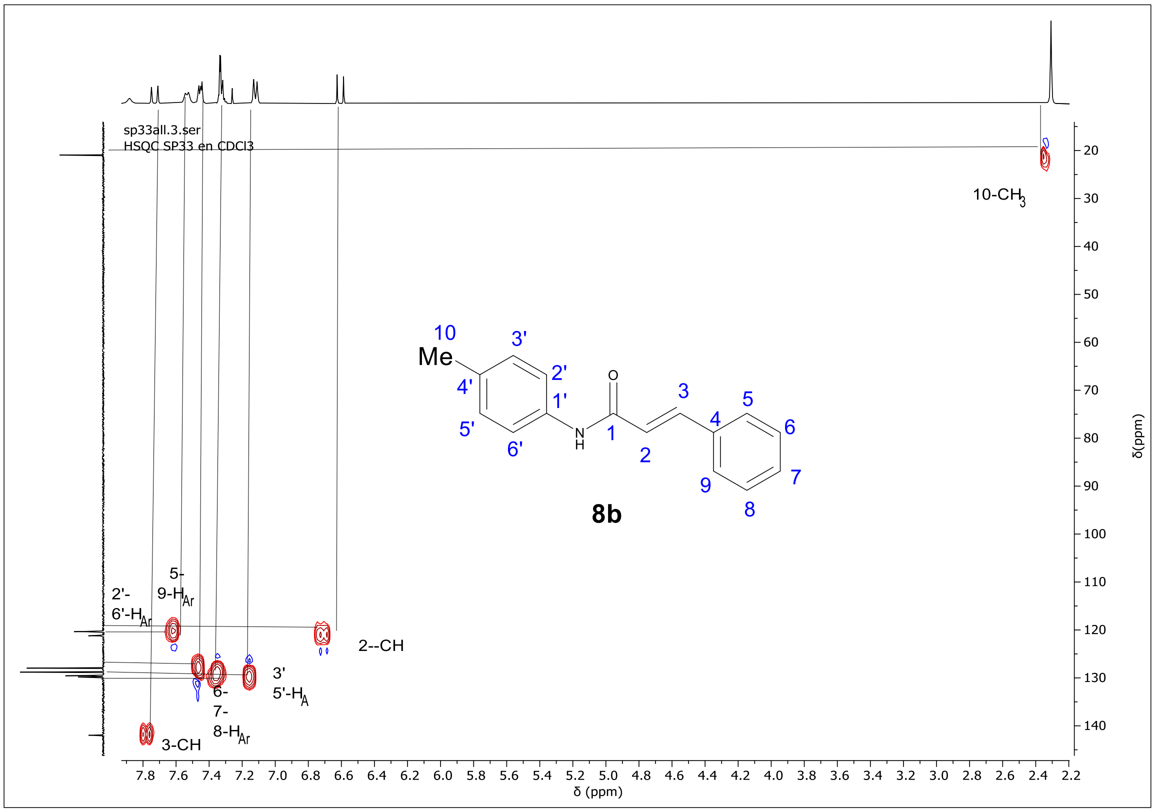
sp33all.3.ser
HSQC SP33 en CDCl3
10-CH3
2'-6'-HAr
5-9-HAr
2--CH
3'5'-HA
6-7-8-HAr
3-CH
8b
δ (ppm)

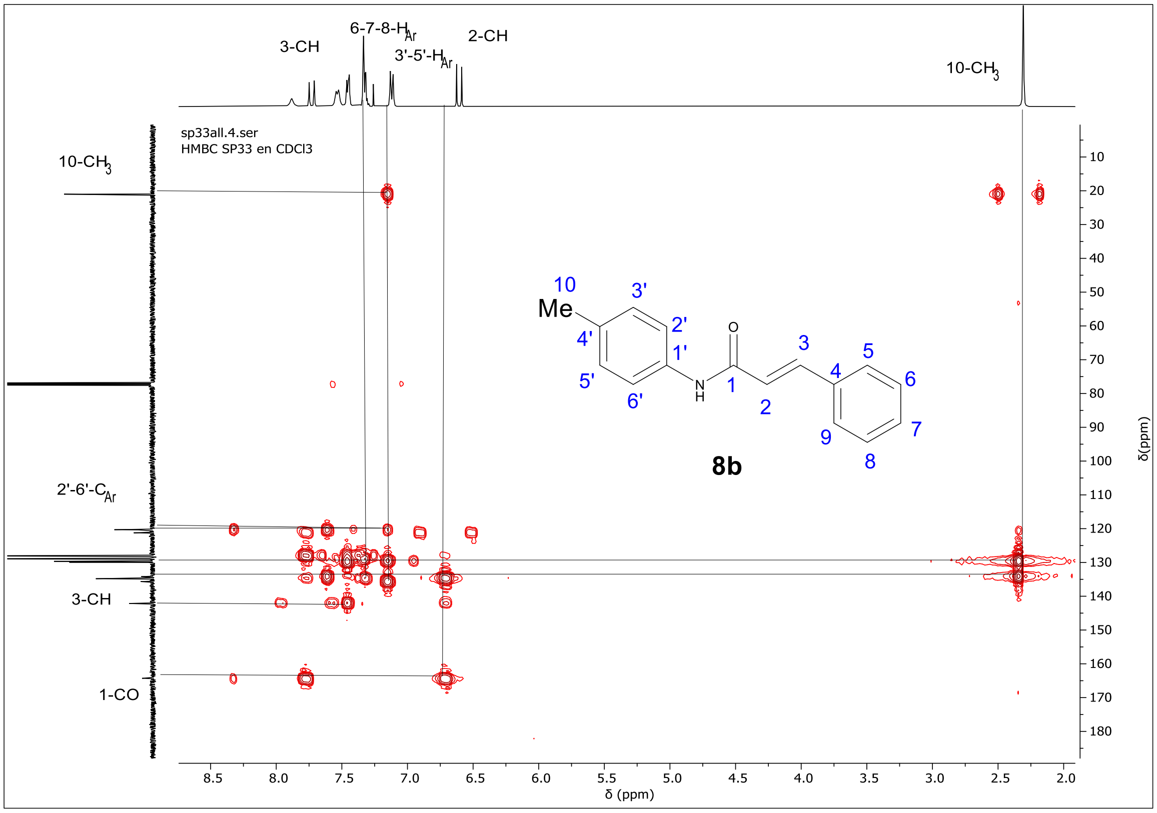
3-CH
6-7-8-HAr
3'-5'-HAr
2-CH
10-CH3
sp33all.4.ser
HMBC SP33 en CDCl3
2'-6'-CAr
1-CO
8b
δ (ppm)

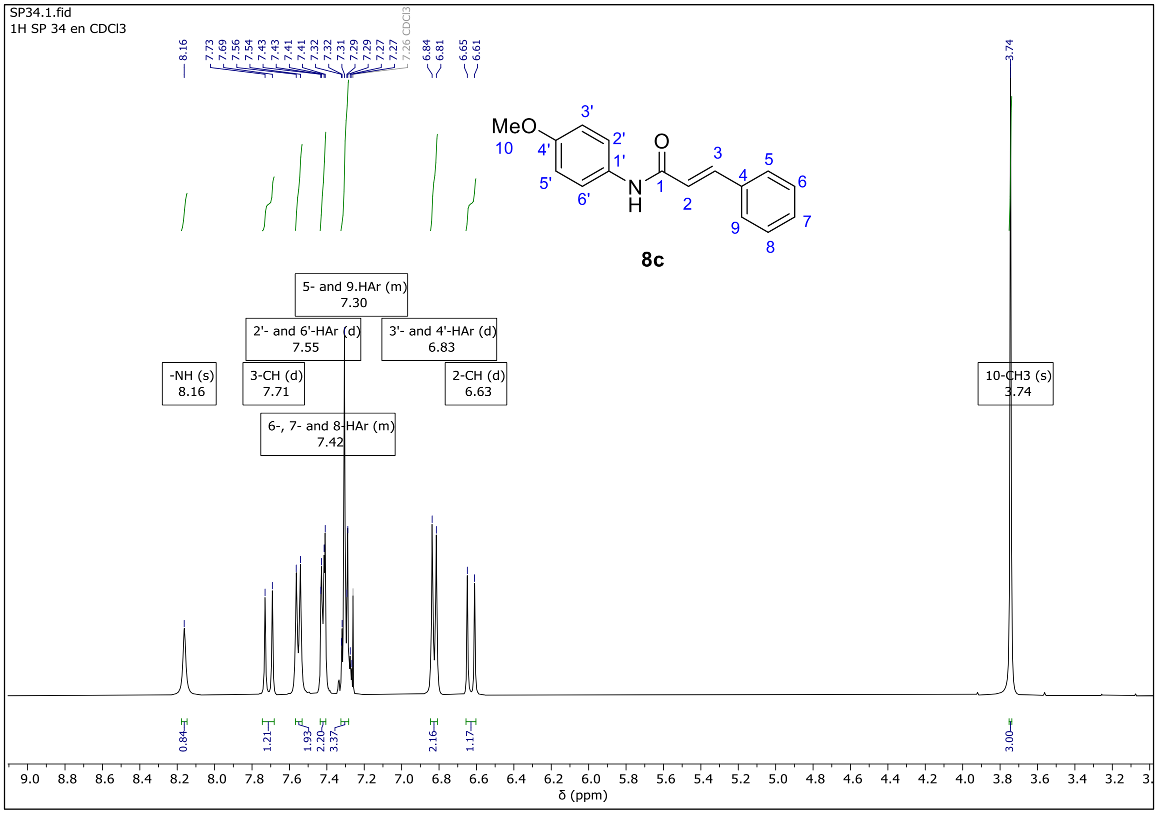

SP34.1.fid
1H SP 34 en CDCl3
8c
5- and 9.HAr (m)
7.30
2'- and 6'-HAr (d)
7.55
3'- and 4'-HAr (d)
6.83
-NH (s)
8.16
3-CH (d)
7.71
2-CH (d)
6.63
10-CH3 (s)
3.74
6-, 7- and 8-HAr (m)
7.42
δ (ppm)

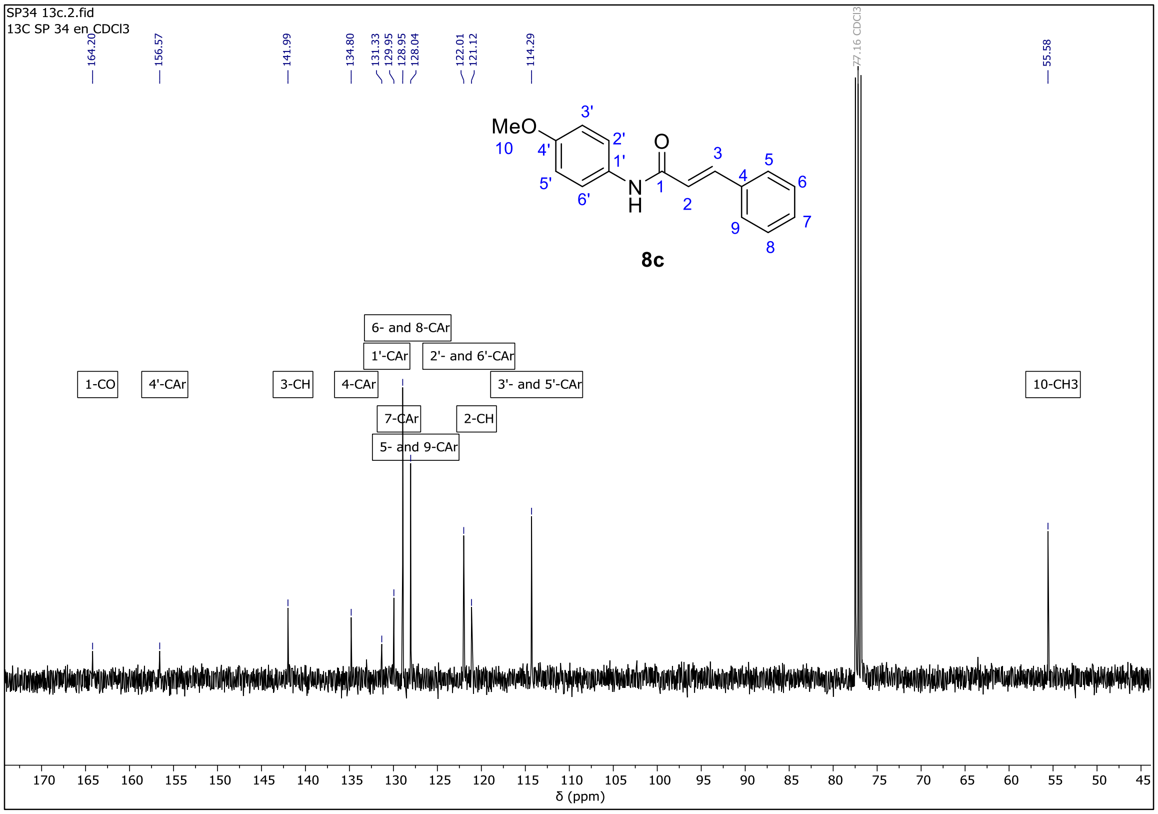

SP34 13c.2.fid
13C SP 34 en CDCl3
8c
6- and 8-CAr
1'-CAr
2'- and 6'-CAr
1-CO
4'-CAr
3-CH
4-CAr
3'- and 5'-CAr
10-CH3
7-CAr
2-CH
5- and 9-CAr
δ (ppm)

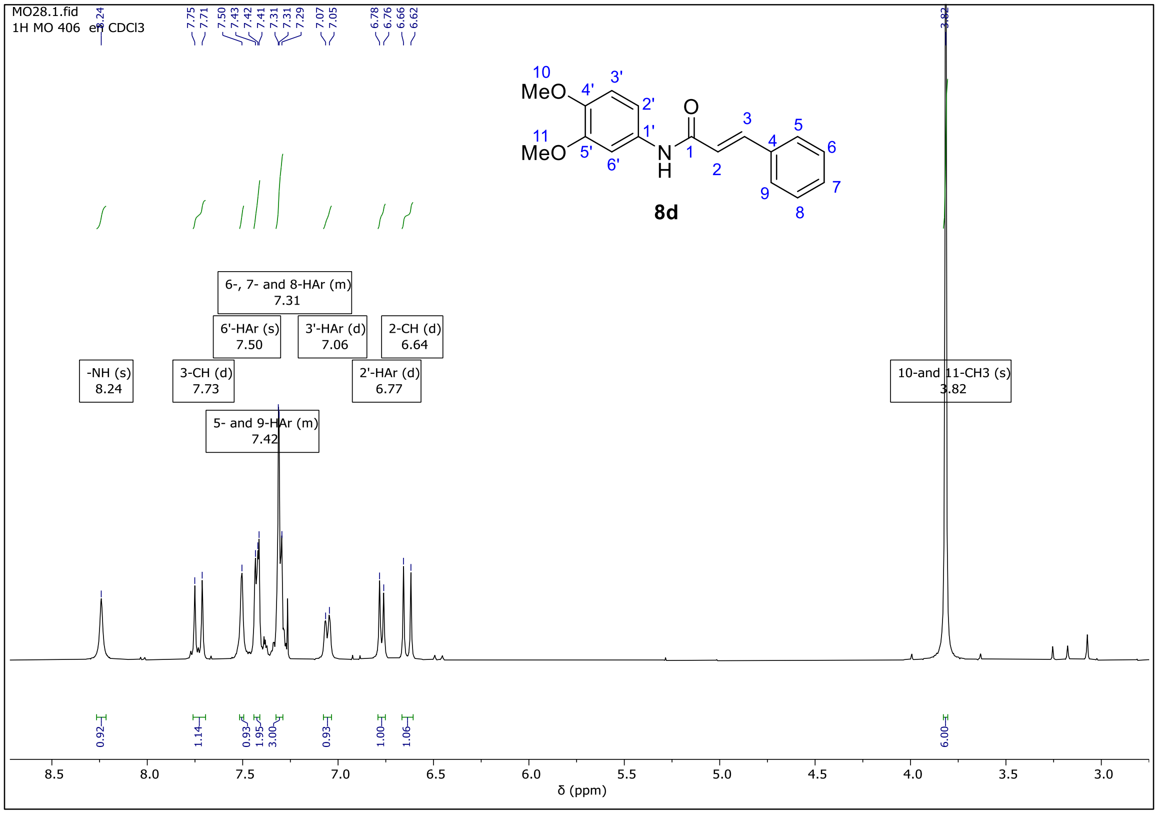
MO28.1.fid
1H MO 406 en CDCl3
8d
-NH (s) 8.24
3-CH (d) 7.73
6'-HAr (s) 7.50
5- and 9-HAr (m) 7.42
6-, 7- and 8-HAr (m) 7.31
3'-HAr (d) 7.06
2'-HAr (d) 6.77
2-CH (d) 6.64
10-and 11-CH3 (s) 3.82
δ (ppm)

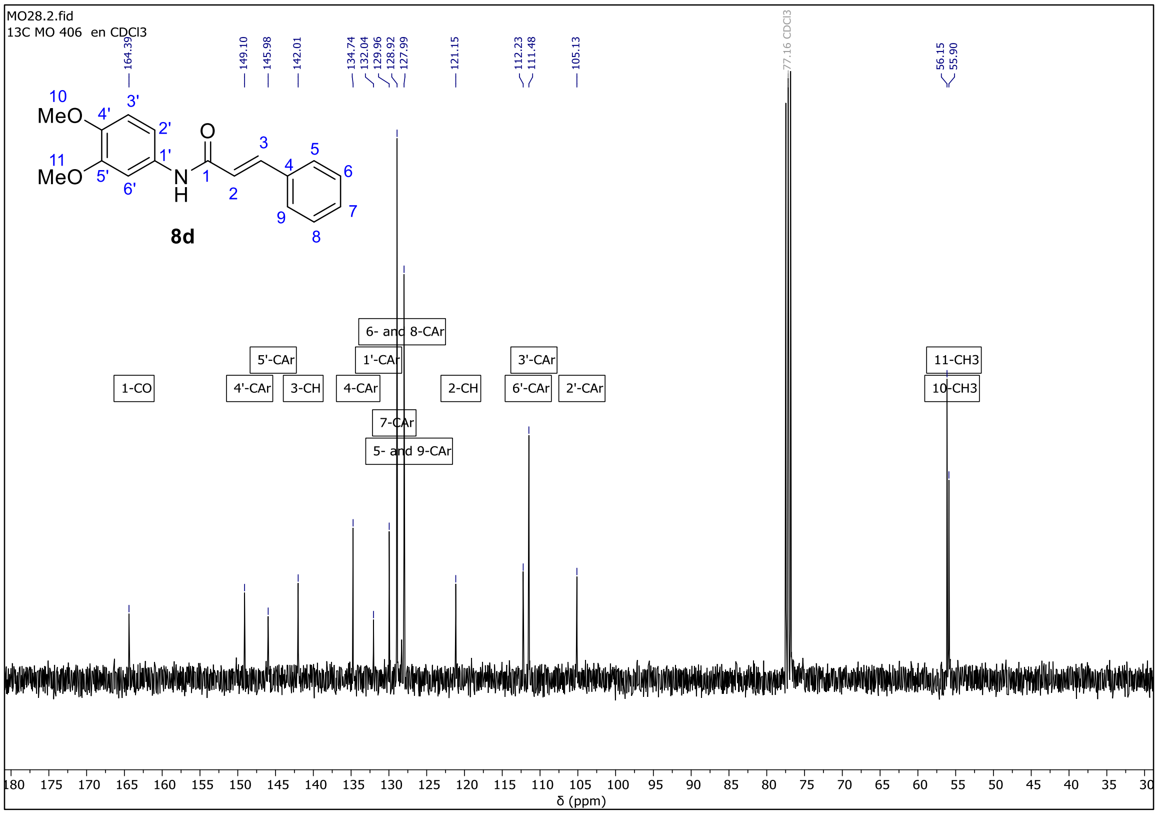
MO28.2.fid
13C MO 406 en CDCl3
8d
1-CO
4'-CAr
5'-CAr
3-CH
4-CAr
1'-CAr
6- and 8-CAr
7-CAr
5- and 9-CAr
2-CH
3'-CAr
6'-CAr
2'-CAr
11-CH3
10-CH3
δ (ppm)

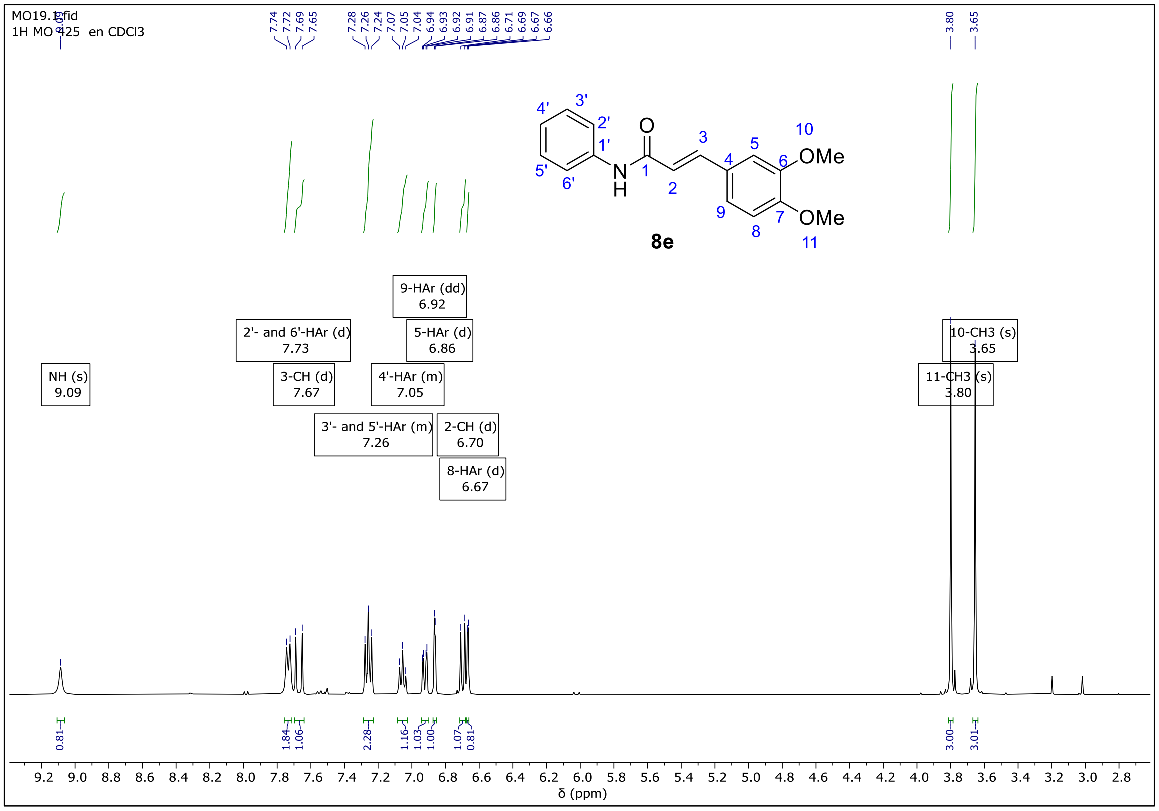
MO19.1.fid
1H MO 425 en CDCl3
7.74 7.72 7.69 7.65 7.28 7.26 7.24 7.07 7.05 7.04 6.94 6.93 6.92 6.91 6.87 6.86 6.71 6.69 6.67 6.66
3.80 3.65
8e
OMe
OMe
NH (s) 9.09
2'- and 6'-HAr (d) 7.73
3-CH (d) 7.67
3'- and 5'-HAr (m) 7.26
4'-HAr (m) 7.05
9-HAr (dd) 6.92
5-HAr (d) 6.86
2-CH (d) 6.70
8-HAr (d) 6.67
11-CH3 (s) 3.80
10-CH3 (s) 3.65
0.81 1.84 1.06 2.28 1.16 1.03 1.00 1.07 0.81 3.00 3.01
δ (ppm)

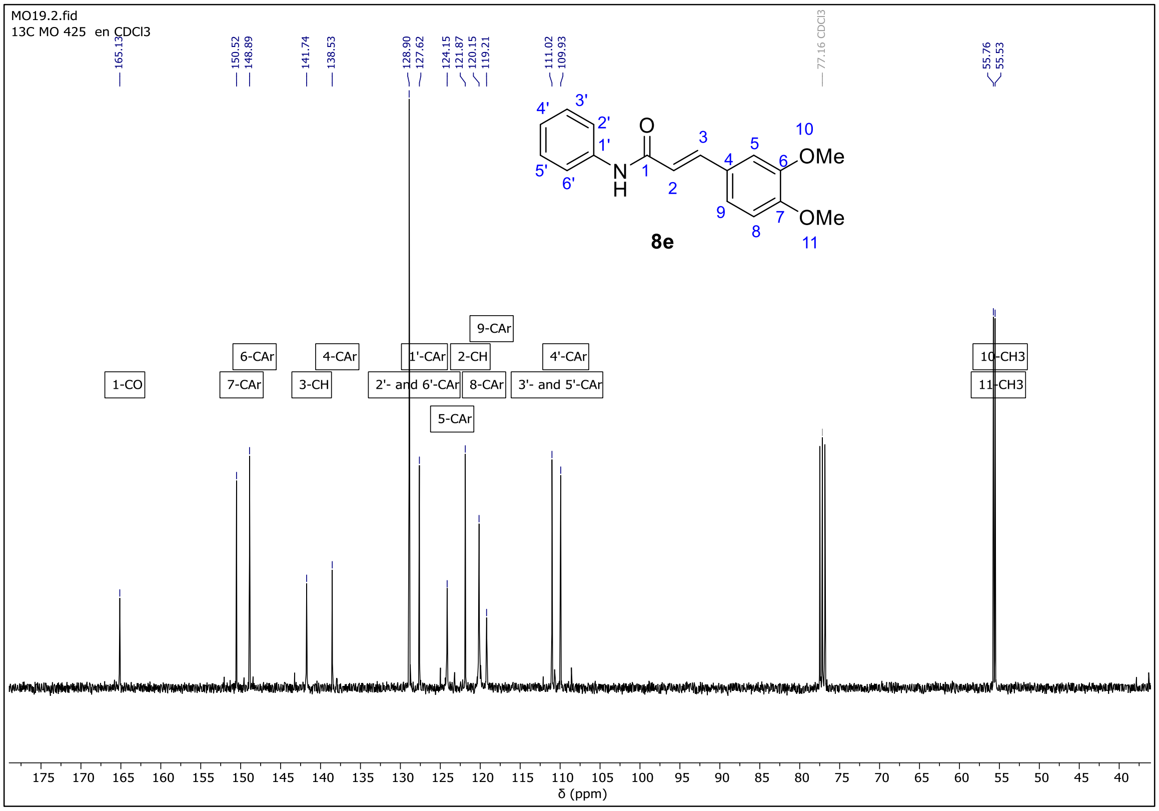
MO19.2.fid
13C MO 425 en CDCl3
165.13 150.52 148.89 141.74 138.53 128.90 127.62 124.15 121.87 120.15 119.21 111.02 109.93 77.16 CDCl3 55.76 55.53
8e
OMe
OMe
1-CO
6-CAr
7-CAr
4-CAr
3-CH
1'-CAr
2'- and 6'-CAr
2-CH
9-CAr
8-CAr
5-CAr
4'-CAr
3'- and 5'-CAr
10-CH3
11-CH3
δ (ppm)

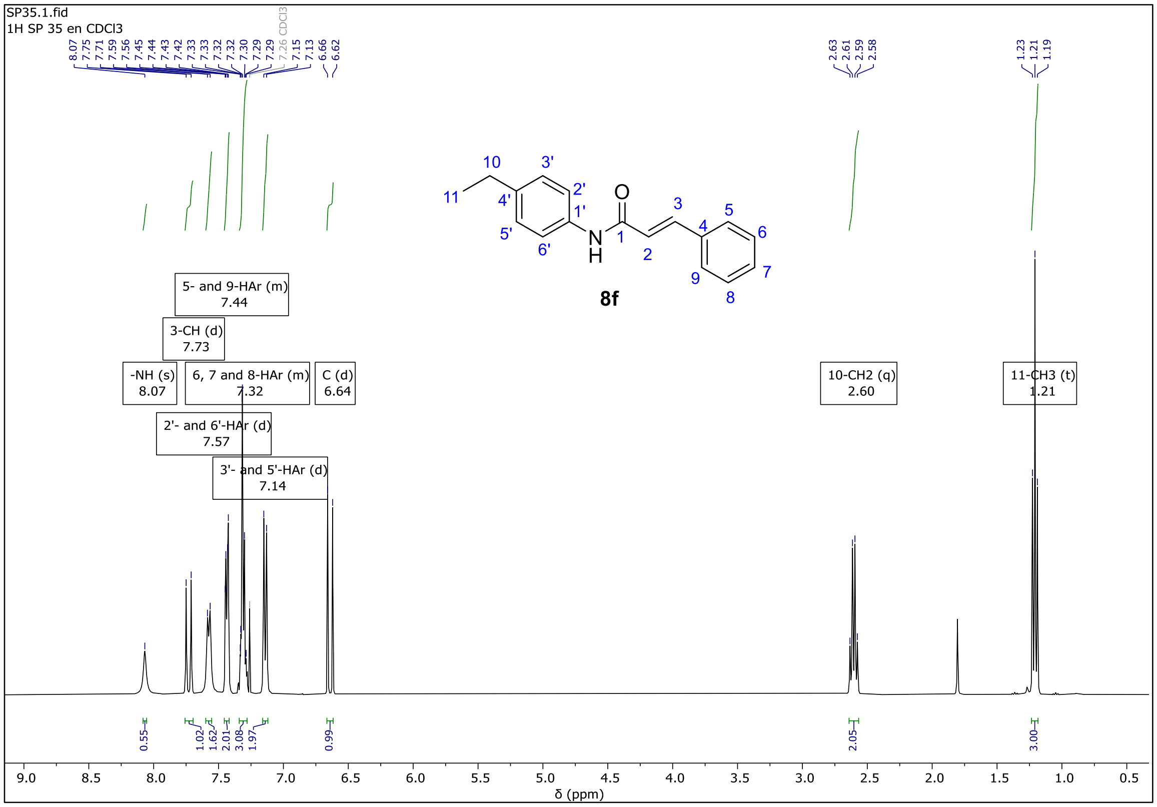
SP35.1.fid
1H SP 35 en CDCl3
8f
5- and 9-HAr (m) 7.44
3-CH (d) 7.73
-NH (s) 8.07
6, 7 and 8-HAr (m) 7.32
C (d) 6.64
2'- and 6'-HAr (d) 7.57
3'- and 5'-HAr (d) 7.14
10-CH2 (q) 2.60
11-CH3 (t) 1.21
δ (ppm)

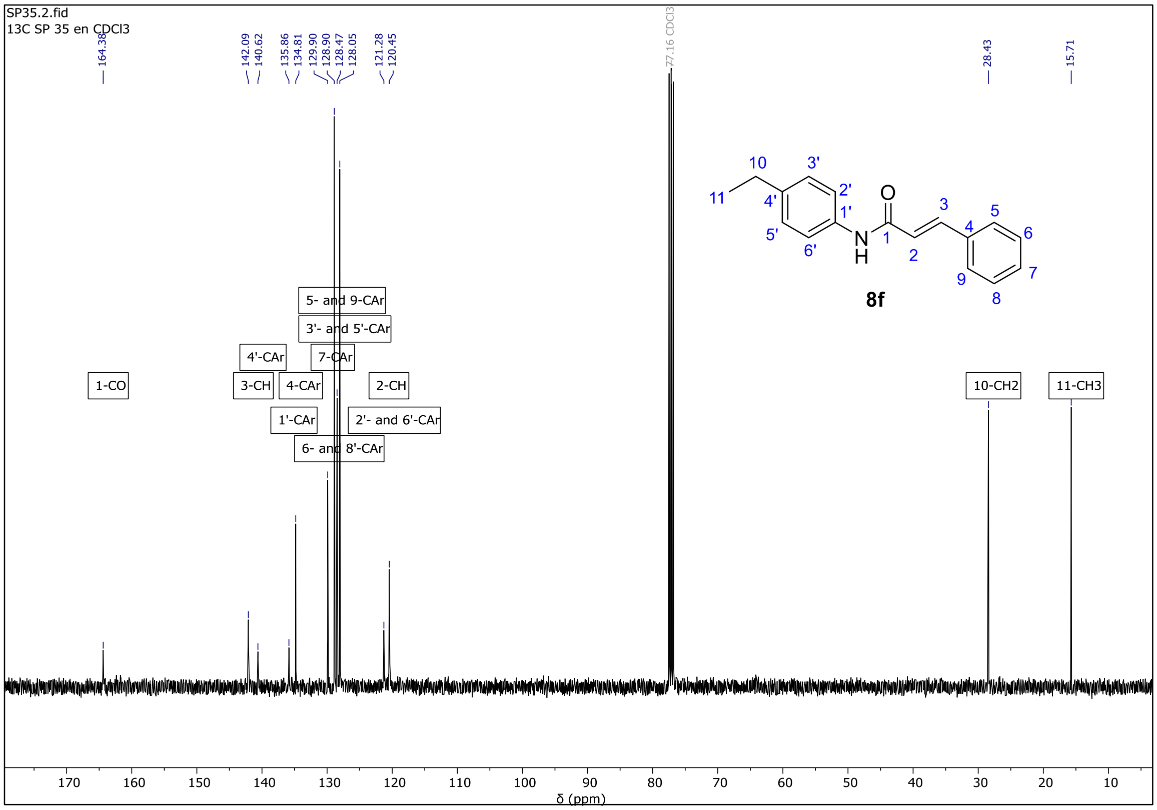
SP35.2.fid
13C SP 35 en CDCl3
8f
5- and 9-CAr
3'- and 5'-CAr
4'-CAr
7-CAr
1-CO
3-CH
4-CAr
2-CH
1'-CAr
2'- and 6'-CAr
6- and 8'-CAr
10-CH2
11-CH3
δ (ppm)

8. Figures S17-S32 of the respective ^{1}H and ^{13}C NMR spectra of all synthesized *N*-(2-bromobenzyl)-*N*-phenylcinnamamides 7a-f

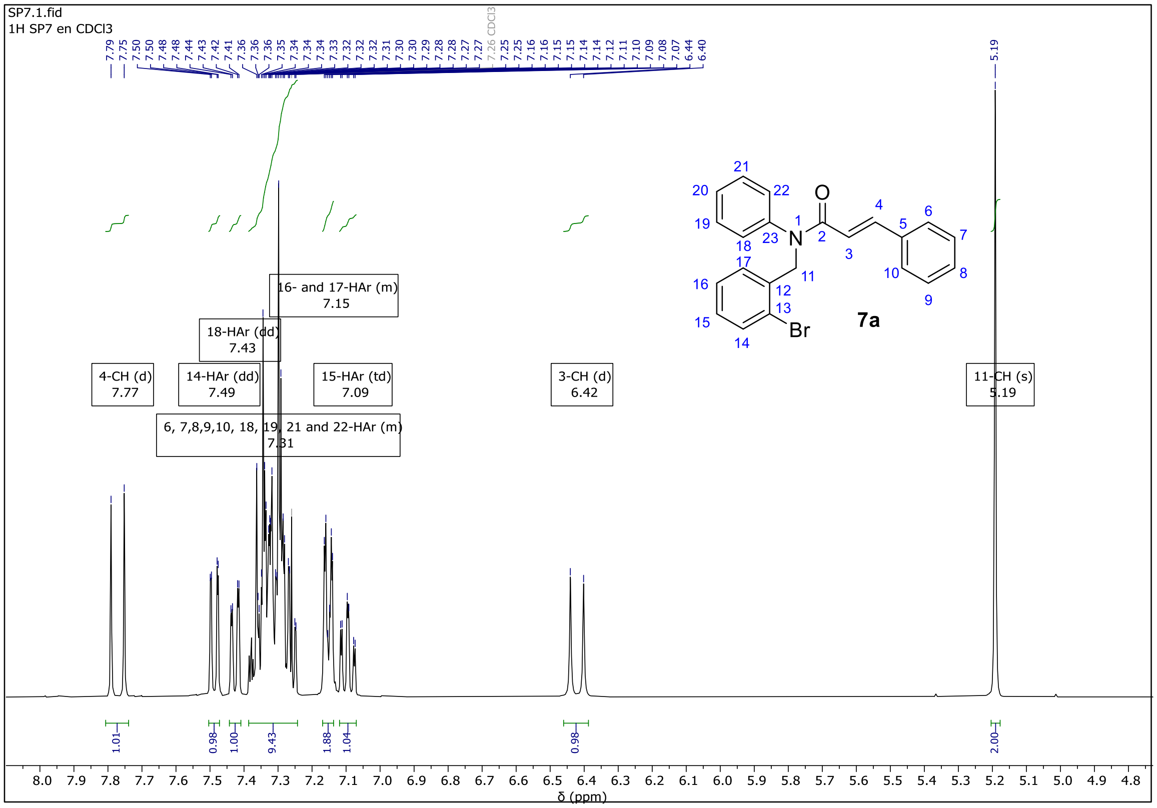

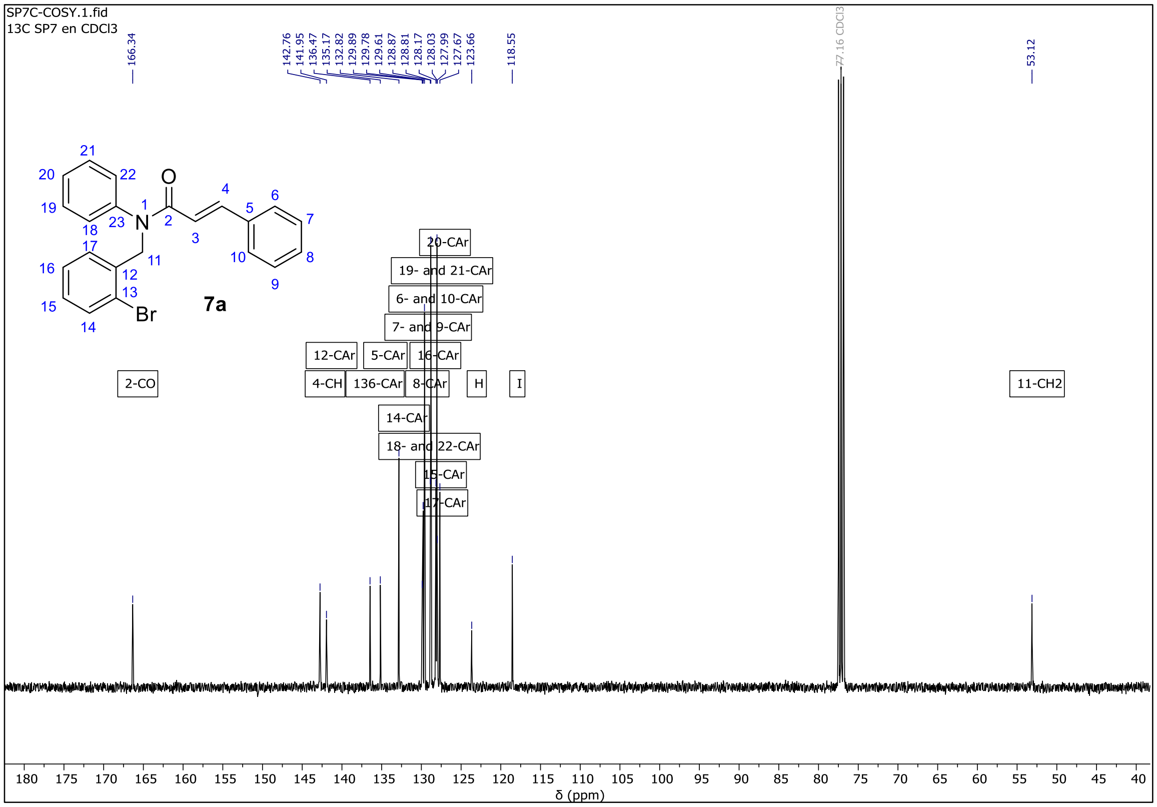

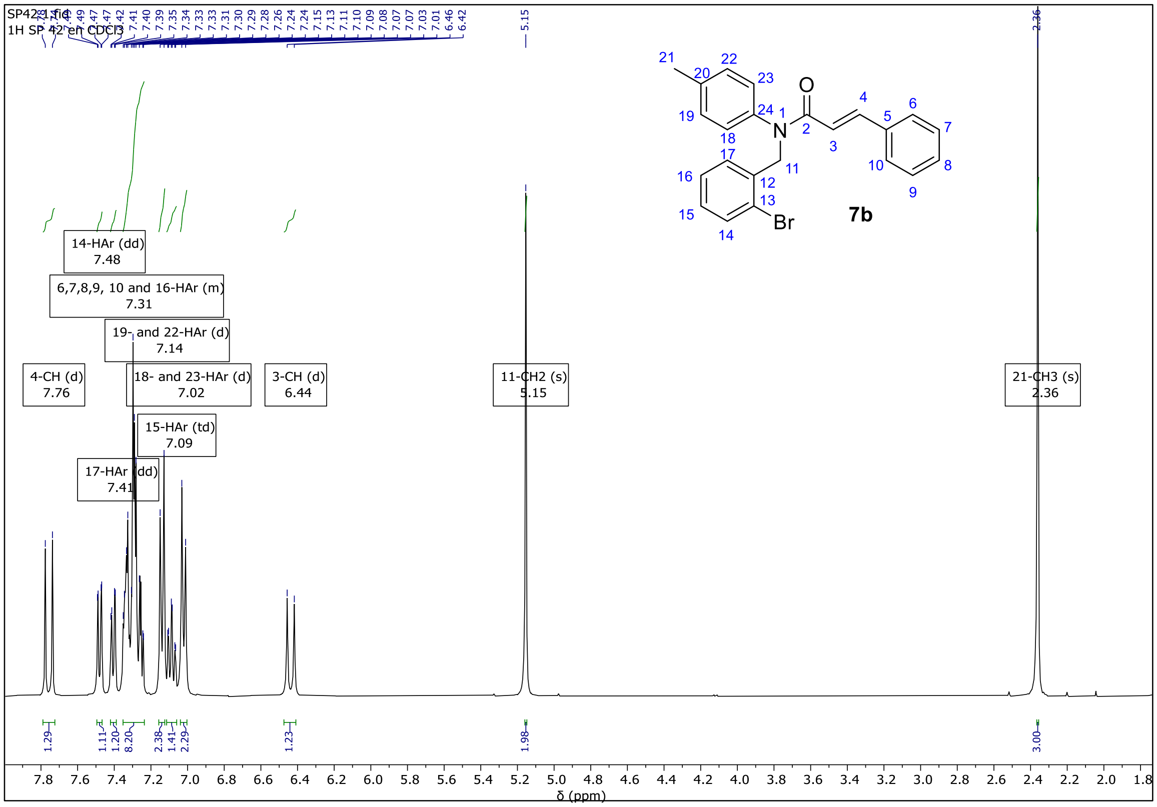
SP42.1.fid
1H SP 42 en CDCl3
7b
14-HAr (dd)
7.48
6,7,8,9, 10 and 16-HAr (m)
7.31
19- and 22-HAr (d)
7.14
4-CH (d)
7.76
18- and 23-HAr (d)
7.02
3-CH (d)
6.44
11-CH2 (s)
5.15
21-CH3 (s)
2.36
15-HAr (td)
7.09
17-HAr (dd)
7.41
δ (ppm)

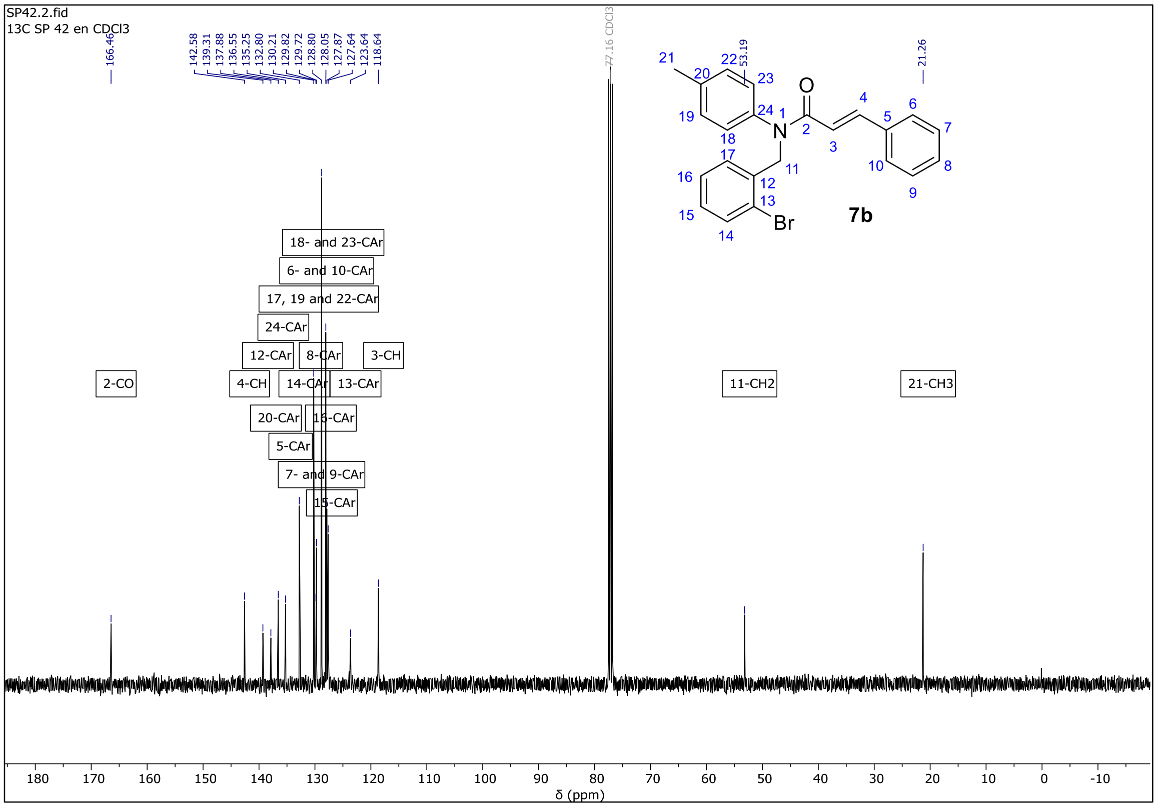
SP42.2.fid
13C SP 42 en CDCl3
7b
18- and 23-CAr
6- and 10-CAr
17, 19 and 22-CAr
24-CAr
12-CAr
8-CAr
3-CH
2-CO
4-CH
14-CAr
13-CAr
11-CH2
21-CH3
20-CAr
16-CAr
5-CAr
7- and 9-CAr
15-CAr
δ (ppm)

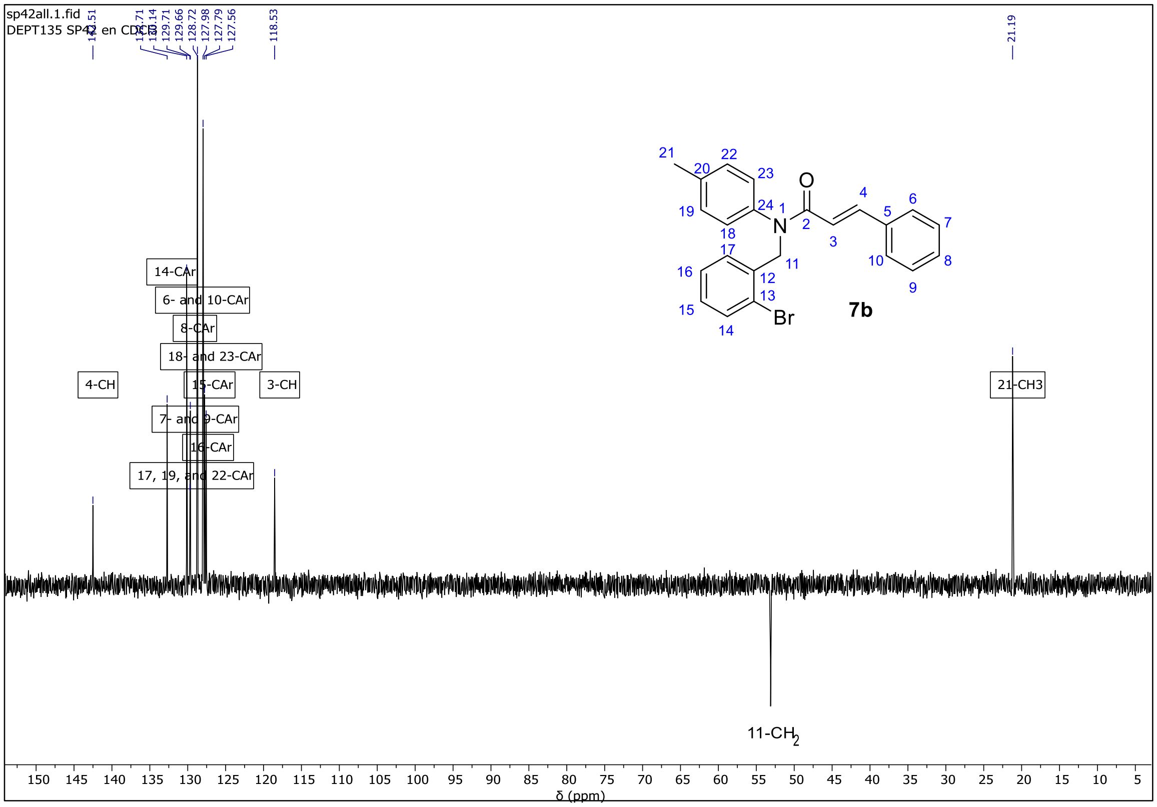
sp42all.1.fid
DEPT135 SP42 en CDCl3
14-CAr
6- and 10-CAr
8-CAr
18- and 23-CAr
4-CH
15-CAr
3-CH
21-CH3
7- and 9-CAr
16-CAr
17, 19, and 22-CAr
11-CH2
7b
Br
δ (ppm)

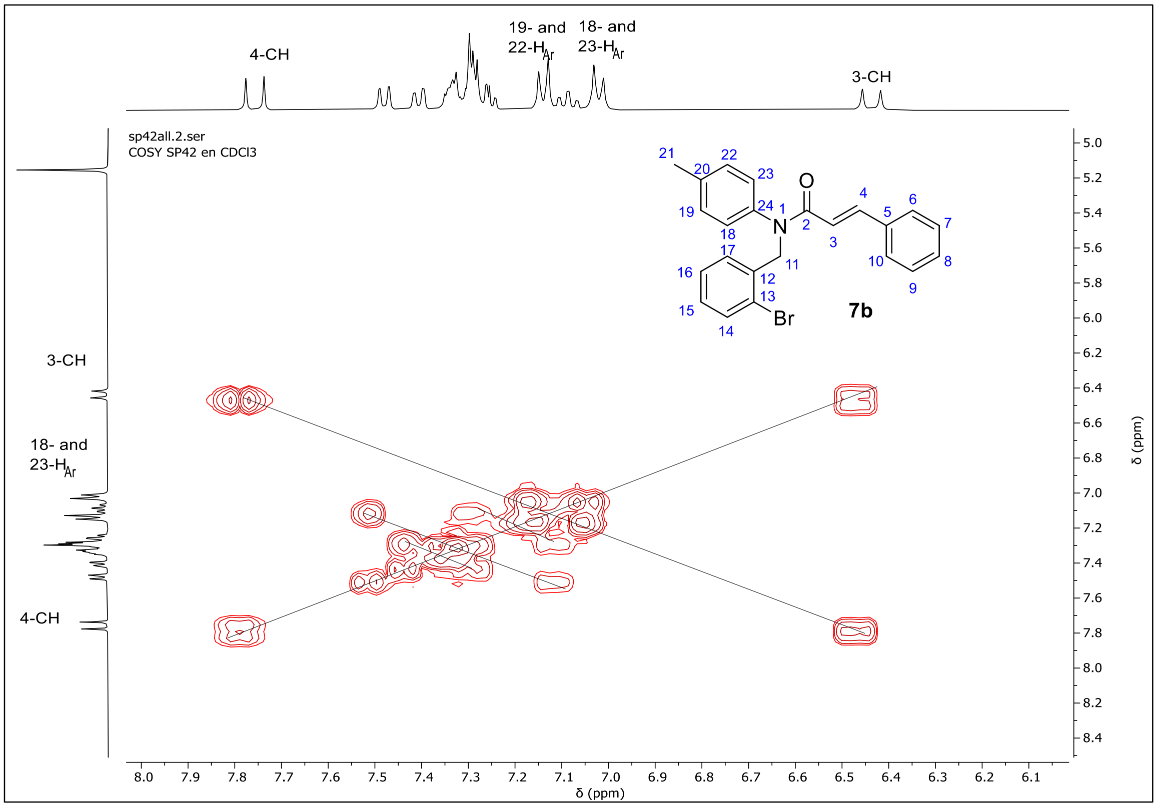
4-CH
19- and 22-HAr
18- and 23-HAr
3-CH
sp42all.2.ser
COSY SP42 en CDCl3
7b
Br
δ (ppm)

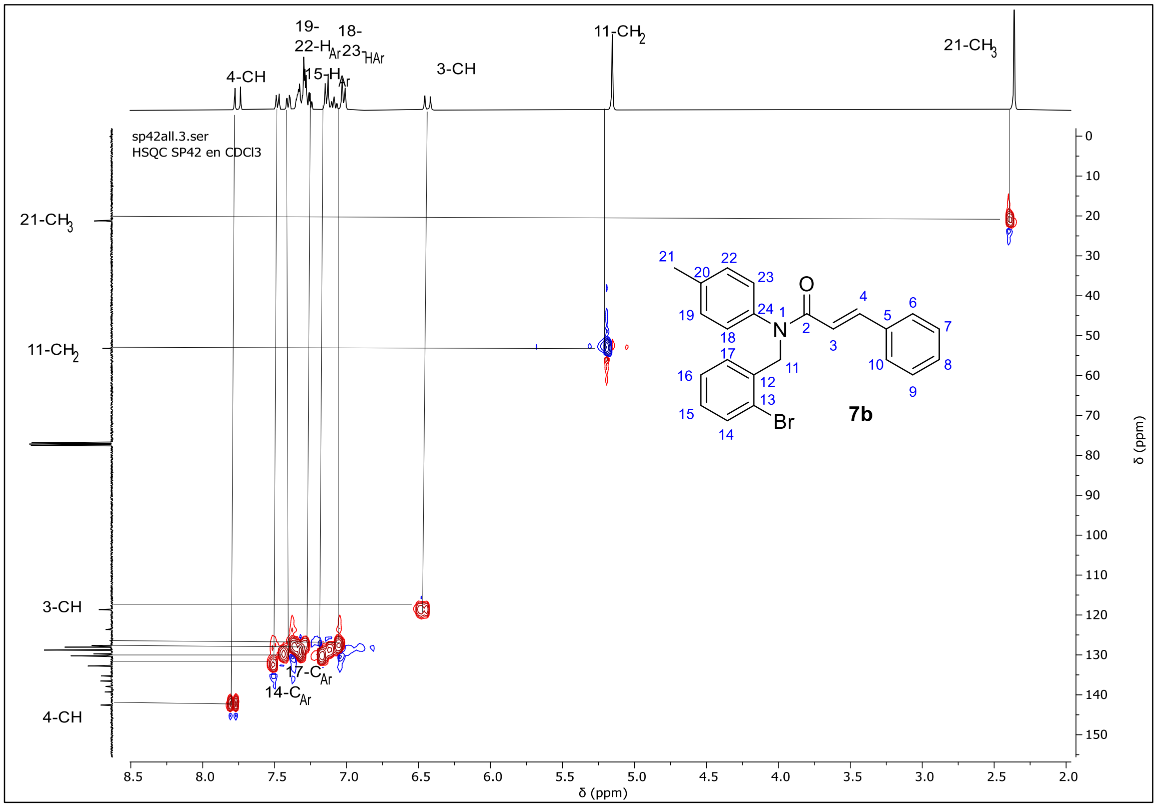
sp42all.3.ser
HSQC SP42 en CDCl3
21-CH3
11-CH2
3-CH
4-CH
19-22-HAr
18-23-HAr
15-HAr
17-CAr
14-CAr
7b
Br
O
N
δ (ppm)

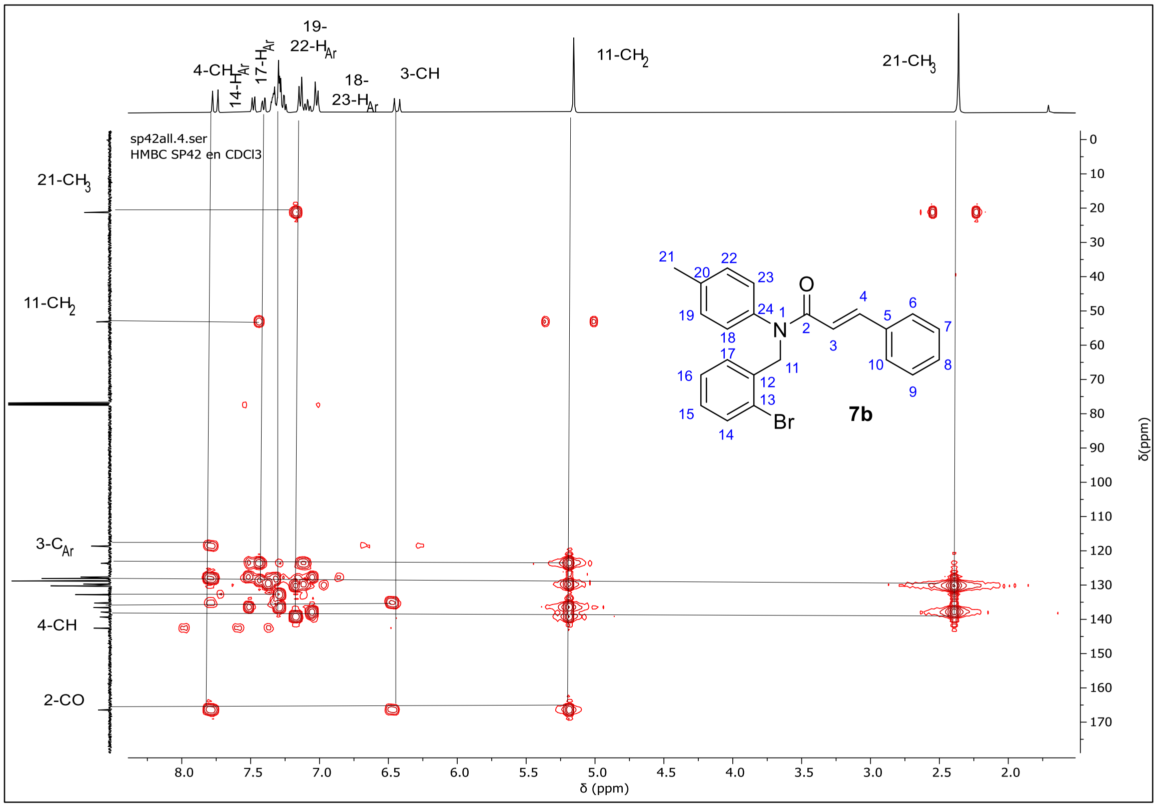
sp42all.4.ser
HMBC SP42 en CDCl3
21-CH3
11-CH2
3-CAr
4-CH
2-CO
3-CH
14-HAr
17-HAr
19-22-HAr
18-23-HAr
7b
Br
O
N
δ (ppm)

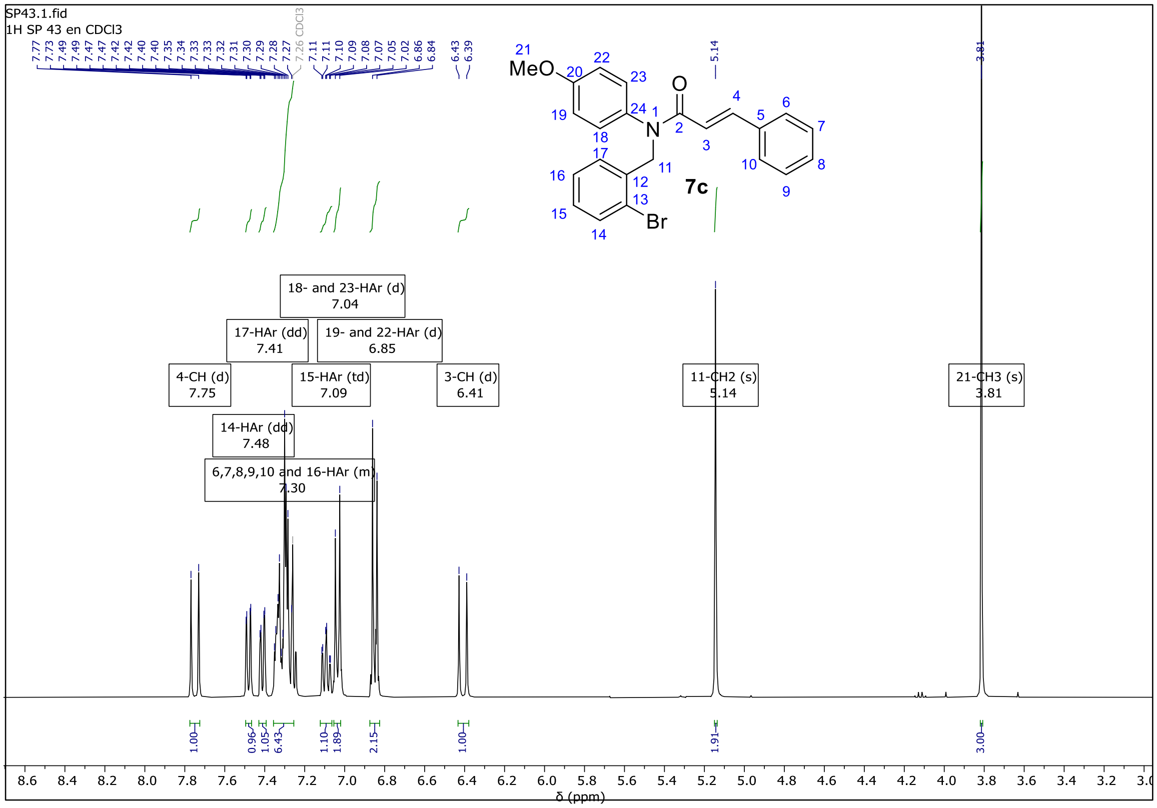
SP43.1.fid
1H SP 43 en CDCl3
7c
MeO
Br
18- and 23-HAr (d)
7.04
17-HAr (dd)
7.41
19- and 22-HAr (d)
6.85
4-CH (d)
7.75
15-HAr (td)
7.09
3-CH (d)
6.41
11-CH2 (s)
5.14
21-CH3 (s)
3.81
14-HAr (dd)
7.48
6,7,8,9,10 and 16-HAr (m)
7.30
δ (ppm)

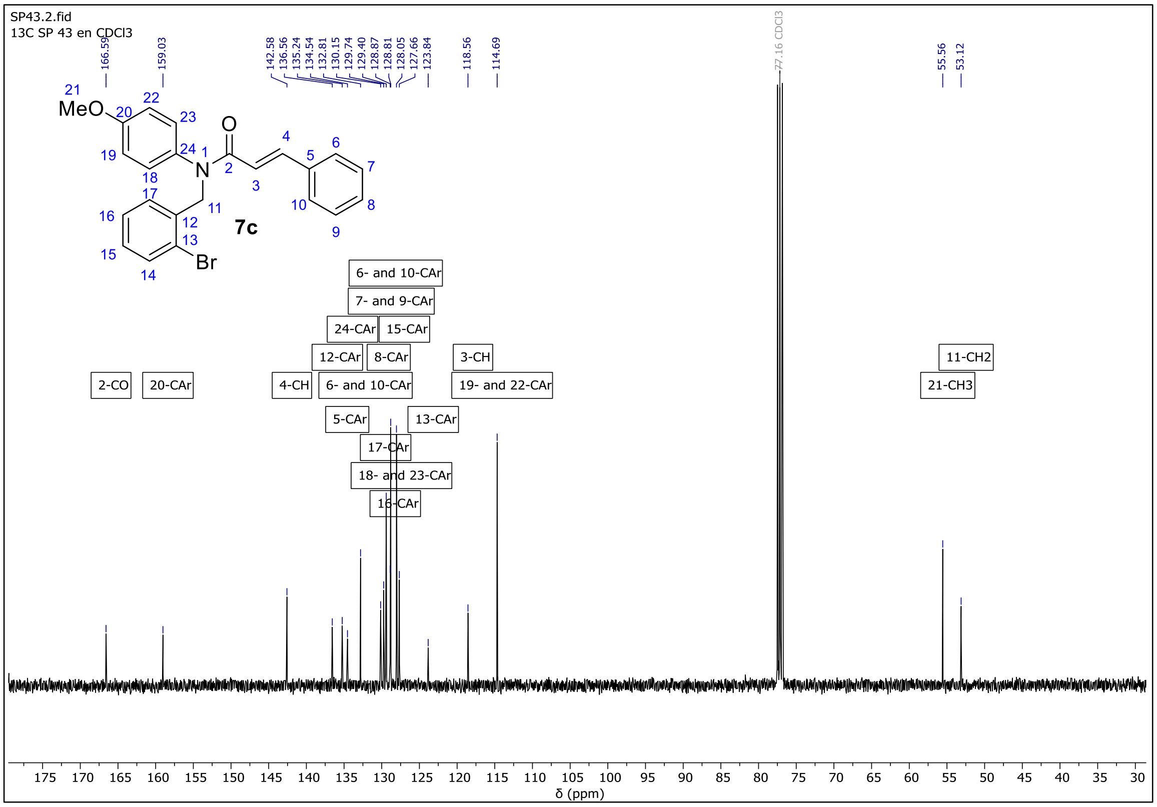
SP43.2.fid
13C SP 43 en CDCl3
7c
MeO
Br
6- and 10-CAr
7- and 9-CAr
24-CAr
15-CAr
12-CAr
8-CAr
3-CH
11-CH2
2-CO
20-CAr
4-CH
6- and 10-CAr
19- and 22-CAr
21-CH3
5-CAr
13-CAr
17-CAr
18- and 23-CAr
16-CAr
δ (ppm)

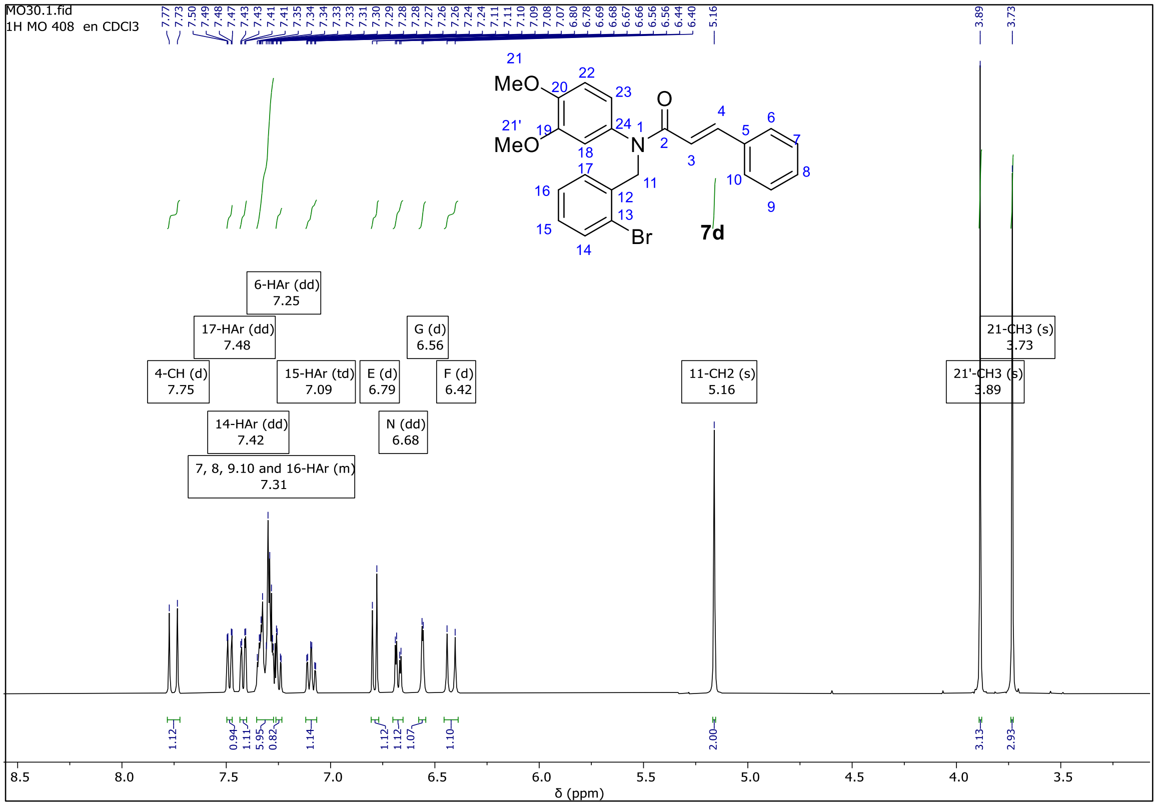
MO30.1.fid
1H MO 408 en CDCl3
7d
6-HAr (dd)
7.25
17-HAr (dd)
7.48
G (d)
6.56
21-CH3 (s)
3.73
4-CH (d)
7.75
15-HAr (td)
7.09
E (d)
6.79
F (d)
6.42
11-CH2 (s)
5.16
21'-CH3 (s)
3.89
14-HAr (dd)
7.42
N (dd)
6.68
7, 8, 9.10 and 16-HAr (m)
7.31
δ (ppm)

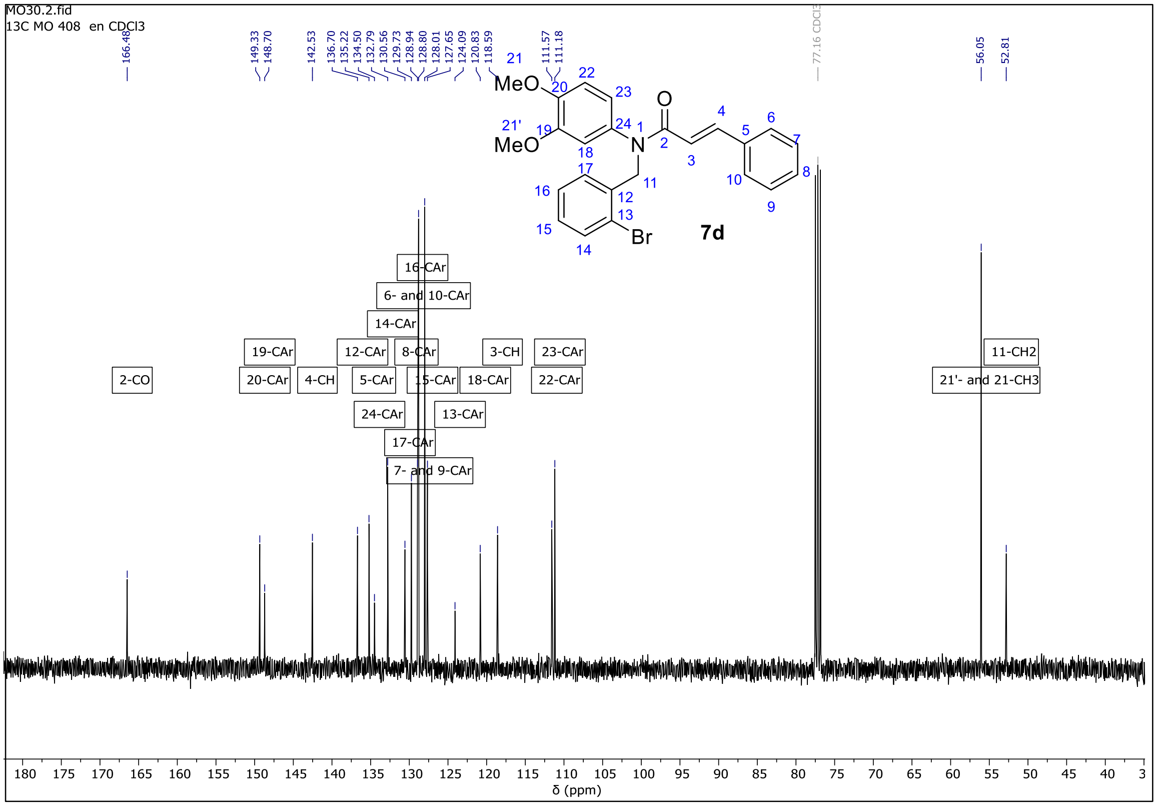
MO30.2.fid
13C MO 408 en CDCl3
7d
16-CAr
6- and 10-CAr
14-CAr
19-CAr
12-CAr
8-CAr
3-CH
23-CAr
11-CH2
2-CO
20-CAr
4-CH
5-CAr
15-CAr
18-CAr
22-CAr
21'- and 21-CH3
24-CAr
13-CAr
17-CAr
7- and 9-CAr
δ (ppm)

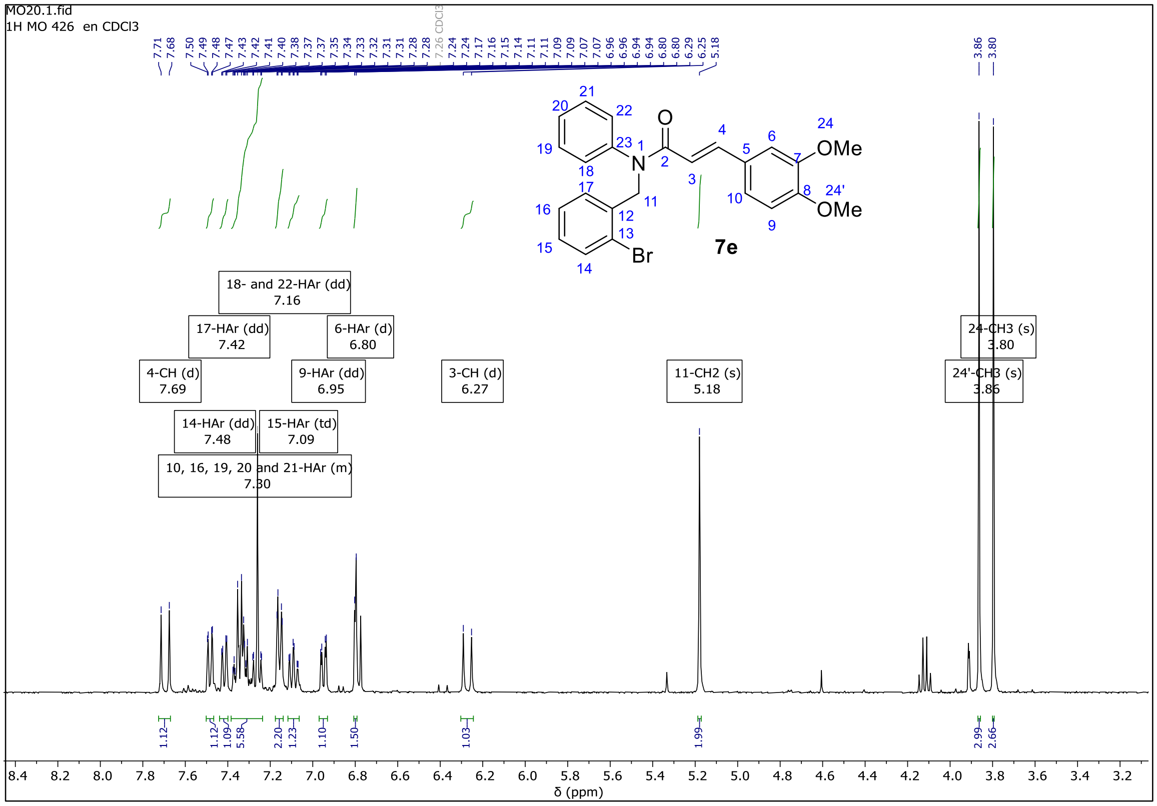
MO20.1.fid
1H MO 426 en CDCl3
7e
18- and 22-HAr (dd)
7.16
17-HAr (dd)
7.42
6-HAr (d)
6.80
4-CH (d)
7.69
9-HAr (dd)
6.95
3-CH (d)
6.27
11-CH2 (s)
5.18
24-CH3 (s)
3.80
24'-CH3 (s)
3.86
14-HAr (dd)
7.48
15-HAr (td)
7.09
10, 16, 19, 20 and 21-HAr (m)
7.30
δ (ppm)

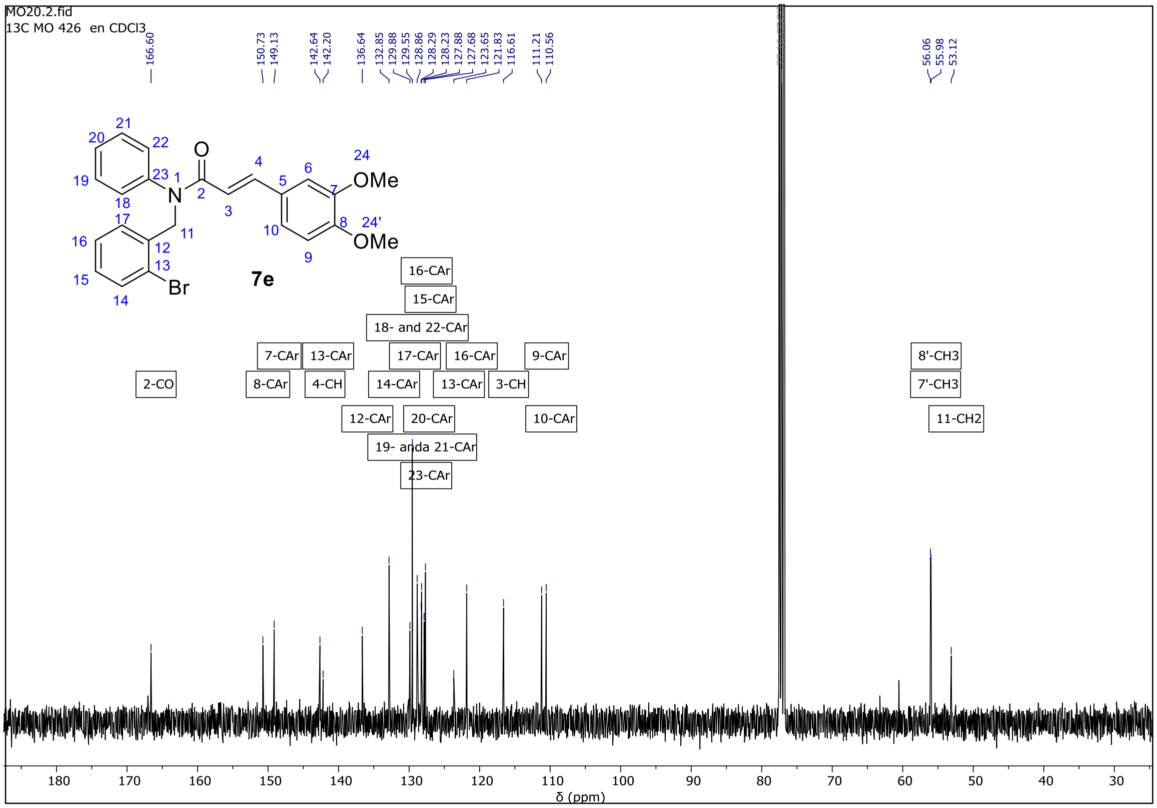
MO20.2.fid
13C MO 426 en CDCl3
7e
16-CAr
15-CAr
18- and 22-CAr
7-CAr
13-CAr
17-CAr
16-CAr
9-CAr
8'-CH3
2-CO
8-CAr
4-CH
14-CAr
13-CAr
3-CH
7'-CH3
12-CAr
20-CAr
10-CAr
11-CH2
19- anda 21-CAr
23-CAr
δ (ppm)

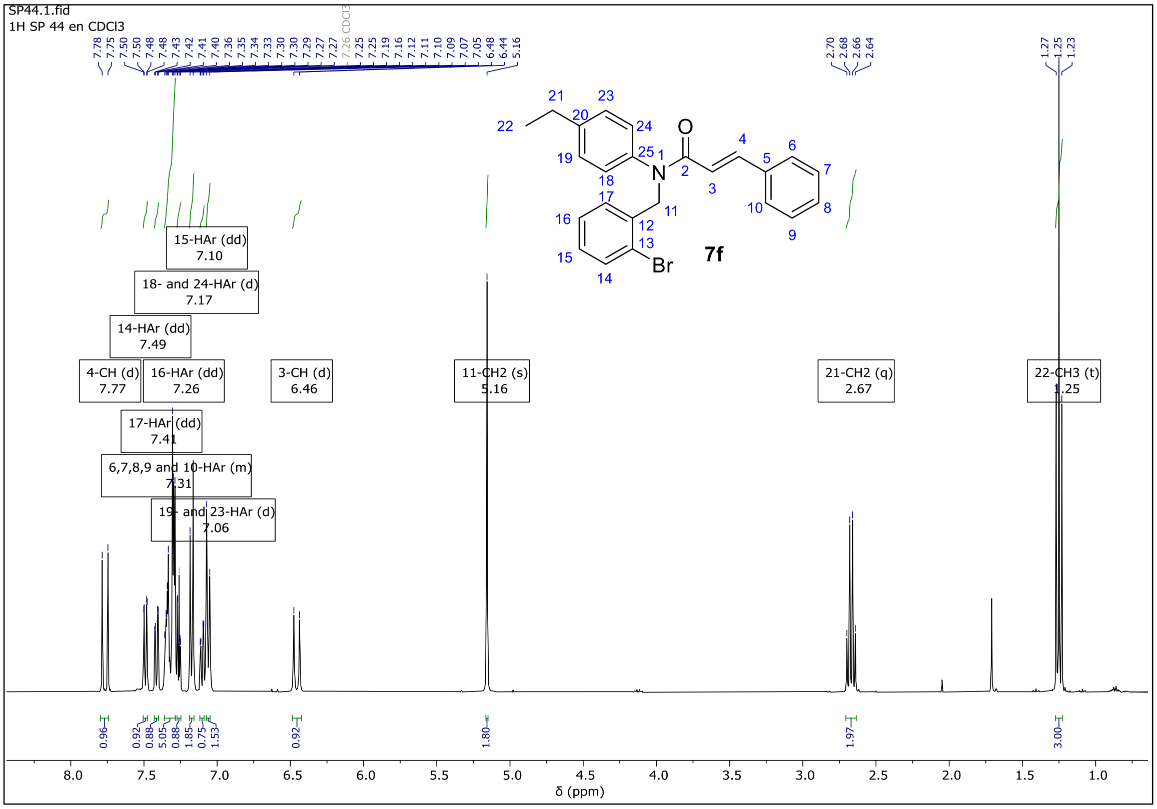
SP44.1.fid
1H SP 44 en CDCl3
7f
15-HAr (dd) 7.10
18- and 24-HAr (d) 7.17
14-HAr (dd) 7.49
4-CH (d) 7.77
16-HAr (dd) 7.26
3-CH (d) 6.46
11-CH2 (s) 5.16
21-CH2 (q) 2.67
22-CH3 (t) 1.25
17-HAr (dd) 7.41
6,7,8,9 and 10-HAr (m) 7.31
19- and 23-HAr (d) 7.06
δ (ppm)

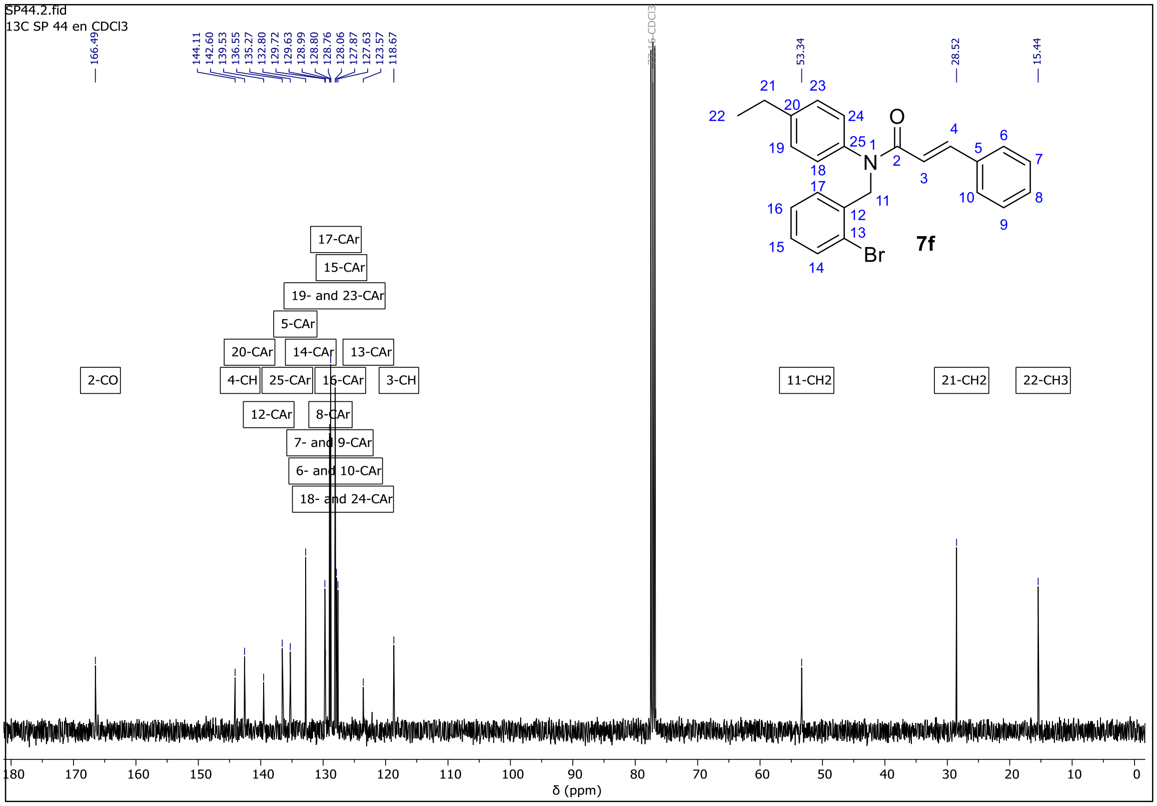
SP44.2.fid
13C SP 44 en CDCl3
166.49
53.34
28.52
15.44
7f
17-CAr
15-CAr
19- and 23-CAr
5-CAr
20-CAr
14-CAr
13-CAr
2-CO
4-CH
25-CAr
16-CAr
3-CH
11-CH2
21-CH2
22-CH3
12-CAr
8-CAr
7- and 9-CAr
6- and 10-CAr
18- and 24-CAr
δ (ppm)

9. Figures S33-S49 of the respective ^{1}H and ^{13}C NMR spectra of all synthesized *N*-benzyl-4-phenyl-3,4-dihydroquinolin-2(1*H*)-ones 6a-f

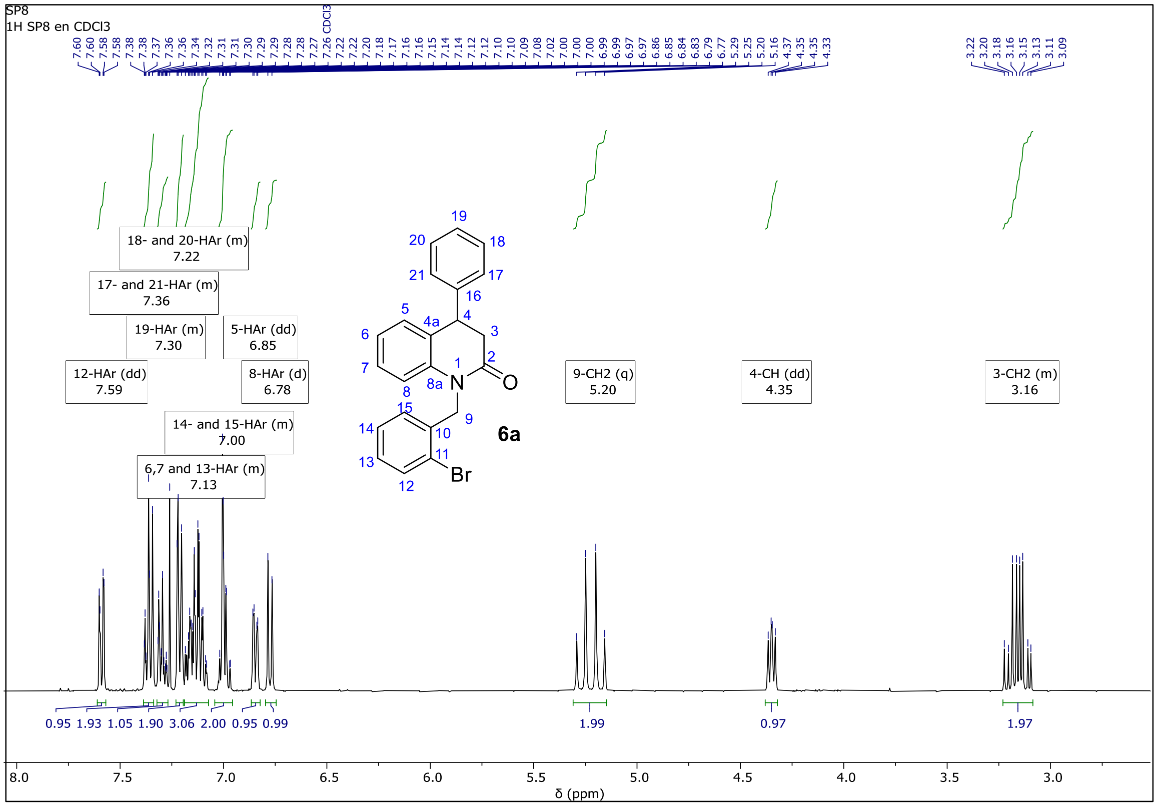

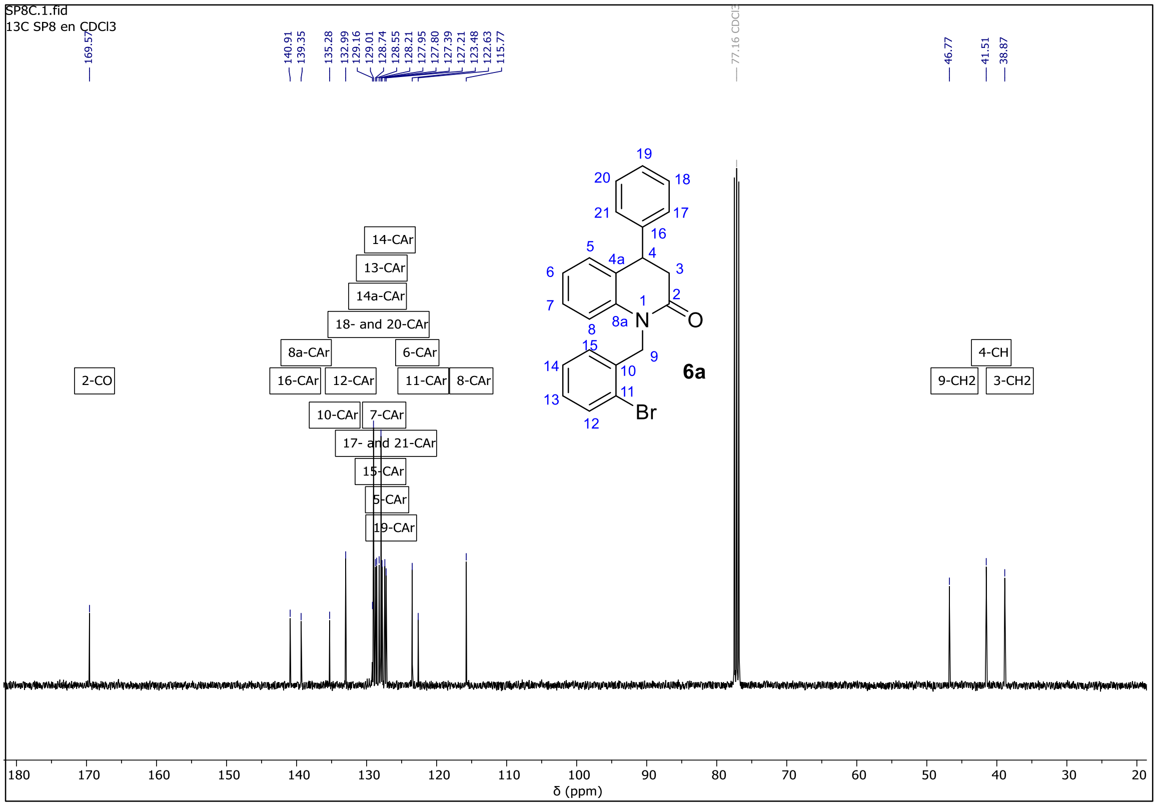

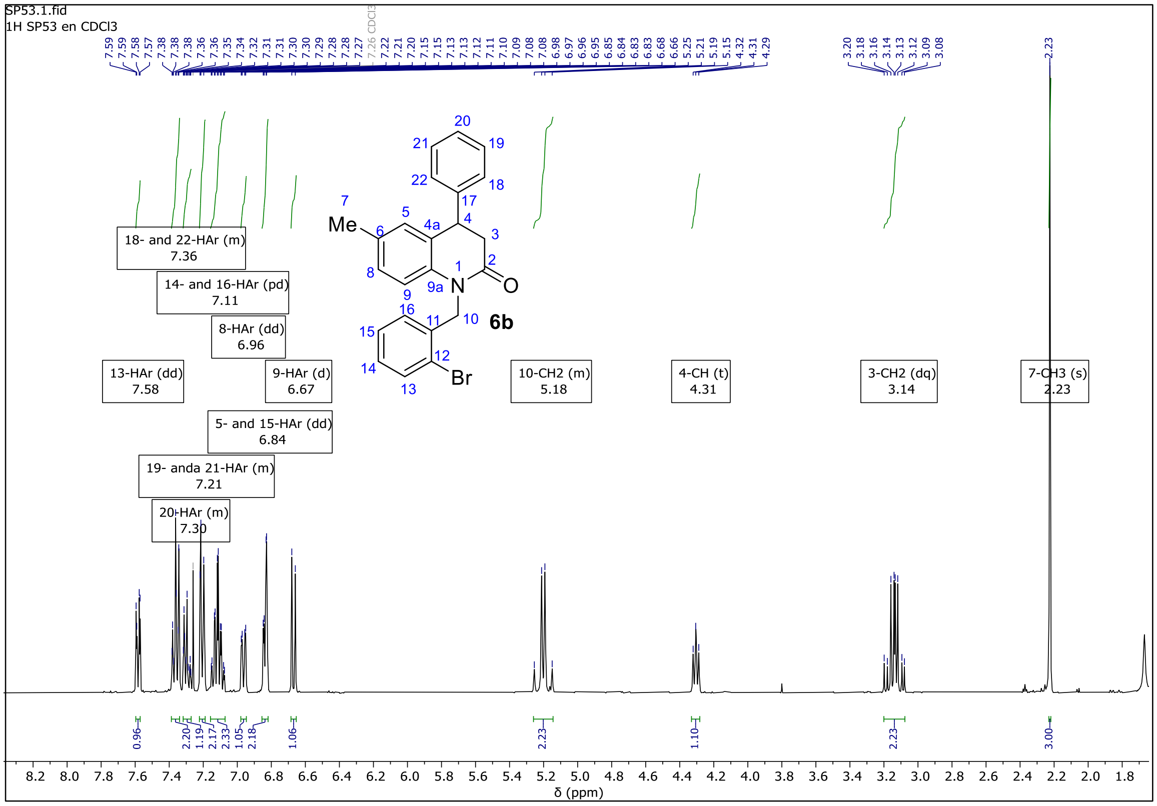
SP53.1.fid
1H SP53 en CDCl3
6b
18- and 22-HAr (m)
7.36
14- and 16-HAr (pd)
7.11
8-HAr (dd)
6.96
13-HAr (dd)
7.58
9-HAr (d)
6.67
10-CH2 (m)
5.18
4-CH (t)
4.31
3-CH2 (dq)
3.14
7-CH3 (s)
2.23
5- and 15-HAr (dd)
6.84
19- anda 21-HAr (m)
7.21
20-HAr (m)
7.30
δ (ppm)

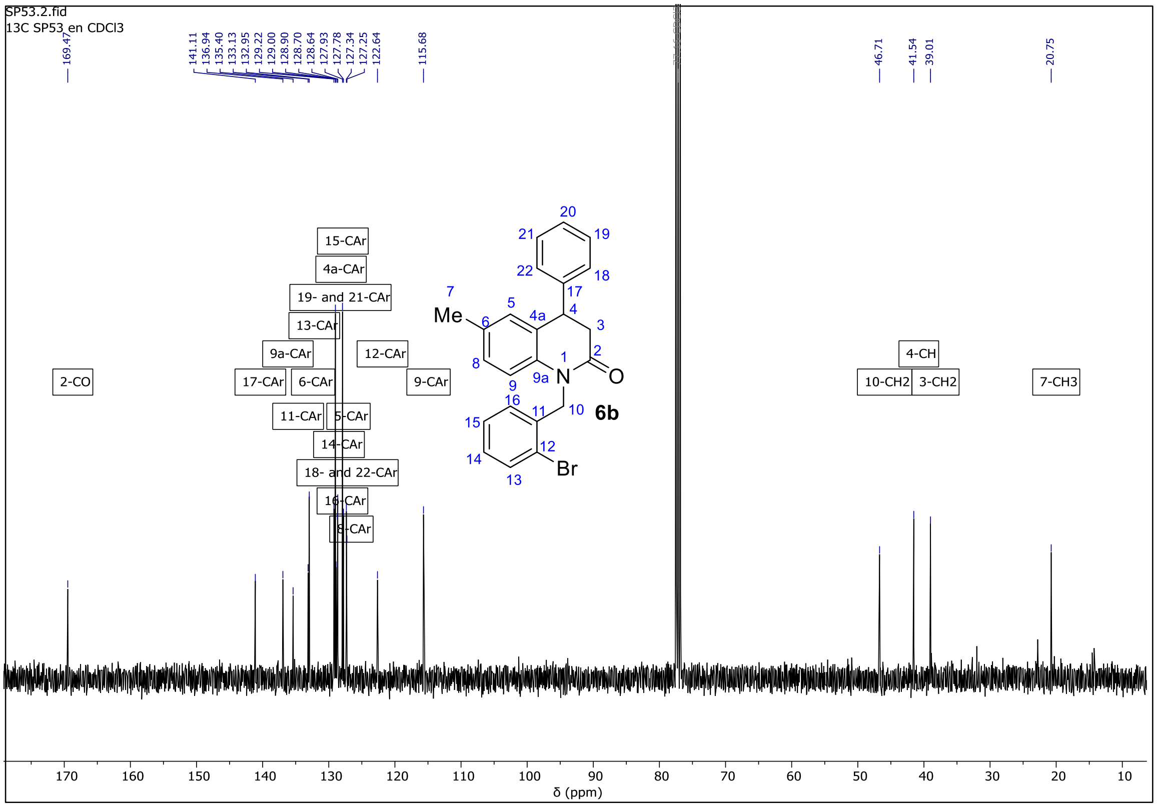
SP53.2.fid
13C SP53 en CDCl3
169.47
141.11
136.94
135.40
133.13
132.95
129.22
129.00
128.90
128.70
128.64
127.93
127.78
127.34
127.25
122.64
115.68
46.71
41.54
39.01
20.75
15-CAr
4a-CAr
19- and 21-CAr
13-CAr
9a-CAr
12-CAr
2-CO
17-CAr
6-CAr
9-CAr
11-CAr
5-CAr
14-CAr
18- and 22-CAr
16-CAr
8-CAr
4-CH
10-CH2
3-CH2
7-CH3
6b
δ (ppm)

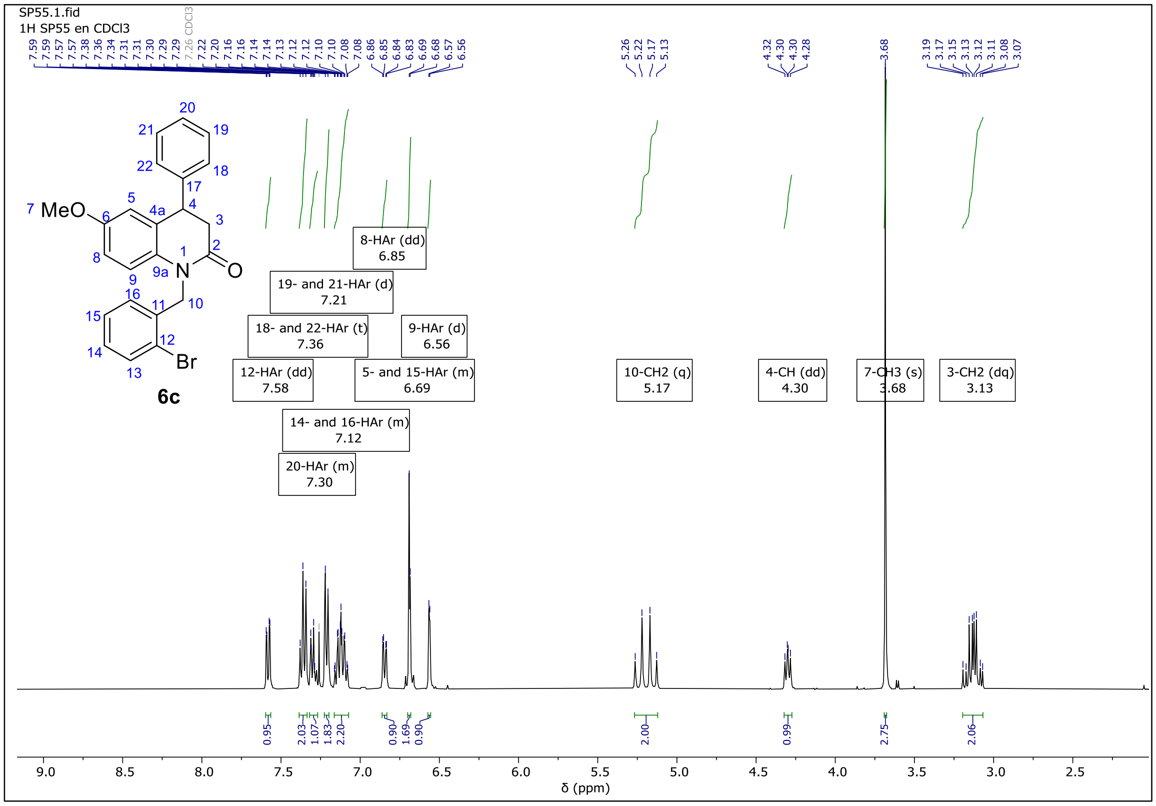
SP55.1.fid
1H SP55 en CDCl3
7.59 7.59 7.57 7.57 7.38 7.36 7.34 7.31 7.31 7.30 7.29 7.29 7.29 7.26 CDCl3 7.22 7.20 7.16 7.16 7.14 7.14 7.13 7.12 7.12 7.10 7.10 7.08 7.08 6.86 6.85 6.84 6.83 6.69 6.68 6.57 6.56
5.26 5.22 5.17 5.13
4.32 4.30 4.30 4.28
3.68
3.19 3.17 3.15 3.13 3.12 3.11 3.08 3.07
8-HAr (dd)
6.85
19- and 21-HAr (d)
7.21
18- and 22-HAr (t)
7.36
9-HAr (d)
6.56
12-HAr (dd)
7.58
5- and 15-HAr (m)
6.69
10-CH2 (q)
5.17
4-CH (dd)
4.30
7-CH3 (s)
3.68
3-CH2 (dq)
3.13
14- and 16-HAr (m)
7.12
20-HAr (m)
7.30
7 MeO
6c
Br
0.95 2.03 1.07 1.83 2.20 0.90 1.69 0.90 2.00 0.99 2.75 2.06
9.0 8.5 8.0 7.5 7.0 6.5 6.0 5.5 5.0 4.5 4.0 3.5 3.0 2.5
δ (ppm)

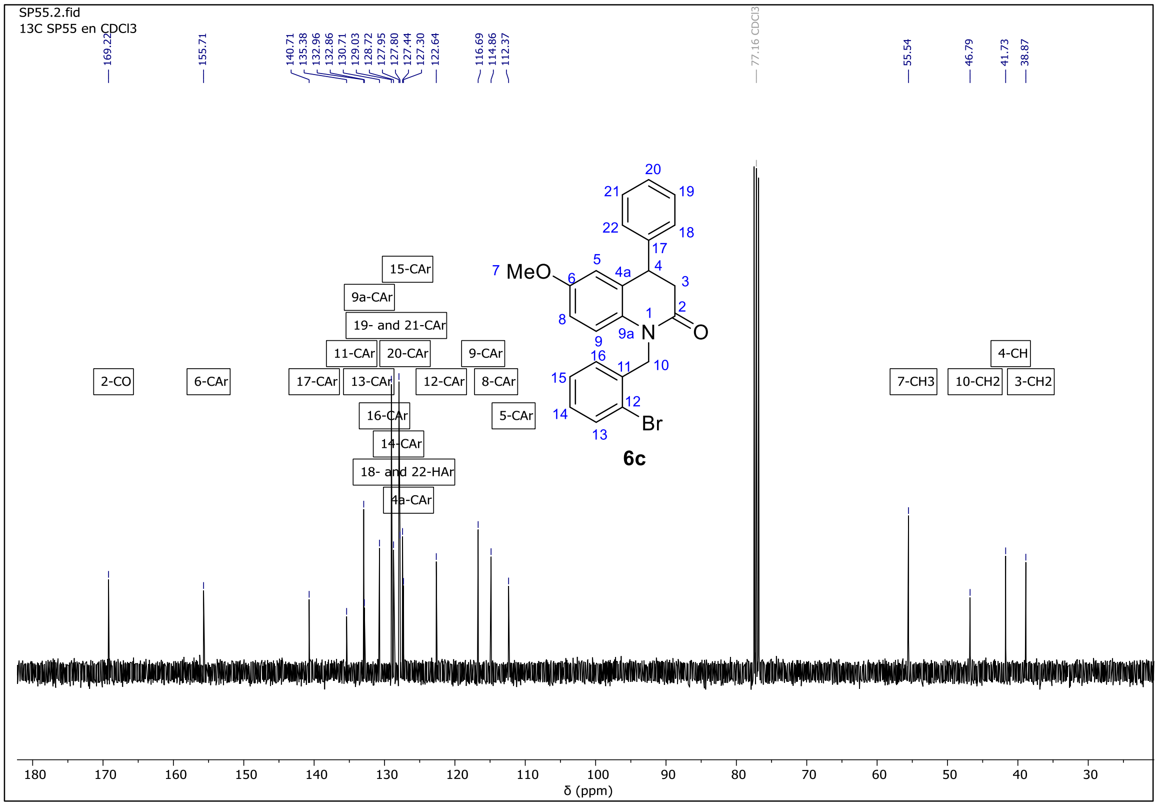
SP55.2.fid
13C SP55 en CDCl3
169.22 155.71 140.71 135.38 132.96 132.86 130.71 129.03 128.72 127.95 127.80 127.44 127.30 122.64 116.69 114.86 112.37 77.16 CDCl3 55.54 46.79 41.73 38.87
15-CAr
9a-CAr
19- and 21-CAr
11-CAr
20-CAr
9-CAr
4-CH
2-CO
6-CAr
17-CAr
13-CAr
12-CAr
8-CAr
7-CH3
10-CH2
3-CH2
16-CAr
5-CAr
14-CAr
18- and 22-HAr
4a-CAr
7 MeO
6c
Br
180 170 160 150 140 130 120 110 100 90 80 70 60 50 40 30
δ (ppm)

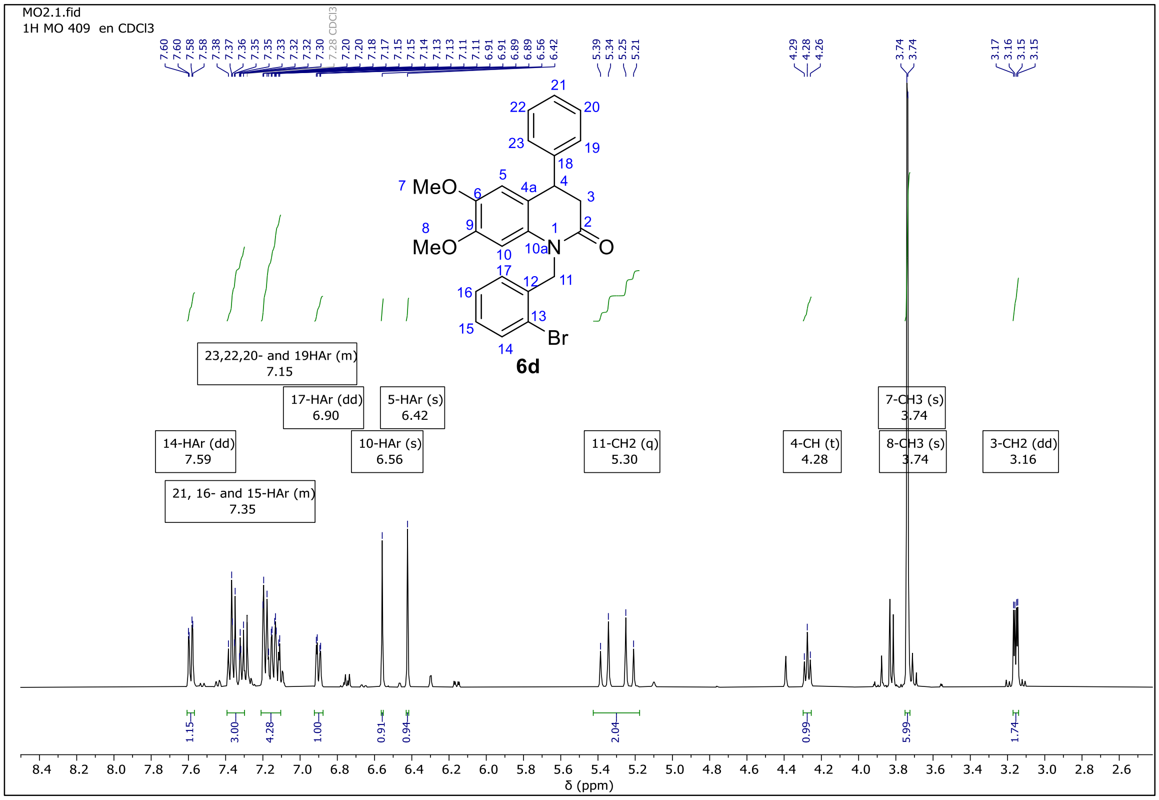
MO2.1.fid
1H MO 409 en CDCl3
6d
23,22,20- and 19HAr (m) 7.15
17-HAr (dd) 6.90
5-HAr (s) 6.42
14-HAr (dd) 7.59
10-HAr (s) 6.56
11-CH2 (q) 5.30
4-CH (t) 4.28
7-CH3 (s) 3.74
8-CH3 (s) 3.74
3-CH2 (dd) 3.16
21, 16- and 15-HAr (m) 7.35
δ (ppm)

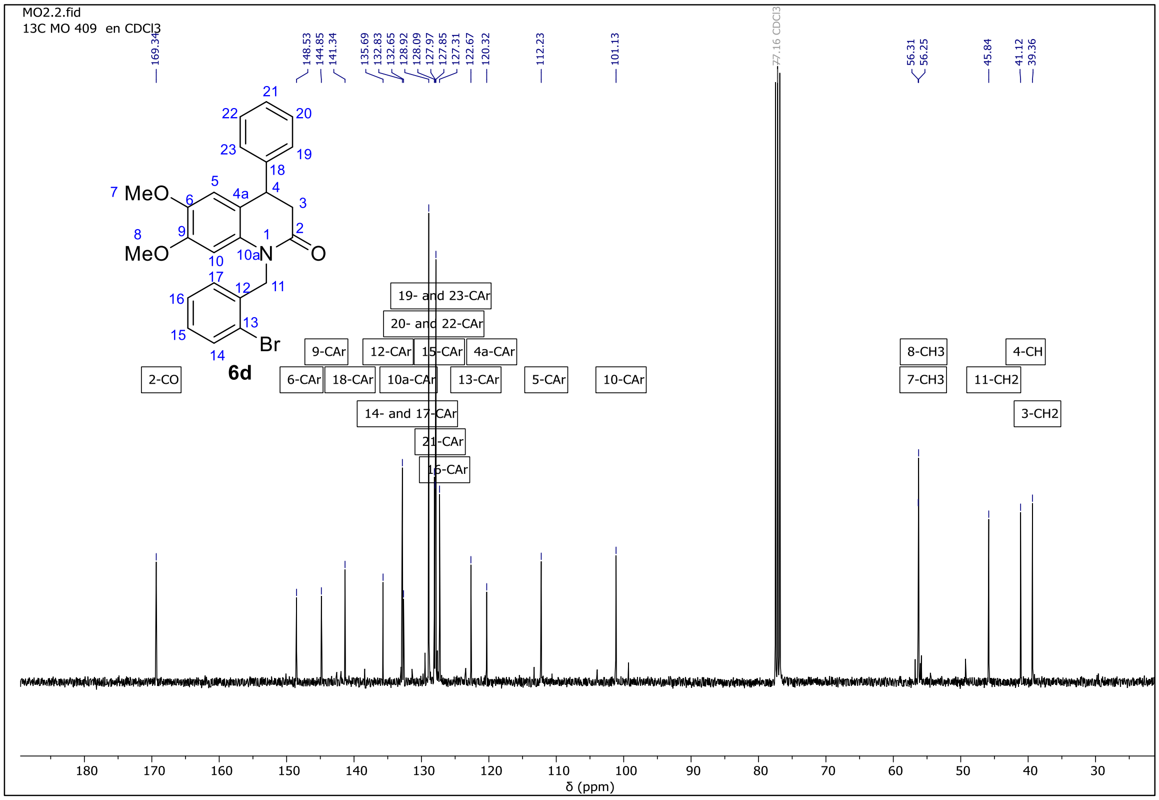
MO2.2.fid
13C MO 409 en CDCl3
6d
19- and 23-CAr
20- and 22-CAr
9-CAr
12-CAr
15-CAr
4a-CAr
8-CH3
4-CH
2-CO
6-CAr
18-CAr
10a-CAr
13-CAr
5-CAr
10-CAr
7-CH3
11-CH2
14- and 17-CAr
3-CH2
21-CAr
16-CAr
δ (ppm)

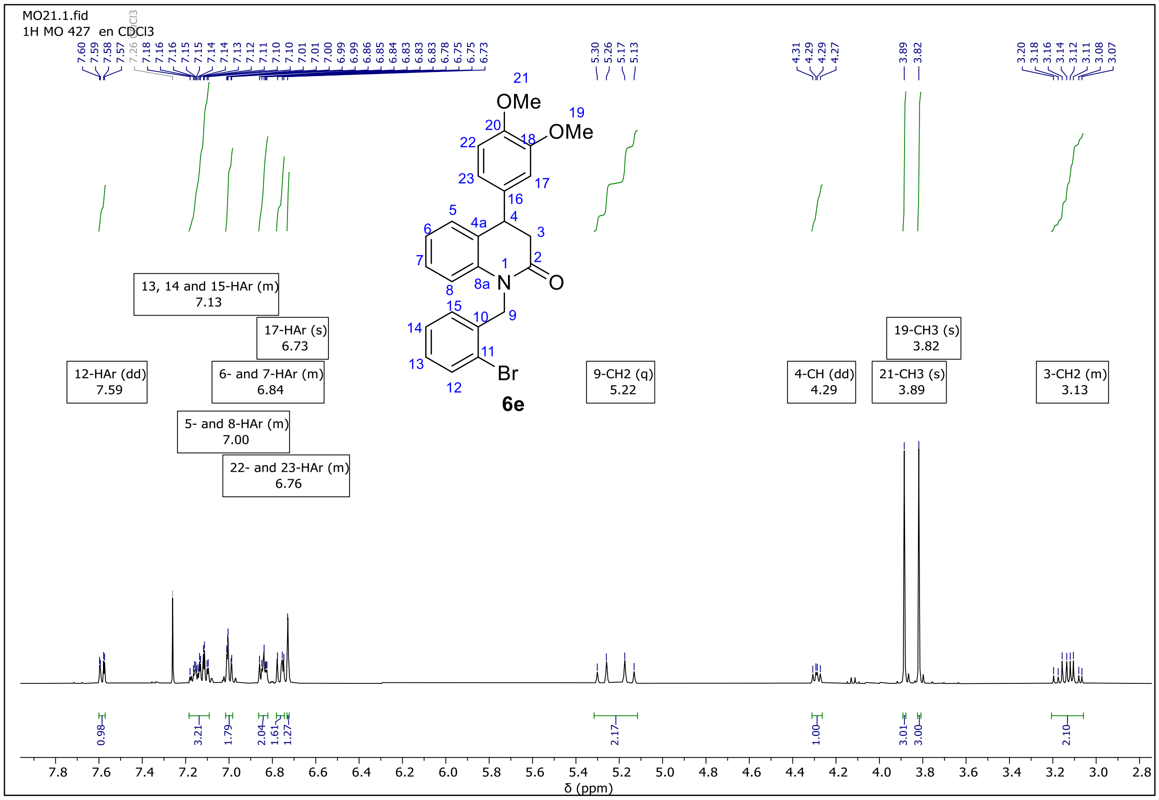
MO21.1.fid
1H MO 427 en CDCl3
13, 14 and 15-HAr (m)
7.13
17-HAr (s)
6.73
12-HAr (dd)
7.59
6- and 7-HAr (m)
6.84
5- and 8-HAr (m)
7.00
22- and 23-HAr (m)
6.76
9-CH2 (q)
5.22
4-CH (dd)
4.29
19-CH3 (s)
3.82
21-CH3 (s)
3.89
3-CH2 (m)
3.13
6e
δ (ppm)

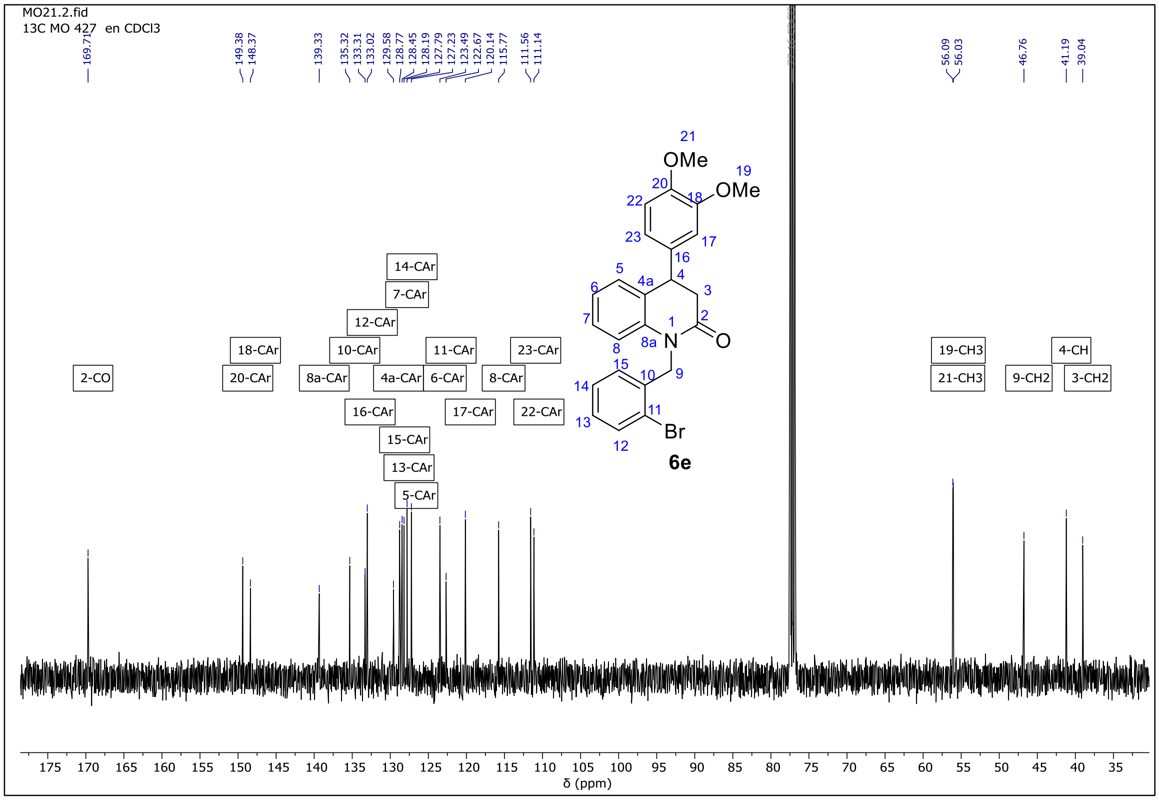
MO21.2.fid
13C MO 427 en CDCl3
2-CO
18-CAr
20-CAr
8a-CAr
10-CAr
12-CAr
7-CAr
14-CAr
4a-CAr
16-CAr
15-CAr
13-CAr
5-CAr
6-CAr
11-CAr
17-CAr
8-CAr
23-CAr
22-CAr
19-CH3
21-CH3
9-CH2
4-CH
3-CH2
6e
δ (ppm)

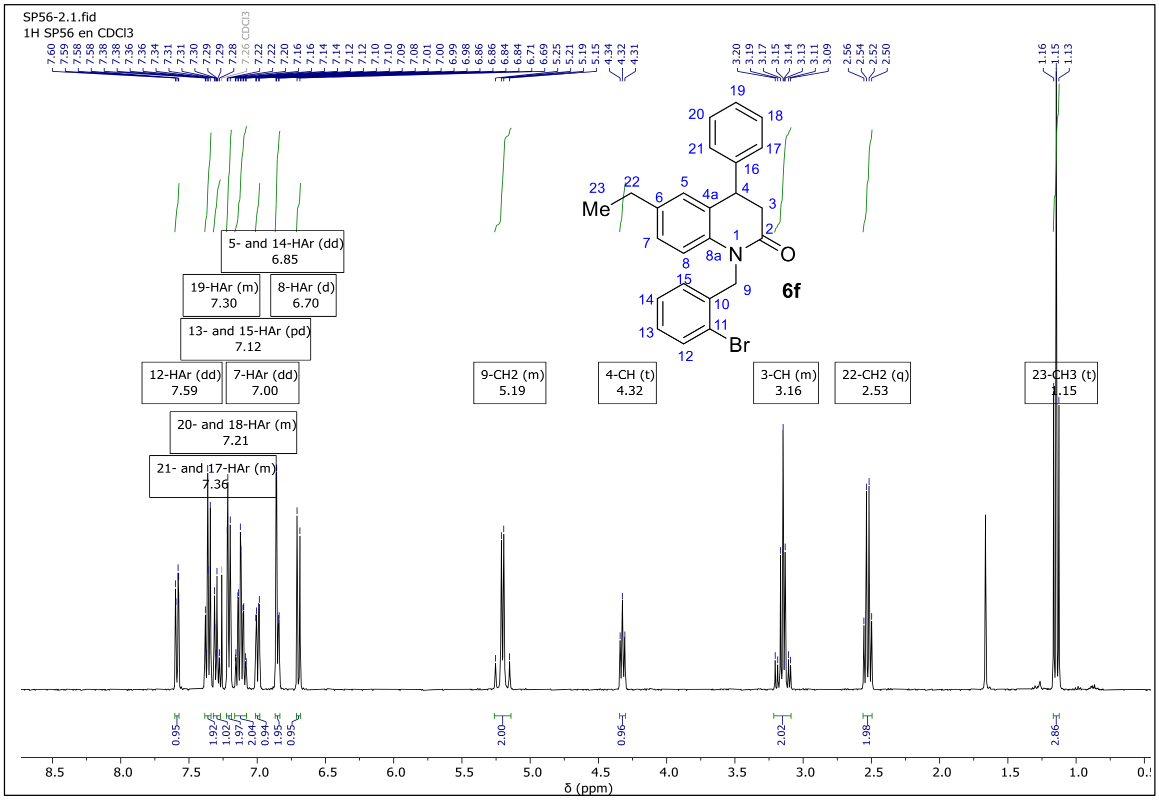
SP56-2.1.fid
1H SP56 en CDCl3
6f
5- and 14-HAr (dd) 6.85
19-HAr (m) 7.30
8-HAr (d) 6.70
13- and 15-HAr (pd) 7.12
12-HAr (dd) 7.59
7-HAr (dd) 7.00
20- and 18-HAr (m) 7.21
21- and 17-HAr (m) 7.36
9-CH2 (m) 5.19
4-CH (t) 4.32
3-CH (m) 3.16
22-CH2 (q) 2.53
23-CH3 (t) 1.15
δ (ppm)

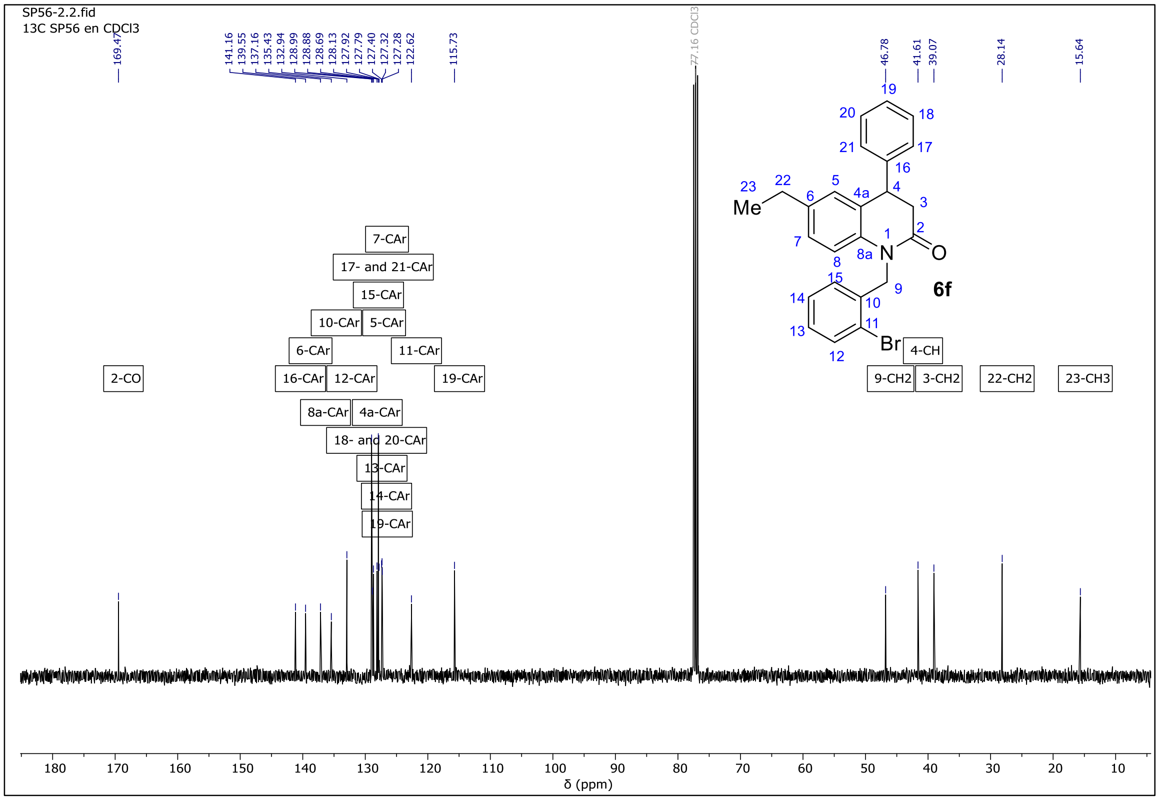
SP56-2.2.fid
13C SP56 en CDCl3
169.47
141.16
139.55
137.16
135.43
132.94
128.99
128.88
128.69
128.13
127.92
127.79
127.40
127.32
127.28
122.62
115.73
77.16 CDCl3
46.78
41.61
39.07
28.14
15.64
6f
7-CAr
17- and 21-CAr
15-CAr
10-CAr
5-CAr
6-CAr
11-CAr
2-CO
16-CAr
12-CAr
19-CAr
8a-CAr
4a-CAr
18- and 20-CAr
13-CAr
14-CAr
19-CAr
4-CH
9-CH2
3-CH2
22-CH2
23-CH3
δ (ppm)

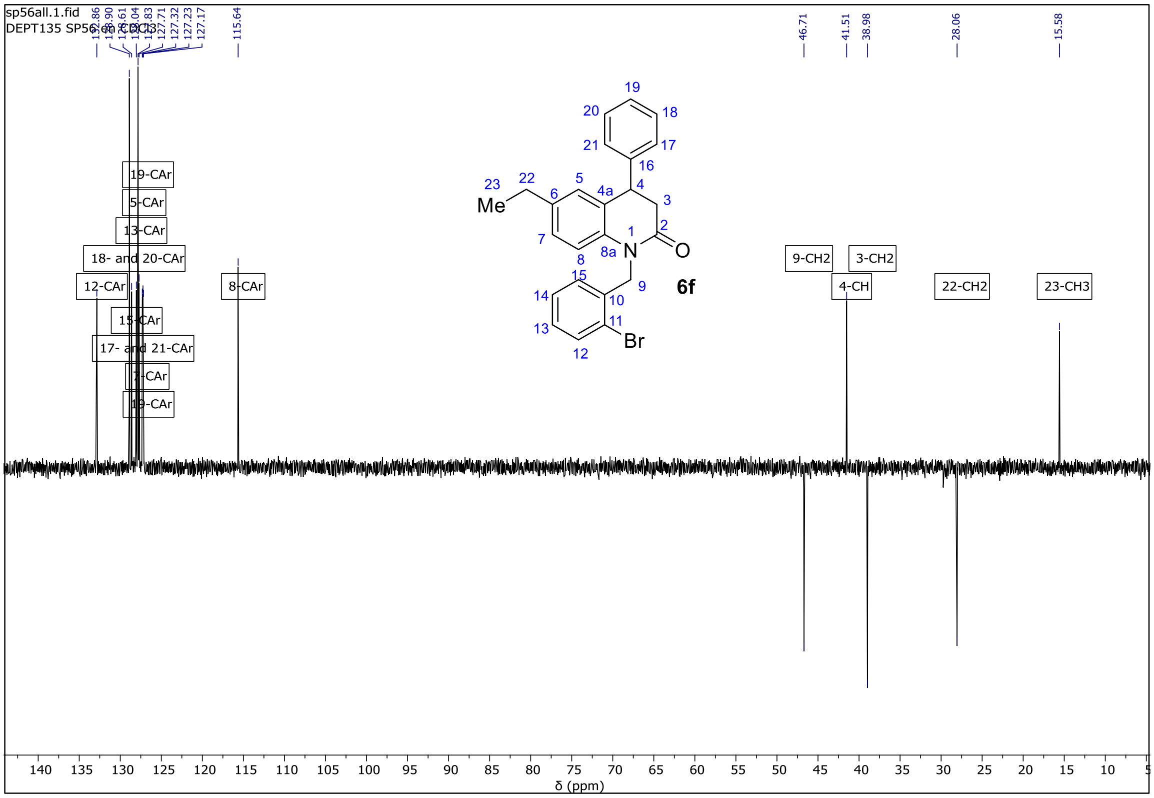
sp56all.1.fid
DEPT135 SP56 en CDCl3
115.64
46.71
41.51
38.98
28.06
15.58
19-CAr
5-CAr
13-CAr
18- and 20-CAr
12-CAr
8-CAr
15-CAr
17- and 21-CAr
7-CAr
9-CH2
3-CH2
4-CH
22-CH2
23-CH3
Me
N
O
Br
6f
δ (ppm)

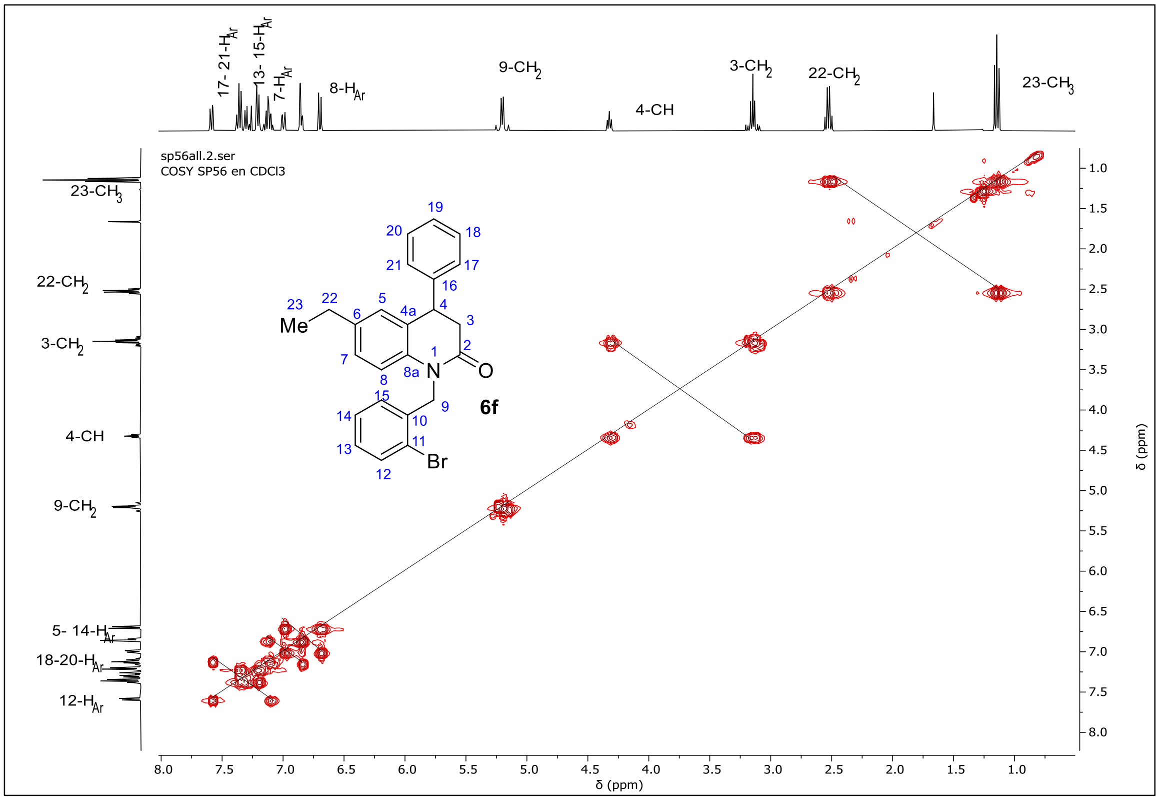
sp56all.2.ser
COSY SP56 en CDCl3
17- 21-H$_{Ar}$
13- 15-H$_{Ar}$
7-H$_{Ar}$
8-H$_{Ar}$
9-CH$_2$
4-CH
3-CH$_2$
22-CH$_2$
23-CH$_3$
5- 14-H$_{Ar}$
18-20-H$_{Ar}$
12-H$_{Ar}$
Me
N
O
Br
6f
δ (ppm)

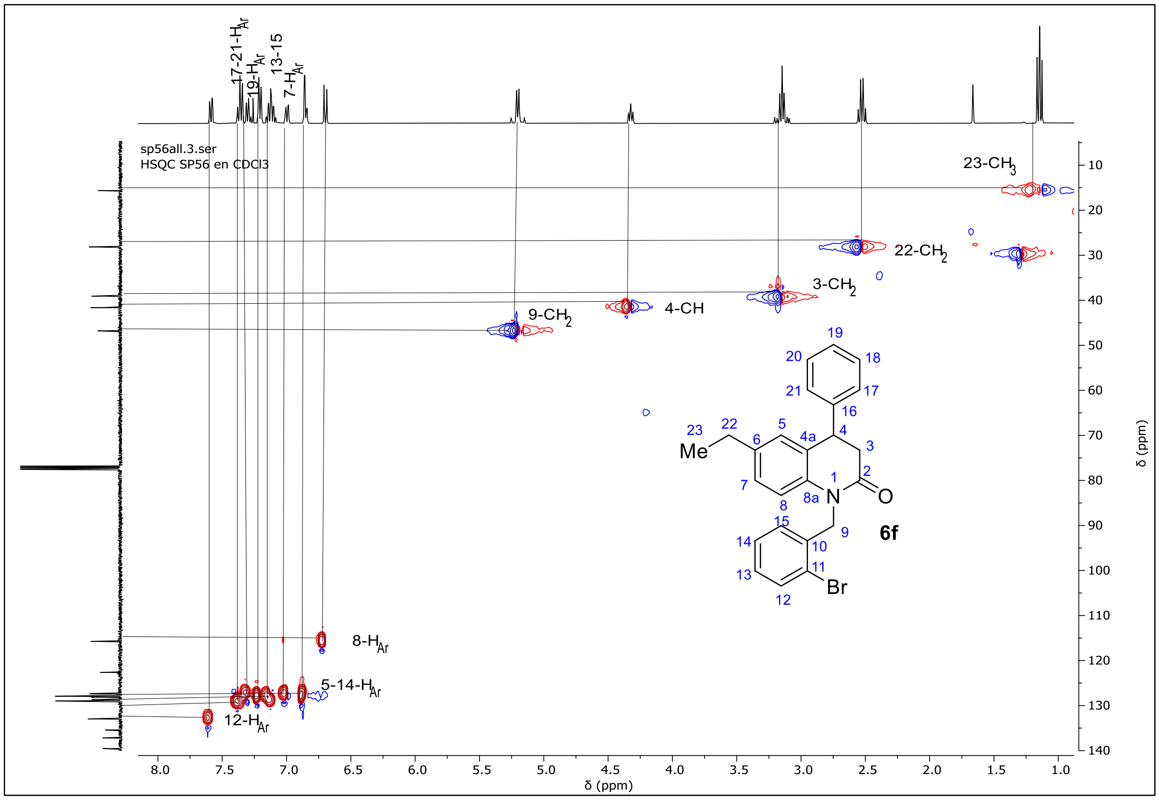
sp56all.3.ser
HSQC SP56 en CDCl3
17-21-H$_{Ar}$
19-H$_{Ar}$
13-15
7-H$_{Ar}$
23-CH$_3$
22-CH$_2$
3-CH$_2$
4-CH
9-CH$_2$
8-H$_{Ar}$
5-14-H$_{Ar}$
12-H$_{Ar}$
6f
Me
Br
O
N
δ (ppm)

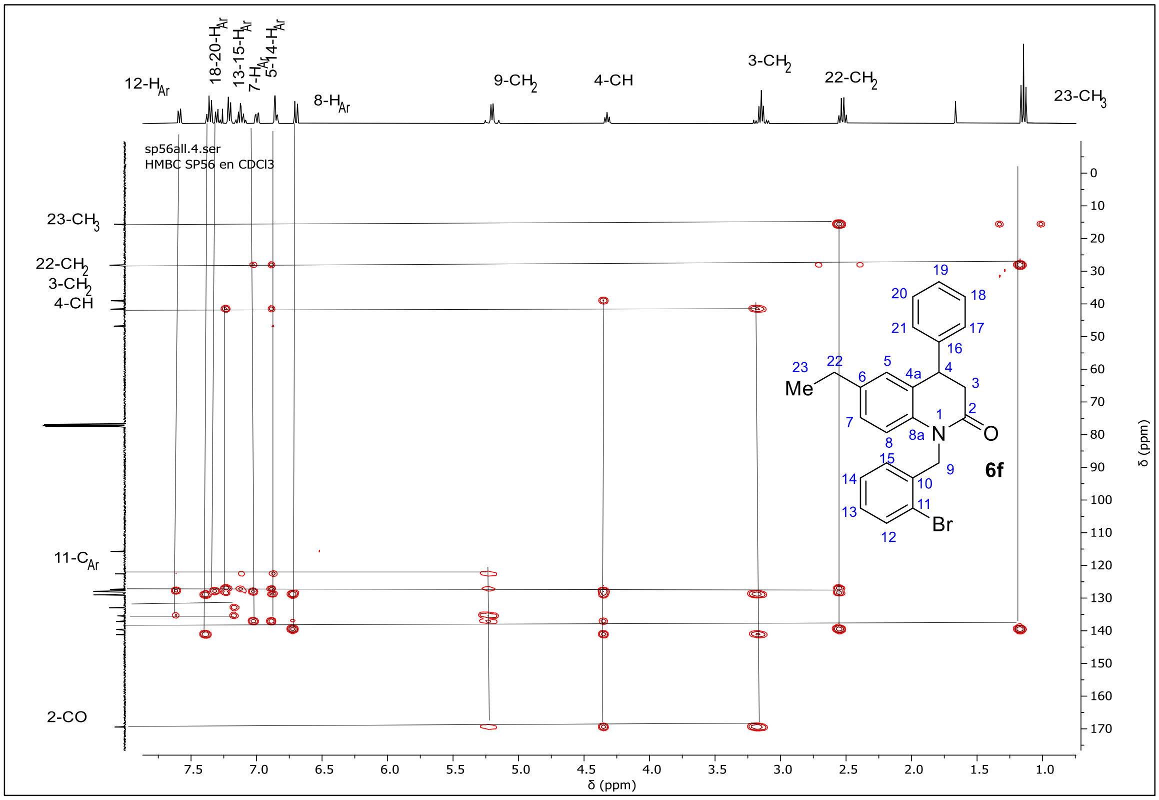
sp56all.4.ser
HMBC SP56 en CDCl3
12-H$_{Ar}$
18-20-H$_{Ar}$
13-15-H$_{Ar}$
7-H$_{Ar}$
5-14-H$_{Ar}$
8-H$_{Ar}$
9-CH$_2$
4-CH
3-CH$_2$
22-CH$_2$
23-CH$_3$
11-C$_{Ar}$
2-CO
6f
Me
Br
O
N
δ (ppm)

10. Figures S50-S66 of the respective ^{1}H and ^{13}C NMR spectra of all synthesized pyrido[3,2,1-*de*]phenanthridin-6-ones 4a-f

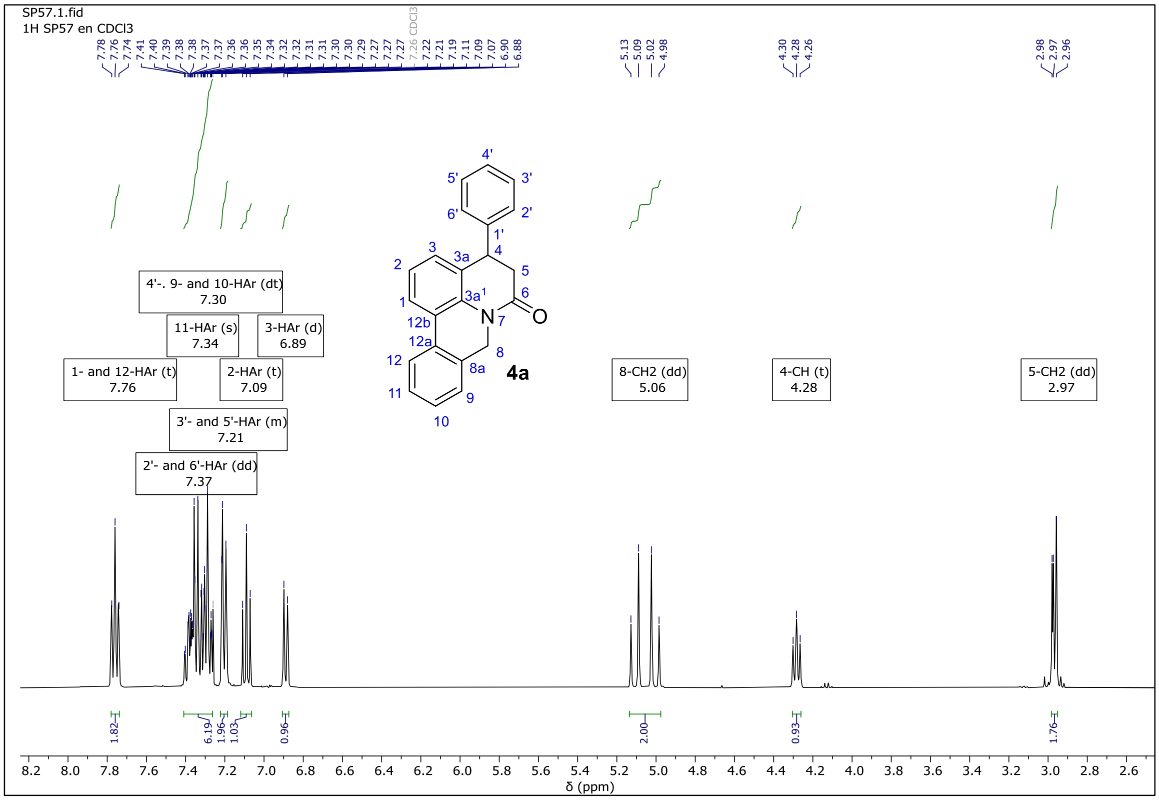

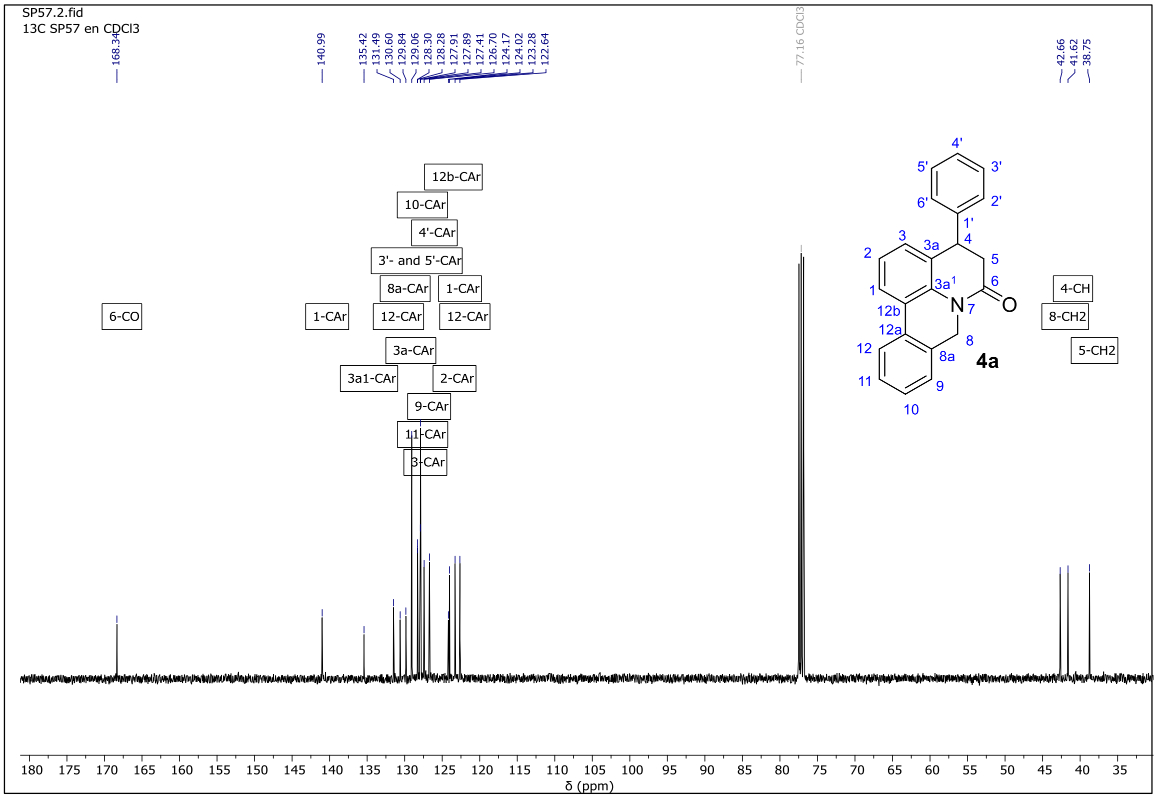

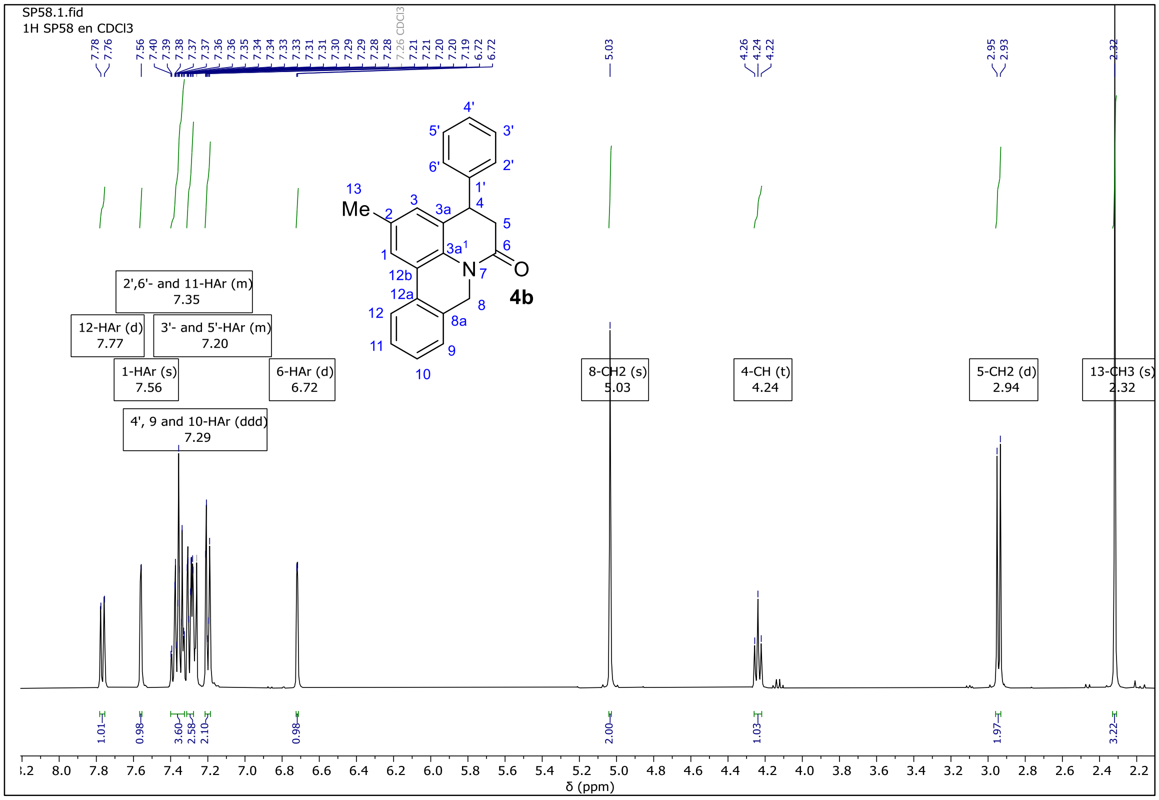
SP58.1.fid
1H SP58 en CDCl3
2',6'- and 11-HAr (m)
7.35
12-HAr (d)
7.77
3'- and 5'-HAr (m)
7.20
1-HAr (s)
7.56
6-HAr (d)
6.72
4', 9 and 10-HAr (ddd)
7.29
8-CH2 (s)
5.03
4-CH (t)
4.24
5-CH2 (d)
2.94
13-CH3 (s)
2.32
4b
δ (ppm)

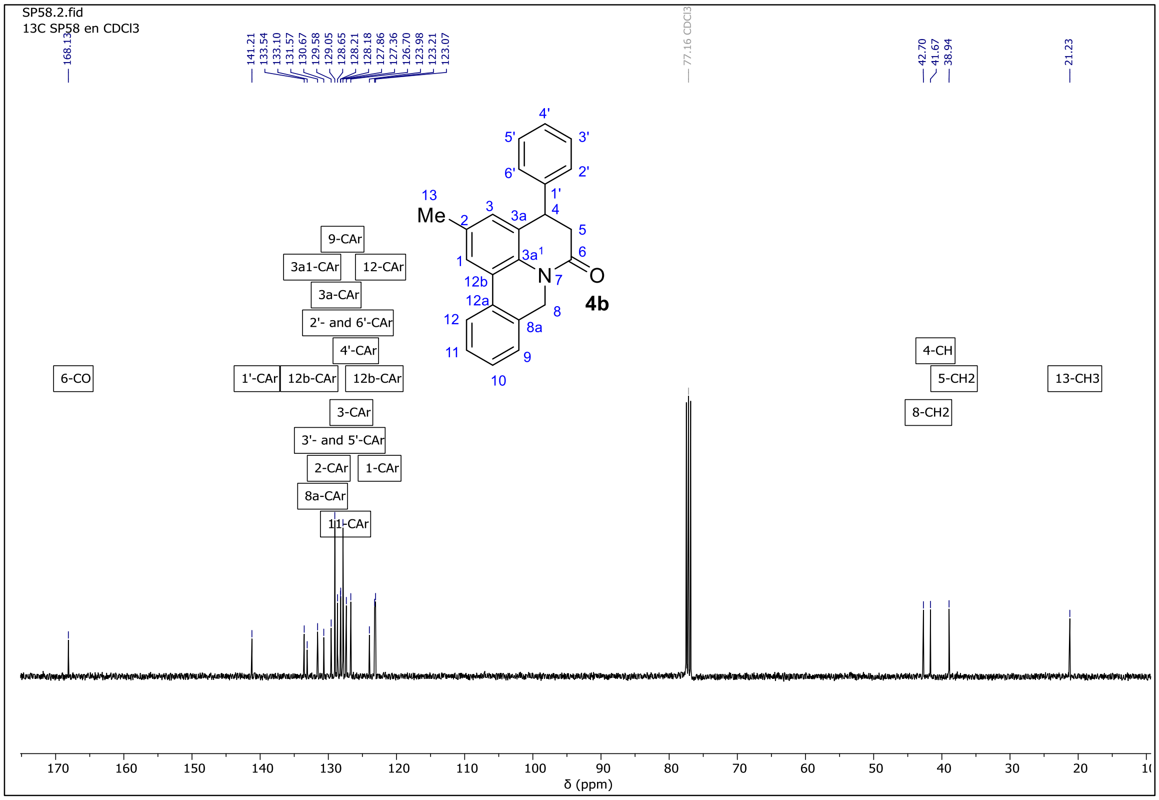
SP58.2.fid
13C SP58 en CDCl3
6-CO
9-CAr
3a1-CAr
12-CAr
3a-CAr
2'- and 6'-CAr
4'-CAr
1'-CAr
12b-CAr
12b-CAr
3-CAr
3'- and 5'-CAr
2-CAr
1-CAr
8a-CAr
11-CAr
4-CH
5-CH2
8-CH2
13-CH3
4b
δ (ppm)

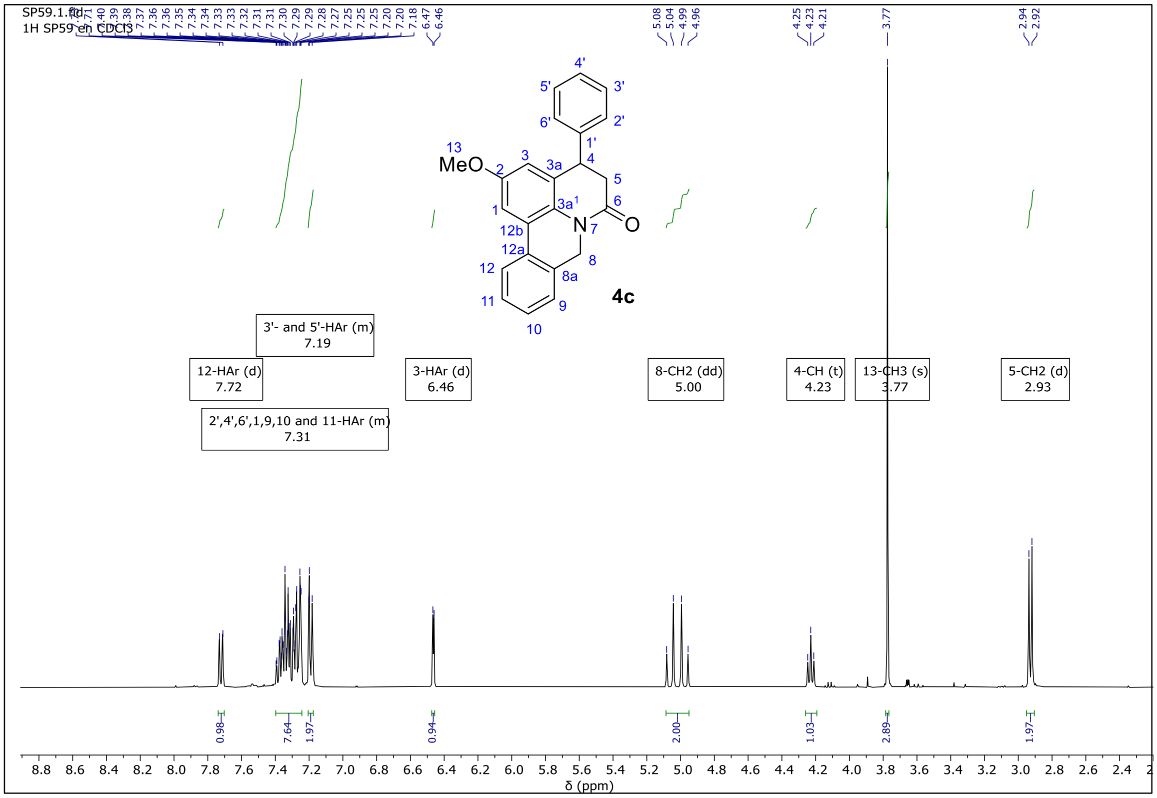
SP59.1.fid
1H SP59 en CDCl3
4c
3'- and 5'-HAr (m)
7.19
12-HAr (d)
7.72
3-HAr (d)
6.46
8-CH2 (dd)
5.00
4-CH (t)
4.23
13-CH3 (s)
3.77
5-CH2 (d)
2.93
2',4',6',1,9,10 and 11-HAr (m)
7.31
δ (ppm)

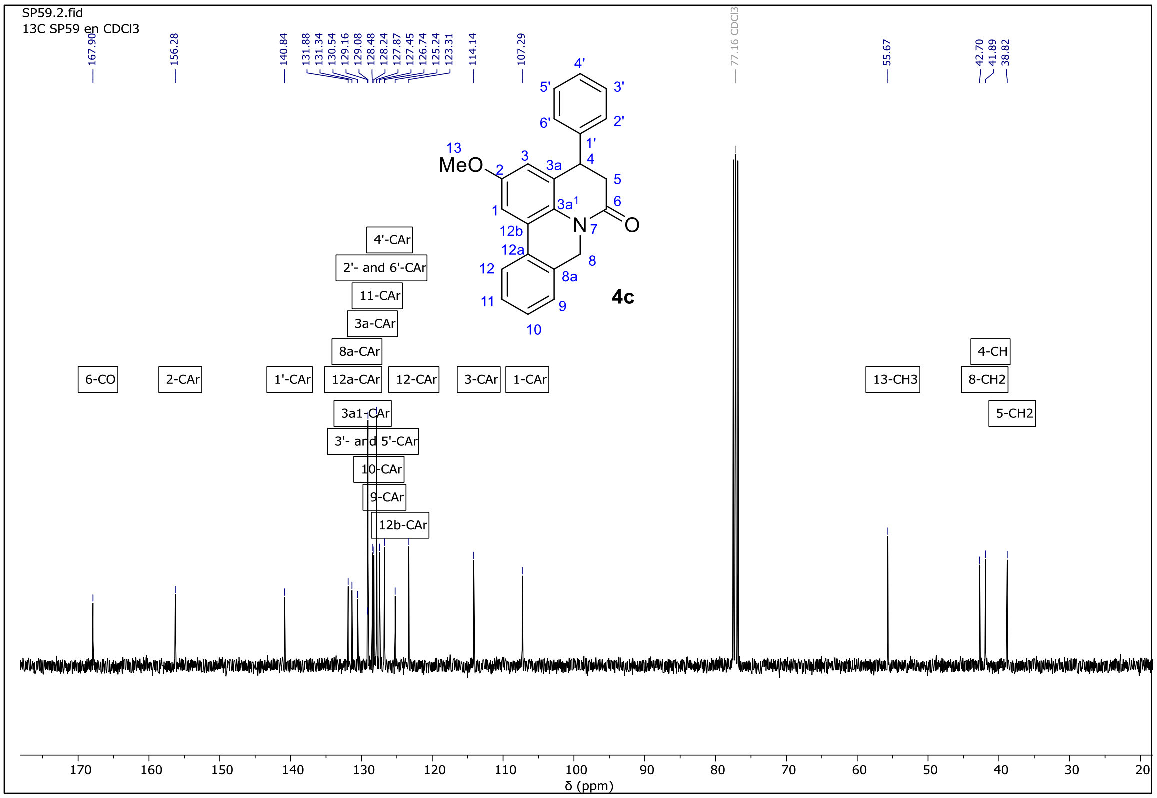
SP59.2.fid
13C SP59 en CDCl3
4c
4'-CAr
2'- and 6'-CAr
11-CAr
3a-CAr
8a-CAr
6-CO
2-CAr
1'-CAr
12a-CAr
12-CAr
3-CAr
1-CAr
13-CH3
4-CH
8-CH2
5-CH2
3a1-CAr
3'- and 5'-CAr
10-CAr
9-CAr
12b-CAr
δ (ppm)

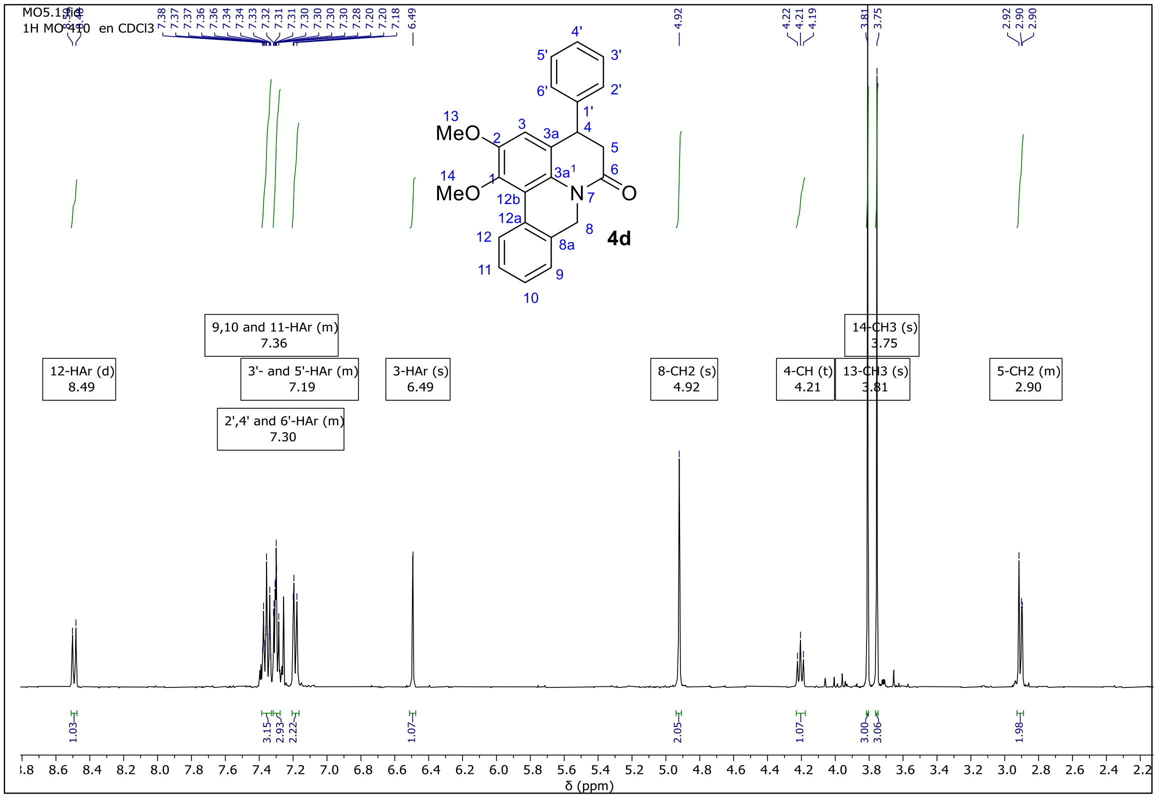
MO5.1.fid
1H MO 410 en CDCl3
4d
12-HAr (d)
8.49
9,10 and 11-HAr (m)
7.36
3'- and 5'-HAr (m)
7.19
2',4' and 6'-HAr (m)
7.30
3-HAr (s)
6.49
8-CH2 (s)
4.92
4-CH (t)
4.21
14-CH3 (s)
3.75
13-CH3 (s)
3.81
5-CH2 (m)
2.90
δ (ppm)

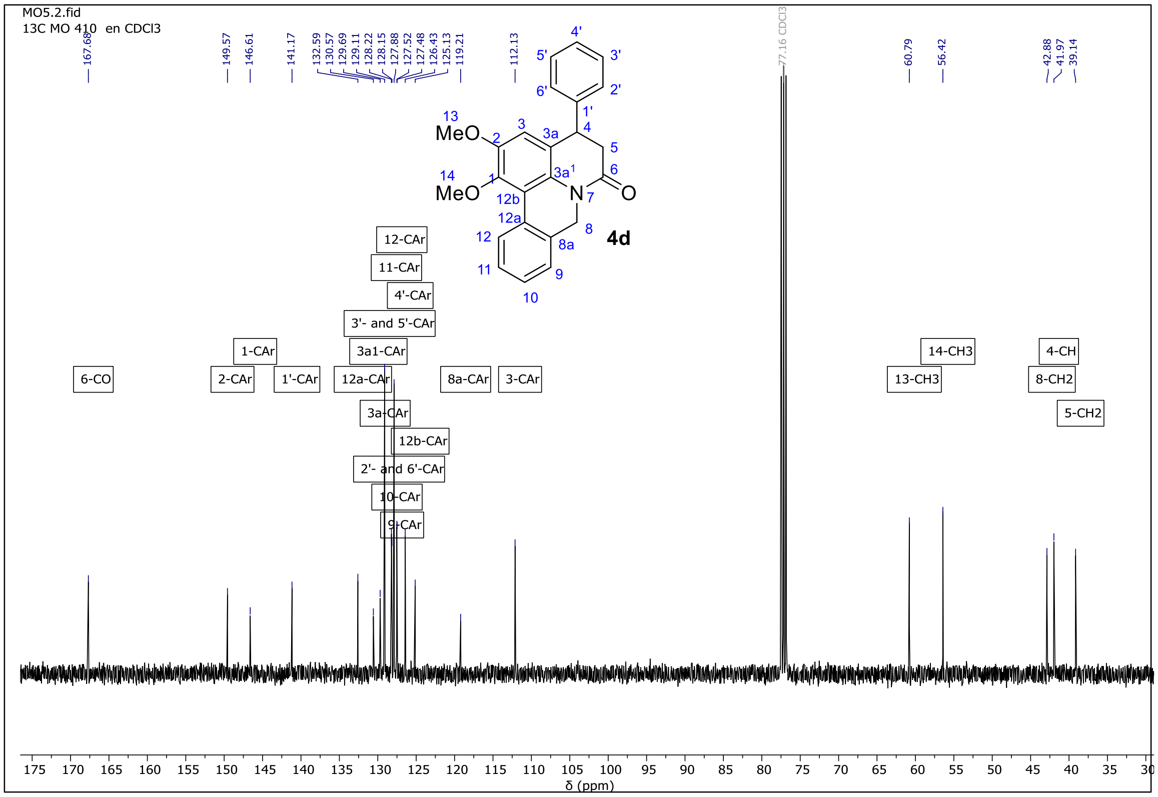
MO5.2.fid
13C MO 410 en CDCl3
4d
6-CO
2-CAr
1-CAr
1'-CAr
12a-CAr
3a1-CAr
3'- and 5'-CAr
4'-CAr
11-CAr
12-CAr
3a-CAr
12b-CAr
2'- and 6'-CAr
10-CAr
9-CAr
8a-CAr
3-CAr
13-CH3
14-CH3
4-CH
8-CH2
5-CH2
δ (ppm)

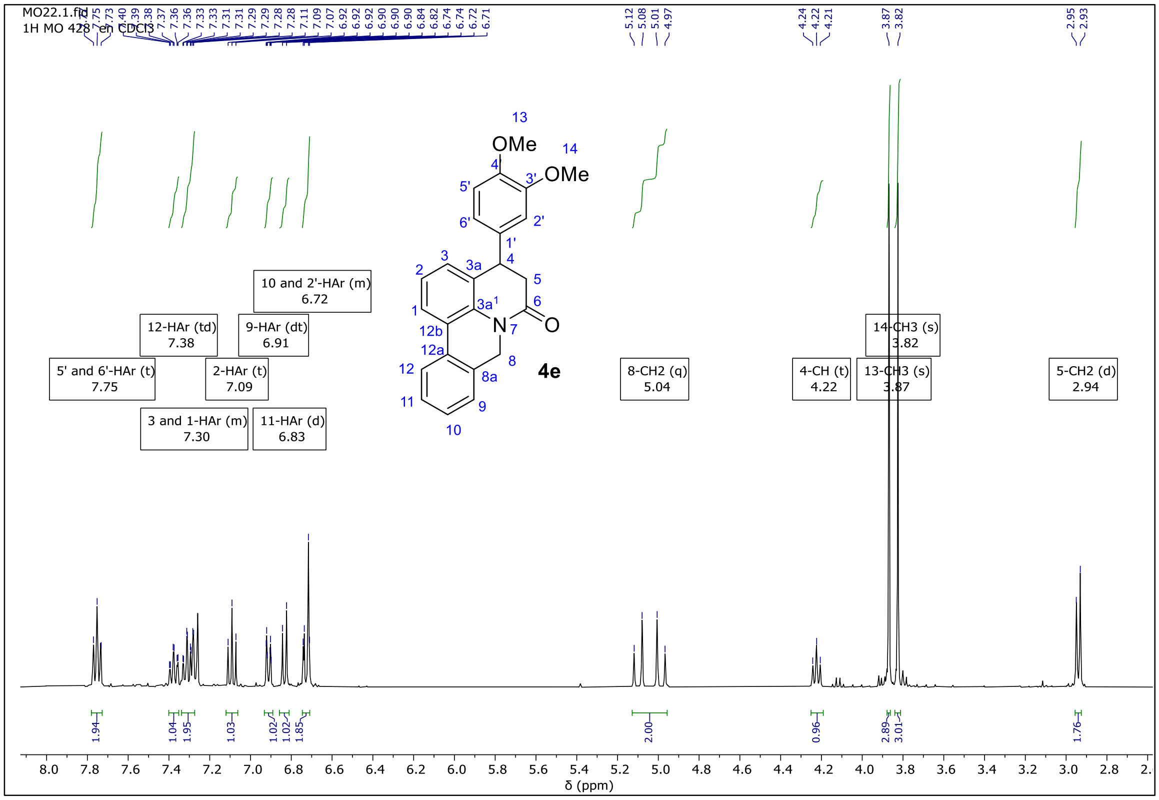
MO22.1.fid
1H MO 428 en CDCl3
7.75 7.73 7.40 7.39 7.38 7.37 7.36 7.36 7.33 7.33 7.31 7.31 7.29 7.29 7.28 7.28 7.11 7.09 7.07 6.92 6.92 6.92 6.90 6.90 6.90 6.84 6.82 6.74 6.74 6.72 6.71
5.12 5.08 5.01 4.97
4.24 4.22 4.21
3.87 3.82
2.95 2.93
4e
10 and 2'-HAr (m) 6.72
12-HAr (td) 7.38
9-HAr (dt) 6.91
5' and 6'-HAr (t) 7.75
2-HAr (t) 7.09
3 and 1-HAr (m) 7.30
11-HAr (d) 6.83
8-CH2 (q) 5.04
4-CH (t) 4.22
14-CH3 (s) 3.82
13-CH3 (s) 3.87
5-CH2 (d) 2.94
1.94 1.04 1.95 1.03 1.02 1.02 1.85 2.00 0.96 2.89 3.01 1.76
δ (ppm)

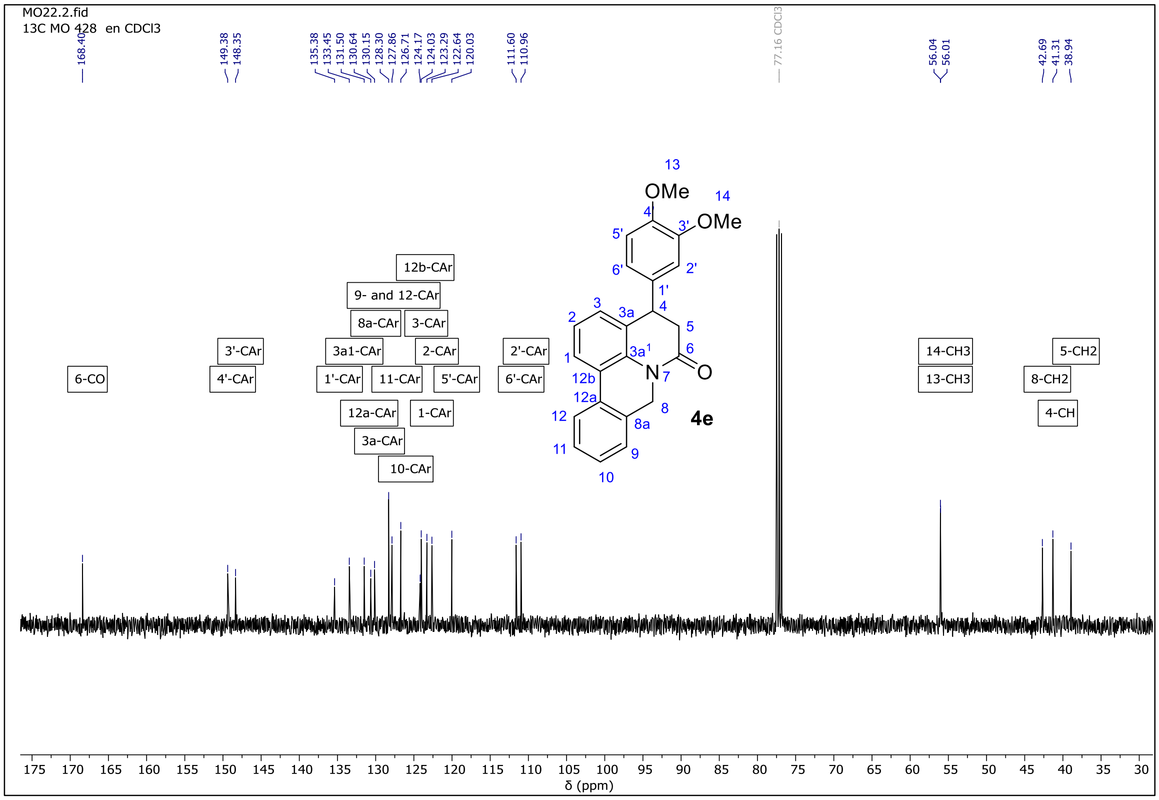
MO22.2.fid
13C MO 428 en CDCl3
168.40
149.38 148.35
135.38 133.45 131.50 130.64 130.15 128.30 127.86 126.71 124.17 124.03 123.29 122.64 120.03
111.60 110.96
77.16 CDCl3
56.04 56.01
42.69 41.31 38.94
4e
12b-CAr
9- and 12-CAr
8a-CAr
3-CAr
3'-CAr
3a1-CAr
2-CAr
2'-CAr
6-CO
4'-CAr
1'-CAr
11-CAr
5'-CAr
6'-CAr
12a-CAr
1-CAr
3a-CAr
10-CAr
14-CH3
13-CH3
5-CH2
8-CH2
4-CH
δ (ppm)

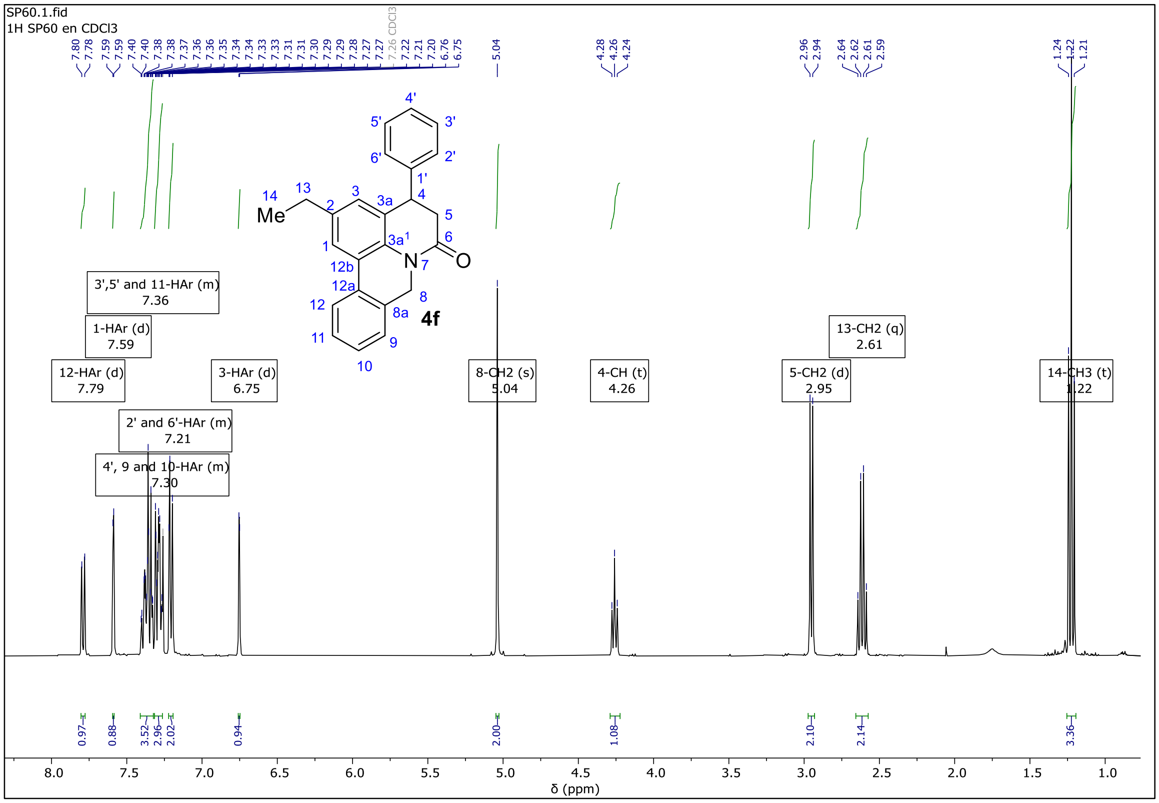
SP60.1.fid
1H SP60 en CDCl3
7.80 7.78 7.59 7.59 7.40 7.38 7.38 7.37 7.36 7.36 7.35 7.34 7.34 7.33 7.33 7.31 7.31 7.30 7.29 7.29 7.28 7.27 7.27 7.26 CDCl3 7.22 7.21 7.20 6.76 6.75
5.04
4.28 4.26 4.24
2.96 2.94 2.64 2.62 2.61 2.59
1.24 1.22 1.21
4f
3',5' and 11-HAr (m) 7.36
1-HAr (d) 7.59
12-HAr (d) 7.79
3-HAr (d) 6.75
2' and 6'-HAr (m) 7.21
4', 9 and 10-HAr (m) 7.30
8-CH2 (s) 5.04
4-CH (t) 4.26
13-CH2 (q) 2.61
5-CH2 (d) 2.95
14-CH3 (t) 1.22
0.97 0.88 3.52 2.96 2.02 0.94 2.00 1.08 2.10 2.14 3.36
δ (ppm)

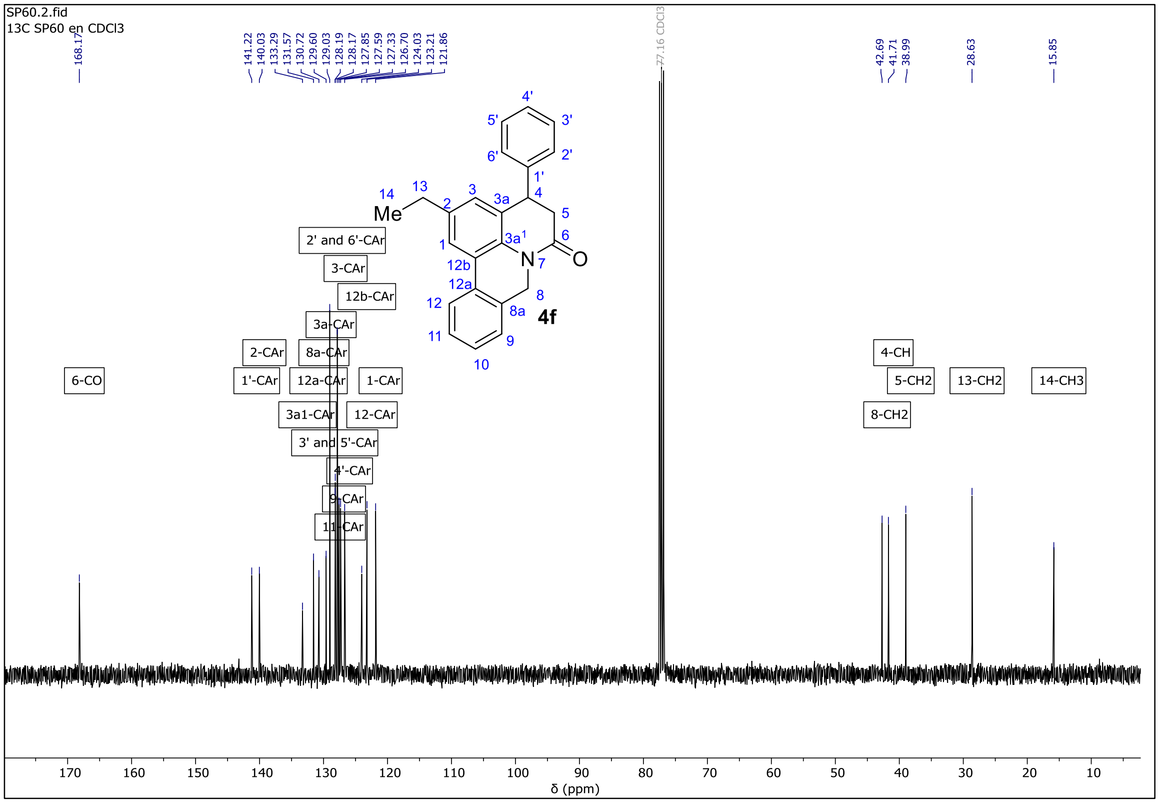
SP60.2.fid
13C SP60 en CDCl3
168.17
141.22 140.03 133.29 131.57 130.72 129.60 129.03 128.19 128.17 127.85 127.59 127.33 126.70 124.03 123.21 121.86
77.16 CDCl3
42.69 41.71 38.99 28.63 15.85
4f
2' and 6'-CAr
3-CAr
12b-CAr
3a-CAr
2-CAr
8a-CAr
6-CO
1'-CAr
12a-CAr
1-CAr
3a1-CAr
12-CAr
3' and 5'-CAr
4'-CAr
9-CAr
11-CAr
4-CH
5-CH2
13-CH2
14-CH3
8-CH2
δ (ppm)

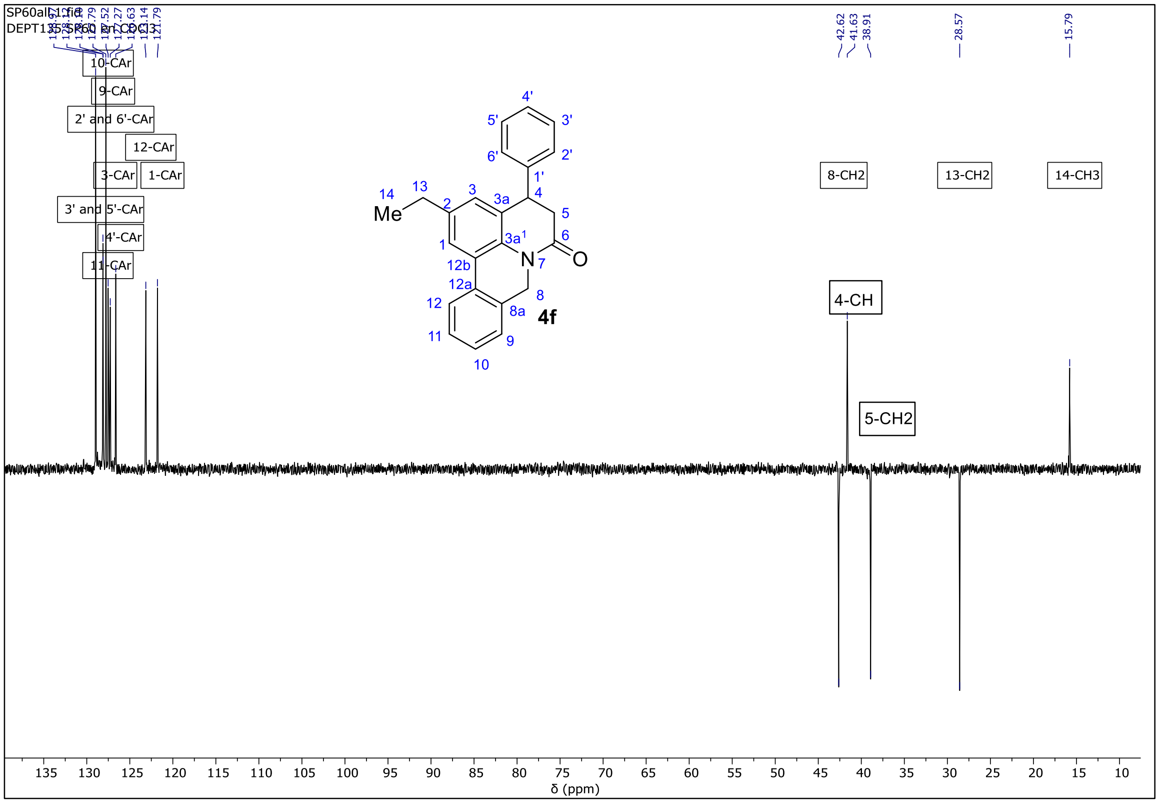
DEPT135 SP60 en CDCl3
42.62
41.63
38.91
28.57
15.79
10-CAr
9-CAr
2' and 6'-CAr
12-CAr
3-CAr
1-CAr
3' and 5'-CAr
4'-CAr
11-CAr
8-CH2
13-CH2
14-CH3
4-CH
5-CH2
4f
δ (ppm)

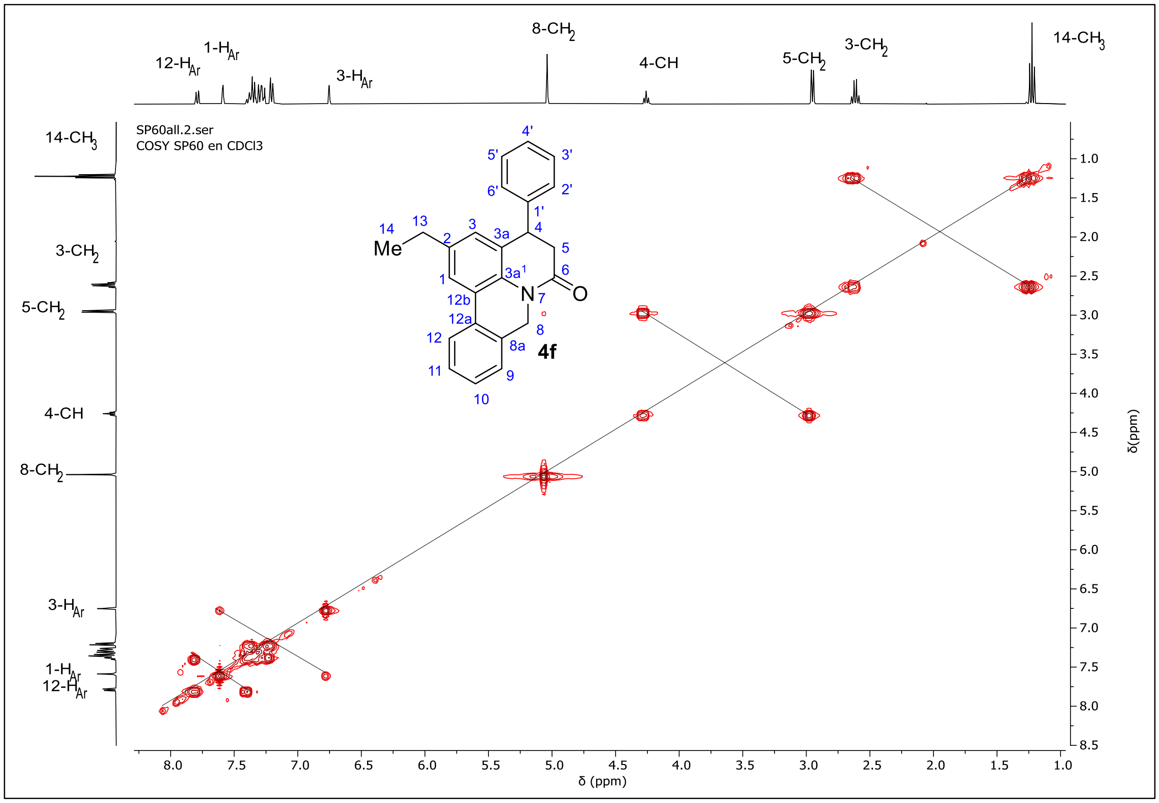
SP60all.2.ser
COSY SP60 en CDCl3
12-H$_{Ar}$
1-H$_{Ar}$
3-H$_{Ar}$
8-CH$_2$
4-CH
5-CH$_2$
3-CH$_2$
14-CH$_3$
4f
δ (ppm)

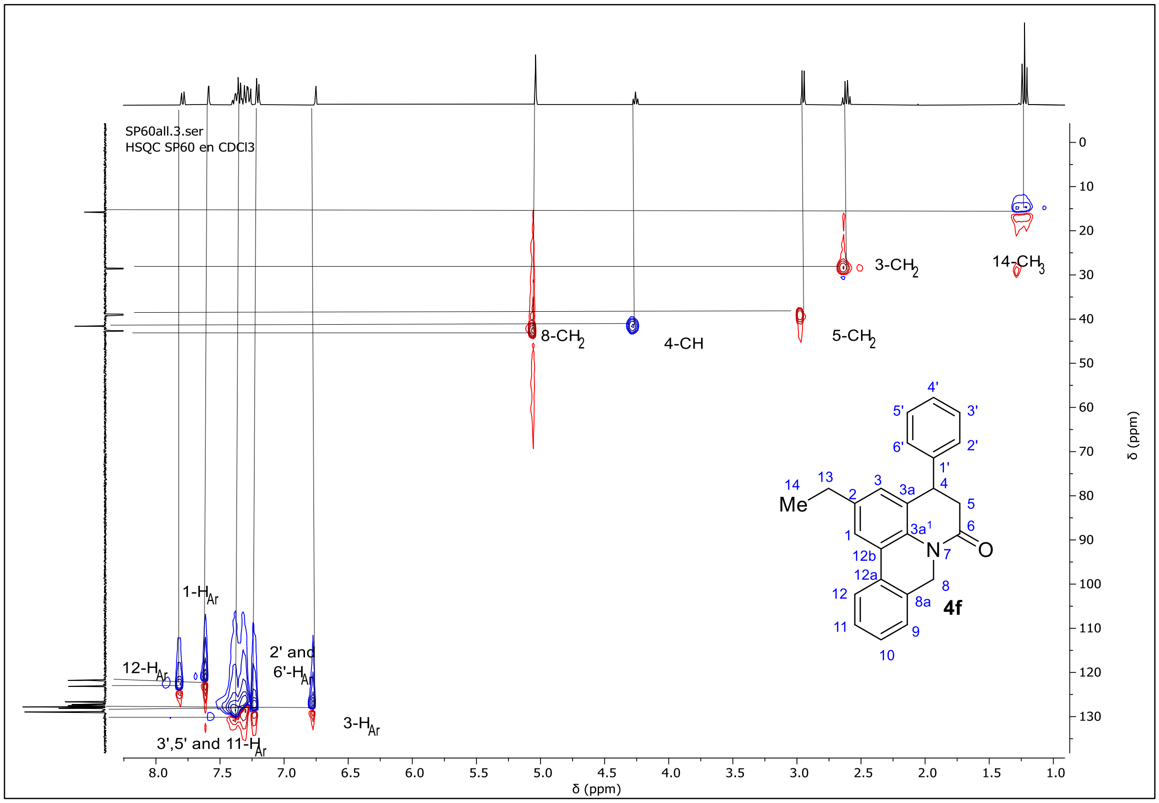
SP60all.3.ser
HSQC SP60 en CDCl3
3-CH2
14-CH3
8-CH2
4-CH
5-CH2
1-HAr
12-HAr
2' and 6'-HAr
3-HAr
3',5' and 11-HAr
4f
δ (ppm)

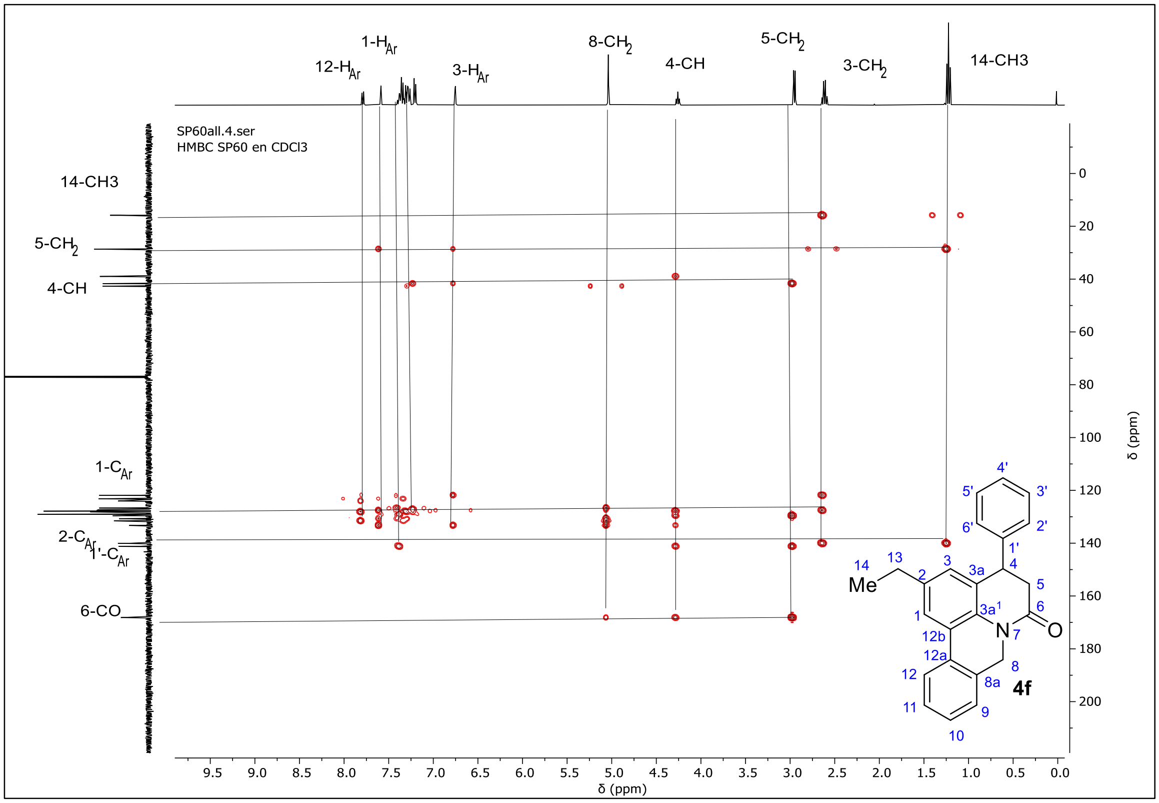
SP60all.4.ser
HMBC SP60 en CDCl3
1-HAr
12-HAr
3-HAr
8-CH2
4-CH
5-CH2
3-CH2
14-CH3
5-CH2
4-CH
1-CAr
2-CAr
1'-CAr
6-CO
4f
δ (ppm)
